# Unveiling the role of stress hyperglycemia in predicting mortality for critically ill hemorrhagic stroke patients: insights from MIMIC-IV

**DOI:** 10.3389/fendo.2025.1558352

**Published:** 2025-05-02

**Authors:** Yong Yue, Pengcheng Li, Zhengyu Sun, Xiaoyi Wang, Zongping Li, Ye Zhang

**Affiliations:** ^1^ Department of Neurosurgery, Mianyang Central Hospital, School of Medicine, University of Electronic Science and Technology of China, Mianyang, Sichuan, China; ^2^ Division of Clinical Neuroscience, Chiba University Center for Forensic Mental Health, Chiba, Japan; ^3^ Department of Pharmacology, Chiba University Graduate School of Medicine, Chiba, Japan; ^4^ The Department of Oncology, The First Affiliated Hospital of the Chengdu Medical College, Chengdu, Sichuan, China; ^5^ Department of Plastic and Aesthetic, Jintang First People’s Hospital, Chengdu, Sichuan, China

**Keywords:** stress hyperglycemia ratio, hemorrhagic stroke, prognosis, MIMIC-IV database, all-cause mortality

## Abstract

**Background:**

Hemorrhagic stroke (HS), including intracerebral hemorrhage (ICH) and subarachnoid hemorrhage (SAH), is associated with high mortality and morbidity. Stress hyperglycemia ratio (SHR), reflecting acute glycemic responses relative to baseline glucose levels, has been linked to poor outcomes in critical illnesses. However, research on its prognostic significance in HS patients admitted to the intensive care unit (ICU) is limited. This study aims to assess the association between SHR and all-cause mortality (ACM) in critically ill HS patients.

**Methods:**

Patients diagnosed with HS were extracted from the Medical Information Mart for Intensive Care-IV (MIMIC-IV) database using ICD-9/10 codes. SHR was calculated as [admission glucose (mg/dL)/(28.7 × HbA1c (%) − 46.7)]. Patients were stratified into tertiles. Primary outcomes were ICU, in-hospital, 30-day, 90-day, 180-day, and 1-year mortality. Cox regression and restricted cubic splines (RCS) evaluated the dose-response relationship between SHR and ACM. Kaplan-Meier (K-M) analysis assessed survival across tertiles, with subgroup analysis and interaction tests for effect modification.

**Results:**

The study included 1,749 patients, with a median age of 68 years (IQR: 57–79), and 53.2% were male. The observed mortality rates were 10.6% in the ICU, 15.2% in-hospital, 19.6% at 30 days, 24.2% at 90 days, 27.8% at 180 days, and 31.7% at 1 year. Multivariate Cox regression analysis indicated that elevated SHR was independently associated with increased ACM at 30 days (adjusted hazard ratio [aHR]: 1.41; 95% confidence interval [CI]: 1.10–1.81; P = 0.006), 90 days (aHR: 1.33; 95% CI: 1.08–1.65; P = 0.008), and 1 year (aHR: 1.27; 95% CI: 1.05–1.54; P = 0.014). RCS analysis demonstrated a linear association between SHR and ACM, with no evidence of non-linearity. Subgroup analysis revealed consistent associations across various patient characteristics.

**Conclusion:**

SHR is significantly associated with ACM in critically ill patients with HS, supporting its potential role as a prognostic marker for risk stratification and guiding clinical management. Incorporating SHR into routine risk assessment may facilitate early identification of high-risk patients, enabling timely interventions and improved outcomes.

## Introduction

Hemorrhagic stroke (HS), comprising intracerebral hemorrhage (ICH) and subarachnoid hemorrhage (SAH), remains a significant global health concern due to its high rates of mortality, disability, and economic burden (1, 2). HS occurs when a blood vessel ruptures, leading to bleeding into the brain tissue or surrounding areas, causing elevated intracranial pressure and subsequent neurological damage. Although HS accounts for 10–20% of all stroke cases, it is responsible for nearly 40% of stroke-related deaths, highlighting its disproportionate contribution to stroke mortality (2, 3). The global incidence of HS in 2019 was 41.81 per 100,000 person-years for ICH and 14.41 per 100,000 person-years for SAH (2). Mortality rates for ICH were higher in low- to upper-middle-income countries compared to high-income countries (29.5% vs. 15.8%). Conversely, SAH mortality was lower in low- to upper-middle-income countries compared to high-income countries (7.9% vs. 19.7%) (2). Despite progress in stroke management, including supportive care, surgical interventions, and blood pressure regulation, long-term outcomes are often suboptimal (4). Patients who survive HS frequently endure lasting neurological and cognitive impairments, resulting in long-term disability and placing substantial strain on healthcare resources and families (5). In general, SAH caused by aneurysm rupture can result in favorable outcomes with prompt intervention, whereas ICH is often linked to more severe neurological impairment and worse prognoses. Recent studies indicated that early implementation of care bundles, incorporating strict blood pressure control, glycemic stabilization, temperature management, and anticoagulation optimization within hours of symptom onset, can lead to significant improvements in functional outcomes (6). Additionally, another study demonstrated that in patients undergoing surgery within 24 hours of symptom onset, minimally invasive hematoma evacuation is associated with superior functional recovery at 180 days when compared to standard medical management (7). Given the rising incidence of HS, particularly in aging populations and ICU settings, there is a pressing need to develop reliable prognostic tools and implement comprehensive treatment approaches to improve survival and mitigate long-term disability.

Stress hyperglycemia, defined as a transient elevation of blood glucose levels in response to acute physiological stress, is a common occurrence in critically ill patients (8, 9). This phenomenon is frequently observed in acute settings such as cerebrovascular events (including stroke, acute myocardial infarction, acute coronary syndrome), post-surgical recovery, and intensive care unit (ICU) admissions (8). While stress hyperglycemia is considered part of the body’s adaptive response to critical illness, mounting evidence suggests that excessive glycemic responses may exacerbate disease progression and worsen clinical outcomes (8). To better assess the clinical significance of stress hyperglycemia, the stress hyperglycemia ratio (SHR) has emerged as a novel marker, calculated by normalizing admission blood glucose to long-term glycemic control, represented by HbA1c (10, 11). Unlike isolated blood glucose measurements, SHR provides a dynamic reflection of acute glycemic disturbances relative to a patient’s baseline glucose levels, offering a more accurate evaluation of glycemic dysregulation during acute illness. This distinction is particularly relevant in critically ill patients, where elevated glucose levels at admission may not necessarily indicate poor glycemic control under normal conditions. SHR has gained increasing recognition as a prognostic marker for adverse outcomes in a range of critical illnesses, including sepsis, cardiovascular disease, and ICH (12–19). Studies have consistently demonstrated that elevated SHR correlates with higher mortality and poorer clinical outcomes across these conditions. In ICH, elevated SHR not only predicts mortality but has also been identified as a significant indicator of hematoma expansion, a key determinant of neurological deterioration and long-term disability (16, 18).

The role of SHR in predicting clinical outcomes in HS, particularly among critically ill patients, remains insufficiently understood. This study aims to explore the potential of SHR as a predictive marker for mortality in critically ill HS patients, a population that faces a significantly higher risk of poor outcomes compared to ischemic stroke (IS) patients. By leveraging data from the comprehensive Medical Information Mart for Intensive Care (MIMIC)-IV database, we seek to clarify the relationship between SHR levels and all-cause mortality (ACM) in HS patients. Our research is designed to address existing gaps in understanding the prognostic value of SHR in HS, contributing to more accurate risk stratification and guiding clinical decision-making. Through this study, we aim to enhance healthcare management by promoting earlier interventions and improving outcomes for patients at elevated risk of mortality and severe neurological complications.

## Methods

This retrospective observational cohort study involved longitudinal follow-up of patients, utilizing data from the Medical Information Mart for Intensive Care-IV (MIMIC-IV 3.1). MIMIC-IV 3.1 is a publicly accessible database encompassing 94, 458 ICU admissions at Beth Israel Deaconess Medical Center in Boston, Massachusetts, covering the period from 2008 to 2022 (20). The database contains extensive clinical information, including patient demographics, vital signs, laboratory test results, and diagnostic data encoded using both the Ninth (ICD-9) and Tenth Revisions (ICD-10) of the International Classification of Diseases. Access to the MIMIC-IV database required certification, which was successfully obtained by one of the authors (Yong Yue), who subsequently conducted the data extraction for this study (Record ID: 669892555). In preparation, the author (Yong Yue) completed specialized training to ensure adherence to standardized protocols and procedural guidelines. To ensure data integrity and reliability, multiple validation measures were applied. This process involved independent cross-checking of critical data points and the use of statistical software to perform consistency checks, allowing for the identification and correction of potential discrepancies or errors during data handling and analysis.

**Figure 1:**
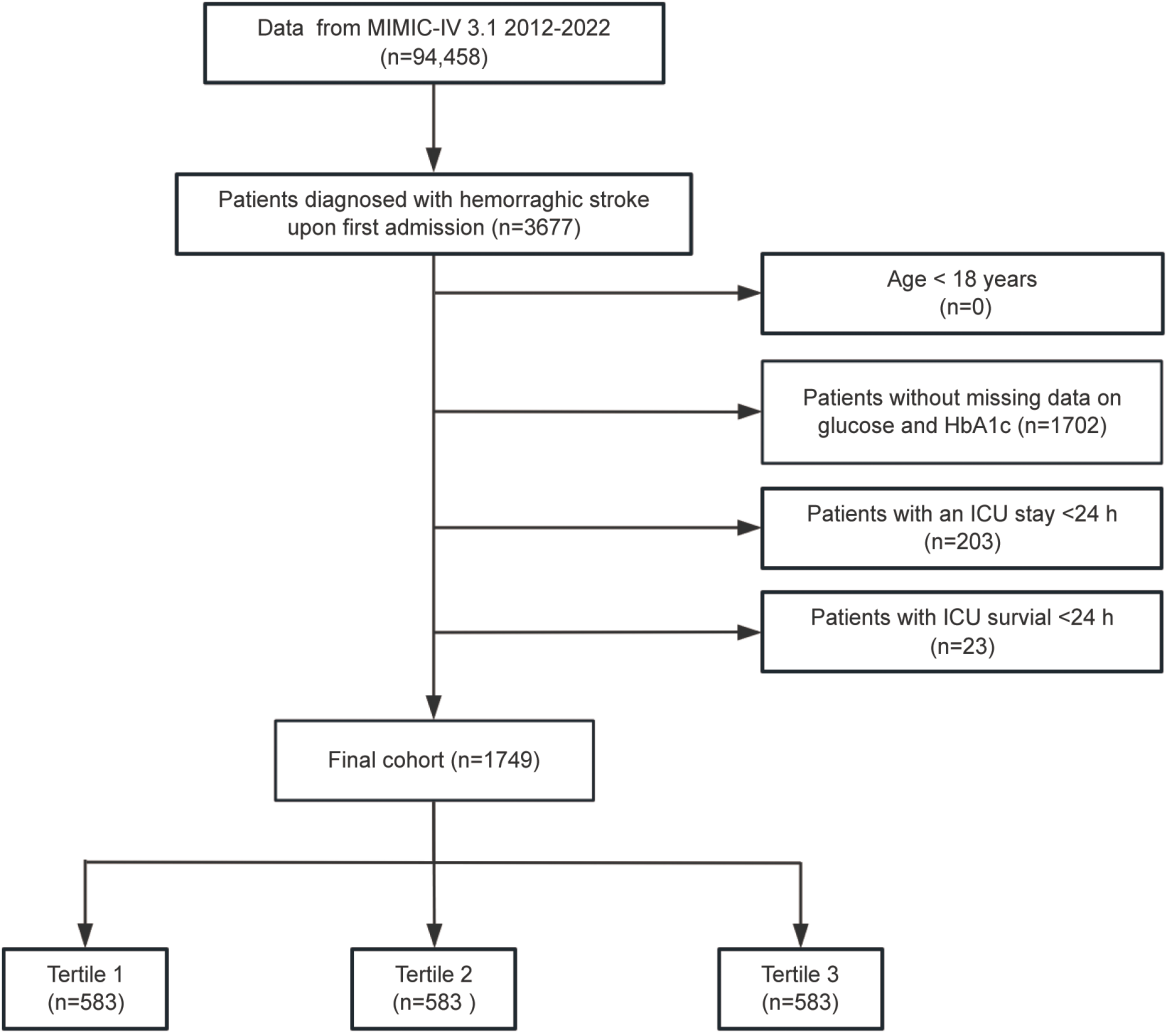
Flowchart illustrating the selection process for patients diagnosed with hemorrhagic stroke (HS) from the MIMIC-IV database.

Patients diagnosed with HS were selected according to the International Classification of Diseases, Ninth and Tenth Revisions (ICD-9/10) criteria. ICH was identified using ICD-9 code 431 and ICD-10 codes I61 and I62.9, while non-traumatic SAH was defined by ICD-9 code 430 and ICD-10 code I60.To ensure data integrity and relevance, the following exclusion criteria were applied: (1) patients under the age of 18; (2) absence of fasting blood glucose (FBG) or HbA1c measurements on the first day of ICU admission; (3) ICU stays of less than 24 hours; and (4) patients with multiple ICU admissions for HS, with only data from the initial admission being retained. After implementing these criteria, 1,749 patients were identified and divided into three groups according to tertiles of SHR for further analysis (**Figure 1**).

### Data extraction

Data extraction was conducted using Navicat Premium (Version 16.1.15) with structured query language (SQL) to retrieve comprehensive clinical information at the time of ICU admission. Extracted data encompassed demographic details, including age, gender, and ethnicity, as well as clinical severity scores such as the Glasgow Coma Scale (GCS), Sequential Organ Failure Assessment (SOFA), Simplified Acute Physiology Score-II (SAPS-II), Acute Physiology Score-III (APS-III), Oxford Acute Severity of Illness Score (OASIS), and Systemic Inflammatory Response Syndrome Score (SIRS).Baseline vital parameters included measurements of mean blood pressure (MBP), systolic blood pressure (SBP), diastolic blood pressure (DBP), heart rate (HR), respiratory rate (RR), oxygen saturation (SpO_2_), and temperature. Laboratory data comprised red blood cell count (RBC), hemoglobin (Hb), platelet count (PLT), white blood cell count (WBC), sodium (Na), potassium (K), blood urea nitrogen (BUN), creatinine (Cr), fasting blood glucose (FBG), anion gap (AG), prothrombin time (PT), activated partial thromboplastin time (APTT), international normalized ratio (INR), and glycated hemoglobin (HbA1c).Information on treatment strategies was also collected, including the use of mechanical ventilation (MV), vasopressors, oxygen delivery, and surgical procedures. Comorbid conditions such as hypertension, diabetes mellitus (DM), heart failure (HF), atrial fibrillation (AF), acute myocardial infarction (AMI), peripheral vascular disease(PVD), chronic obstructive pulmonary disease (COPD), acute kidney injury (AKI), hyperlipidemia, malignancy, renal failure (RF), sepsis, liver disease, ventilator-associated pneumonia (VAP), and Charlson Comorbidity Index (CCI) were recorded.

The observation period for each subject began at the time of admission and continued until mortality occurred. The stress hyperglycemia ratio (SHR) is an index that quantifies the extent of hyperglycemia relative to baseline glycemic status. It is determined using the formula: SHR= (admission blood glucose (mg/dl))/(28.7 × HbA1c (%) − 46.7). The analysis was conducted using laboratory measurements and disease severity scores collected within the first 24 hours of ICU admission, providing an early assessment of the patient’s clinical status. No individual variable exhibited more than 20% missing data. Any missing values were addressed using multiple imputation methods to enhance data completeness and ensure analytical robustness.

### Clinical outcomes

The study aimed to evaluate ACM outcomes in HS patients from the MIMIC-IV database by analyzing mortality at various time points following ICU admission. The primary outcomes included ICU and in-hospital mortality, as well as ACM at 30, 90, 180 days, and 1year post-admission, representing short-term, intermediate, and long-term follow-up periods. Secondary outcomes focused on ICU and hospital length of stay (LOS).

### Feature selection

**Figure 2:**
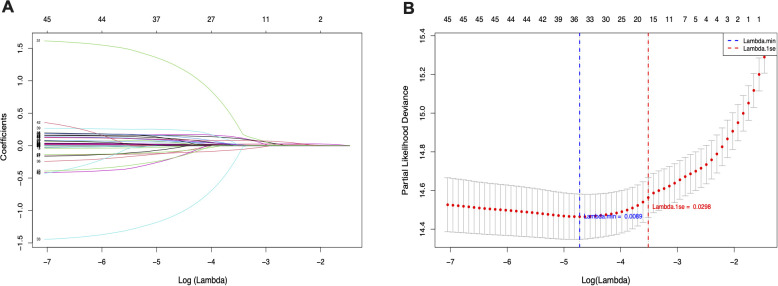
LASSO penalized regression analysis for identifying key variables associated with all-cause mortality in patients with hemorrhagic stroke. **(A)** Coefficient profiles of variables plotted against the log(lambda) sequence. **(B)** Ten-fold cross-validation for tuning parameter selection in the LASSO model. The vertical dashed line indicates the optimal lambda value that minimizes the partial likelihood deviance.

Before examining the association between SHR and ACM in patients with HS, we applied Least Absolute Shrinkage and Selection Operator (LASSO) regression for feature selection. This approach not only reduces model complexity but also minimizes the risk of overfitting by penalizing less informative variables. A ten-fold cross-validation procedure was utilized to determine the optimal penalty term λ (lambda), ensuring the selection of variables that provide the best balance between model accuracy and simplicity. As a result, eight key predictors were identified, including MV, oxygen delivery, AMI, PVD, VAP, INR, SIRS, and malignancy, as demonstrated in **Figure 2**. These variables were found to be the most influential in predicting all-cause mortality among patients with hemorrhagic stroke.

### Statistical analysis

An initial evaluation was conducted to assess the distribution patterns of continuous variables. Based on their SHR values, participants were stratified into tertiles (T1–T3) for further analysis. Continuous data were summarized as either mean ± standard deviation (SD) or median with interquartile range (IQR), depending on whether the data followed a normal or skewed distribution. Categorical variables were expressed as counts and percentages. For comparisons involving normally distributed continuous variables, the t-test or analysis of variance (ANOVA) was applied. When the data deviated from normality, non-parametric tests, including the Mann-Whitney U test and Kruskal-Wallis test, were utilized. Categorical variables were analyzed across SHR quartiles using Pearson’s chi-squared test to detect significant differences among groups.

Kaplan-Meier (K-M) survival analysis was performed to estimate endpoint incidence across different SHR groups, with the log-rank test used to compare survival curves and assess statistical significance between strata. Cox proportional hazards models were applied to evaluate the relationship between SHR and study outcomes, producing HRs and 95% CIs. Three models were developed to account for potential confounders. Model 1 served as the unadjusted baseline. Model 2 incorporated adjustments for fundamental demographic factors, including age, sex, and race. Model 3 expanded upon this by integrating a comprehensive set of variables identified through a combination of clinical expertise, established literature, and feature selection using LASSO regression. The variables in Model 3 included age, sex, race, sodium, hemoglobin, WBC, surgical procedure, GCS, CR, MV, oxygen therapy, AMI, PVD, VAP, INR, SIRS, malignancy, hypertension, and DM. SHR was analyzed both as a continuous variable and by tertiles, with the lowest quartile serving as the reference. To assess the potential nonlinear relationship between SHR and ACM, restricted cubic spline (RCS) analysis was employed to evaluate the dose-response association across short-, intermediate-, and long-term outcomes. Additionally, the proportional hazards (PH) assumption of the Cox models was evaluated based on Model 3 using Schoenfeld residuals and global statistical tests. Moreover, subgroup analyses were conducted to examine the modifying effects of age (<65 or ≥65 years), gender, race, GCS (13-15 or 3-12), hypertension, DM, and surgical procedures on the association between SHR and ACM. Cox proportional hazards models were used to calculate hazard ratios (HRs) and 95% confidence intervals (CIs), with interaction terms evaluated through likelihood ratio tests to assess statistical significance across subgroups. Each subgroup analysis was adjusted for the same covariates as Model 3, with the exception of the subgroup variable itself. To evaluate the robustness of the observed associations to potential unmeasured confounding, we calculated E-values for the hazard ratios (HRs) of SHR and all-cause mortality outcomes. The E-value quantifies the minimum strength of association that an unmeasured confounder would need to have with both the exposure (SHR) and the outcome, conditional on the measured covariates, to fully explain away the observed association. Higher E-values indicate that stronger unmeasured confounding would be required to negate the findings. All statistical analyses were conducted using R software (version 4.3.3). A two-tailed P value of less than 0.05 was considered statistically significant.

## Results

A total of 1,749 patients diagnosed with HS were included in the study, with a median age of 68 years (IQR: 57–79). Males comprised 53.2% (n = 930) of the cohort. The median SHR across the population was 1.07 (IQR: 0.91–1.28). Mortality rates during the study period were recorded at 10.6% in the ICU and 15.2% during hospitalization, with mortality progressively increasing to 19.6%, 24.2%, 27.8%, and 31.7% at 30 days, 90 days, 180 days, and 1 year, respectively.

### Baseline characteristics

**Table 1** outlines the baseline demographic and clinical characteristics of the cohort, stratified by SHR tertiles. Patients were categorized into three tertiles based on their SHR values at hospital admission: Tertile 1 (T1: 0.18–0.96), Tertile 2 (T2: 0.96–1.19), and Tertile 3 (T3: 1.19–4.16). The median SHR values for T1, T2, and T3 were 0.85 (IQR: 0.77–0.91), 1.07 (IQR: 1.01–1.13), and 1.38 (IQR: 1.28–1.59), respectively.

**Table 1:** The baseline characteristics and outcomes of participants classified by SHR tertiles.

Variable	Overall (n=1749)	Tertile 1 (n=583)	Tertile 2 (n=583)	Tertile 3 (n=583)	*P* value
**SHR**	1.07 (0.91,1.28)	0.85 (0.77,0.91)	1.07 (1.01,1.13)	1.38 (1.28,1.59)	<0.001
**Demographics**					
**Age, years**	68.0 (57.0,79.0)	69.0 (59.0,81.0)	68.0 (56.0,78.0)	67.0 (55.5;77.0)	0.002
**Gender, n (%)**					0.648
Male	930 (53.2%)	313 (53.7%)	316 (54.2%)	301 (51.6%)	
Female	819 (46.8%)	270 (46.3%)	267 (45.8%)	282 (48.4%)	
**Race, n (%)**					0.003
Asian	76 (4.35%)	18 (3.09%)	26 (4.46%)	32 (5.49%)	
White	183 (10.5%)	73 (12.5%)	62 (10.6%)	48 (8.23%)	
Black	964 (55.1%)	329 (56.4%)	338 (58.0%)	297 (50.9%)	
Others	526 (30.1%)	163 (28.0%)	157 (26.9%)	206 (35.3%)	
**Comorbidities**					
**Hypertension, n (%)**	1130 (64.6%)	369 (63.3%)	370 (63.5%)	391 (67.1%)	0.314
**Diabetes mellitus, n (%)**	559 (32.0%)	201 (34.5%)	149 (25.6%)	209 (35.8%)	<0.001
**Heart failure, n (%)**	240 (13.7%)	87 (14.9%)	77 (13.2%)	76 (13.0%)	0.585
**atrial fibrillation, n (%)**	472 (27.0%)	168 (28.8%)	162 (27.8%)	142 (24.4%)	0.199
**Acute myocardial infarction, n (%)**	13 (0.74%)	3 (0.51%)	4 (0.69%)	6 (1.03%)	0.693
**Peripheral vascular disease, n (%)**	27 (1.54%)	14 (2.40%)	6 (1.03%)	7 (1.20%)	0.117
**Chronic obstructive pulmonary disease, n (%)**	71 (4.06%)	28 (4.80%)	24 (4.12%)	19 (3.26%)	0.408
**Acute kidney injury, n (%)**	1096 (62.7%)	329 (56.4%)	364 (62.4%)	403 (69.1%)	<0.001
**Hyperlipidemia, n (%)**	758 (43.3%)	291 (49.9%)	242 (41.5%)	225 (38.6%)	<0.001
**Malignancy, n (%) **	295 (16.9%)	90 (15.4%)	102 (17.5%)	103 (17.7%)	0.527
**Renal failure, n (%)**	1153 (65.9%)	359 (61.6%)	379 (65.0%)	415 (71.2%)	0.002
**Sepsis, n (%)**	685 (39.2%)	186 (31.9%)	222 (38.1%)	277 (47.5%)	<0.001
**Ventilator-associated pneumonia, n (%)**	82 (4.69%)	25 (4.29%)	27 (4.63%)	30 (5.15%)	0.784
**Liver disease, n (%)**	170 (9.72%)	42 (7.20%)	47 (8.06%)	81 (13.9%)	<0.001
**Charlson comorbidity index**	6.00 (5.00, 8.00)	7.00 (5.00, 8.00)	6.00 (5.00, 8.00)	7.00 (5.00, 8.00)	0.016
**vital signs**					
**Mean blood pressure, mmHg**	92.0 (81.0, 103)	93.0 (82.0, 103)	93.0 (82.0, 103)	92.0 (80.0, 102)	0.285
**Systolic blood pressure, mmHg**	137 (122, 150)	137 (123, 150)	137 (122, 150)	136 (120, 149)	0.337
**Diastolic blood pressure, mmHg**	75.0 (64.0, 87.0)	75.0 (66.5, 87.0)	75.0 (64.0, 87.0)	75.0 (63.0, 85.0)	0.157
**Mean heart rate, beats/min**	79.4 (70.5, 89.0)	76.1 (68.8, 85.2)	79.3 (69.7, 88.8)	82.9 (73.7, 93.4)	<0.001
**Respiratory rate, times/min**	18.0 (15.5, 21.0)	18.0 (15.0, 21.0)	18.0 (15.0, 21.0)	18.0 (16.0, 22.0)	0.017
**Temperature, °C**	36.8 (36.6, 37.1)	36.8 (36.6, 37.1)	36.9 (36.6, 37.1)	36.8 (36.5, 37.1)	0.149
**Saturation of pulse oxygen, %**	98.0 (96.0, 100)	98.0 (95.0, 99.0)	98.0 (96.0, 100)	98.0 (96.0, 100)	0.022
**Laboratory parameters**					
**Red blood cell, 109/L**	4.13 (3.70, 4.55)	4.18 (3.73, 4.62)	4.12 (3.74, 4.52)	4.08 (3.58, 4.51)	0.009
**Hemoglobin, g/L**	12.4 (11.1, 13.7)	12.5 (11.0, 13.7)	12.5 (11.3, 13.7)	12.3 (10.9, 13.6)	0.321
**Platelet, 109/L**	208 (166, 264)	209 (168, 261)	209 (166, 266)	206 (162, 262)	0.685
**White blood cell, 109/L**	10.2 (7.90, 13.2)	8.90 (7.10, 11.4)	10.2 (8.10, 12.8)	11.7 (9.30, 14.6)	<0.001
**Sodium, mmol/L**	139 (137, 142)	140 (138, 142)	139 (137, 142)	139 (136, 141)	<0.001
**Potassium, mmol/L**	3.90 (3.60, 4.30)	3.90 (3.60, 4.30)	3.90 (3.60, 4.30)	3.90 (3.60, 4.30)	0.994
**Blood urea nitrogen, mg/dL**	16.0 (12.0, 21.0)	15.0 (12.0, 21.0)	15.0 (12.0, 21.0)	16.0 (12.0, 22.0)	0.049
**Creatinine, mg/24 h**	0.90 (0.70, 1.10)	0.90 (0.70, 1.10)	0.90 (0.70, 1.10)	0.90 (0.70, 1.20)	0.037
**Fasting blood glucose, mg/dL**	128 (107, 159)	102 (93.0, 116)	125 (113, 140)	166 (144, 216)	<0.001
**HBA1C，%**	5.70 (5.40, 6.30)	5.90 (5.50, 6.40)	5.70 (5.35, 6.10)	5.70 (5.30, 6.50)	<0.001
**AG, mmol/L**	14.0 (12.0, 16.0)	14.0 (12.0, 16.0)	14.0 (12.0, 16.0)	15.0 (13.0, 17.0)	<0.001
**Prothrombin time, s**	12.6 (11.7, 13.8)	12.5 (11.7, 13.8)	12.6 (11.7, 13.7)	12.6 (11.7, 14.0)	0.704
**Activated partial thromboplastin time, s**	28.3 (25.9, 31.4)	28.5 (26.2, 31.6)	28.4 (26.0, 31.4)	28.0 (25.6, 31.2)	0.178
**International normalized ratio**	1.10 (1.10, 1.30)	1.10 (1.10, 1.25)	1.12 (1.10, 1.20)	1.10 (1.10, 1.30)	0.896
**Clinical severity scores**					
**Baseline Glasgow Coma score**	15.0 (14.0, 15.0)	15.0 (14.0, 15.0)	15.0 (14.0, 15.0)	15.0 (14.0, 15.0)	0.922
**SOFA**	0.00 (0.00, 2.00)	0.00 (0.00, 1.00)	0.00 (0.00, 1.00)	1.00 (0.00, 2.00)	0.001
**SAPS-II**	32.0 (24.0, 39.0)	31.0 (24.0, 39.0)	31.0 (24.0, 37.5)	34.0 (26.0, 42.0)	<0.001
**SIRS**	2.00 (2.00, 3.00)	2.00 (1.00, 3.00)	2.00 (2.00, 3.00)	3.00 (2.00, 3.00)	<0.001
**OASIS**	32.0 (26.0, 38.0)	31.0 (25.0, 37.0)	32.0 (26.0, 37.0)	35.0 (28.0, 40.0)	<0.001
**APS-III**	39.0 (28.0, 52.0)	35.0 (26.0, 48.5)	37.0 (27.0, 49.0)	46.0 (32.0, 61.0)	<0.001
**Treatment**					
**Mechanical ventilation, n (%)**	1265 (72.3%)	361 (61.9%)	423 (72.6%)	481 (82.5%)	<0.001
**Vasopressors, n (%)**	254 (14.5%)	66 (11.3%)	67 (11.5%)	121 (20.8%)	<0.001
**Oxygen delivery, n (%)**	1274 (72.8%)	379 (65.0%)	435 (74.6%)	460 (78.9%)	<0.001
**Surgical procedure, n(%)**	394 (22.5%)	114 (19.6%)	129 (22.1%)	151 (25.9%)	0.033
**Clinical outcomes**					
**LOS ICU, day**	4.56 (2.39, 8.98)	3.67 (1.97, 7.38)	4.66 (2.65, 8.66)	5.72 (2.76, 10.2)	<0.001
**LOS hospital, day**	9.58 (5.00, 17.0)	8.04 (4.25, 15.0)	9.25 (5.52, 16.90)	11.3 (5.96, 18.5)	<0.001
**All-cause mortality**					
**ICU mortality, n (%)**	185 (10.6%)	34 (5.83%)	51 (8.75%)	100 (17.2%)	<0.001
**In-hospital mortality, n (%)**	266 (15.2%)	52 (8.92%)	79 (13.6%)	135 (23.2%)	<0.001
**30-day mortality, n (%)**	342 (19.6%)	78 (13.4%)	99 (17.0%)	165 (28.3%)	<0.001
**90-day mortality, n (%)**	423 (24.2%)	104 (17.8%)	124 (21.3%)	195 (33.4%)	<0.001
**180-day mortality, n (%)**	487 (27.8%)	126 (21.6%)	146 (25.0%)	215 (36.9%)	<0.001
**1-year mortality, n (%)**	554 (31.7%)	150 (25.7%)	169 (29.0%)	235 (40.3%)	<0.001

Continuous variables are expressed as mean ± standard deviation (SD) or median [interquartile range, IQR], depending on data distribution. Categorical variables are presented as number (percentage). For comparisons, t-tests or ANOVA were used for normally distributed continuous variables, and Mann–Whitney U or Kruskal–Wallis tests for non-normally distributed variables. Pearson’s chi-square test was applied to compare categorical variables across SHR quartiles.

SHR: T1:0.18-0.96; T2:0.96-1.19; T3:1.19-4.16. SHR: stress hyperglycemia; SOFA, sequential organ failure assessment score; SAPS-II, simplified acute physiology score; SIRS, systemic inflammatory response syndrome score; OASIS, oxford acute severity of illness score; APS-III, acute physiology score-III; LOS, length of stay; ICU, intensive care unit.

Patients in the highest tertile (T3) demonstrated significantly higher rates of comorbid conditions and complications, including diabetes mellitus (35.8%), acute kidney injury (69.1%), sepsis (47.5%), liver disease (13.9%), and renal failure (71.2%) compared to those in the lower tertiles (P < 0.05). Notably, T3 patients had elevated severity scores, with SOFA of 1.00 (IQR: 0.00–2.00), SAPS-II of 34 (IQR: 26–42), SIRS of 3.00 (IQR: 2.00–3.00), OASIS of 35.0 (IQR: 28.0–40.0), and APS-III of 46 (IQR: 32–61). T3 patients required more intensive medical interventions compared to their counterparts in T1 and T2, with higher rates of mechanical ventilation (82.5%), vasopressor support (20.8%), oxygen therapy (78.9%), and surgical interventions (25.9%) (P < 0.05).

Laboratory analyses revealed that T3 patients exhibited significantly higher levels of systemic inflammation and metabolic dysregulation. WBC counts were elevated at 11.7 × 10^9^/L (IQR: 9.30–14.6), along with BUN at 16.0 (IQR: 12.0–22.0), creatinine at 0.90 mg/dL (IQR: 0.70–1.20), and fasting blood glucose at 166 mg/dL (IQR: 144–216). Anion gap values, indicative of metabolic acidosis, were higher in T3 (15 mmol/L, IQR: 13–17), underscoring the severity of metabolic disturbances in this group.

Interestingly, while most clinical and laboratory indicators deteriorated in T3, certain parameters showed unexpected reductions. T3 patients were younger (median age: 67 years, IQR: 55.5–77.0) compared to T1 (69 years, IQR: 59.0–81.0) and T2 (68 years, IQR: 56.0–78.0). Hyperlipidemia was less common in T3 (38.6%) than in T1 (49.9%) and T2 (41.5%). Additionally, RBC counts (4.08, IQR: 3.58–4.51), sodium (139 mmol/L, IQR: 136–141), and HbA1c (5.7%, IQR: 5.3–6.5) were all lower in T3 (P < 0.05).

ICU and hospital stays were significantly longer in T3, reflecting increased disease severity. T3 patients had a median ICU stay of 5.72 days (IQR: 2.76–10.2), compared to 3.67 days (IQR:1.97–7.38) in T1 and 4.66 days (IQR: 2.65–8.66) in T2 (P < 0.001). Total hospitalization duration followed a similar trend, with T3 patients staying a median of 11.3 days (IQR: 5.96–18.5), versus 8.04 days (IQR: 4.25–15.0) in T1 and 9.25 days (IQR: 5.52–16.9) in T2. ICU mortality was markedly higher in T3 at 17.2%, compared to 5.83% in T1 and 8.75% in T2. Similarly, in-hospital mortality rates were highest in T3 (23.2%) relative to T1 (8.92%) and T2 (13.6%) (P < 0.001). Mortality rates at 30 days (28.3%), 90 days (33.4%), 180 days (36.9%), and 1 year (40.3%) followed a similar gradient across tertiles, with T3 patients consistently demonstrating the poorest outcomes (P < 0.001).

### Clinical outcomes

**Figure 3:**
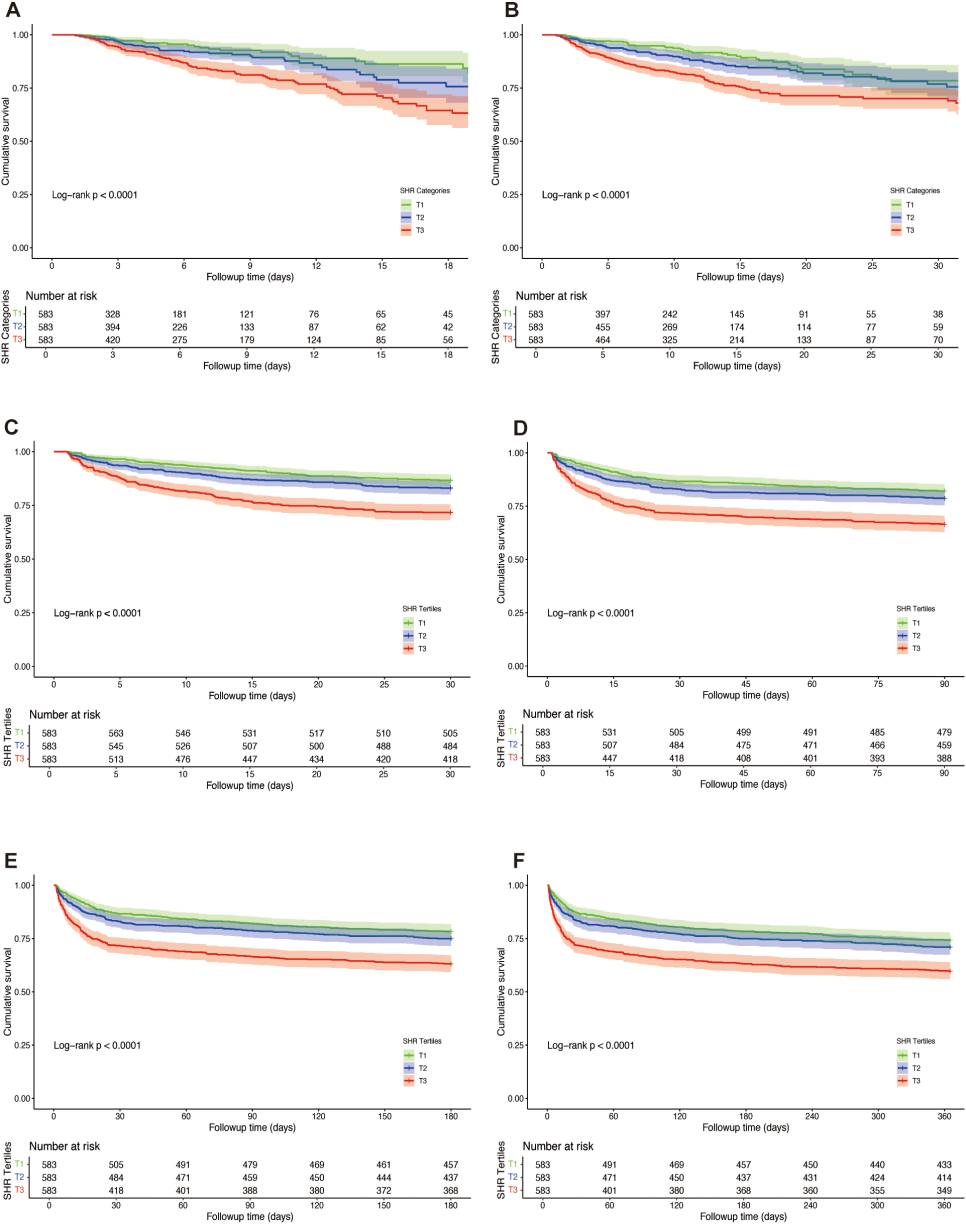
Kaplan-Meier survival curves for all-cause mortality (ACM) across tertiles of stress hyperglycemia ratio (SHR) in patients with hemorrhagic stroke. Survival probabilities are depicted for ICU mortality **(A)**, in-hospital mortality **(B)**, 30-day mortality **(C)**, 90-day mortality **(D)**, 180-day mortality **(E)**, and 1-year mortality **(F)**. Log-rank tests were used to compare survival differences across tertiles.

K-M survival analysis was employed to assess ACM across SHR tertiles, as depicted in **Figure 3**. The findings revealed that patients in the highest tertile (T3) exhibited significantly elevated mortality at every assessed interval compared to those in the lower tertiles. This trend remained consistent across ICU mortality, in-hospital mortality, as well as 30-day, 90-day, 180-day, and 1-year mortality (log-rank p < 0.0001 for each endpoint).

**Figure 4:**
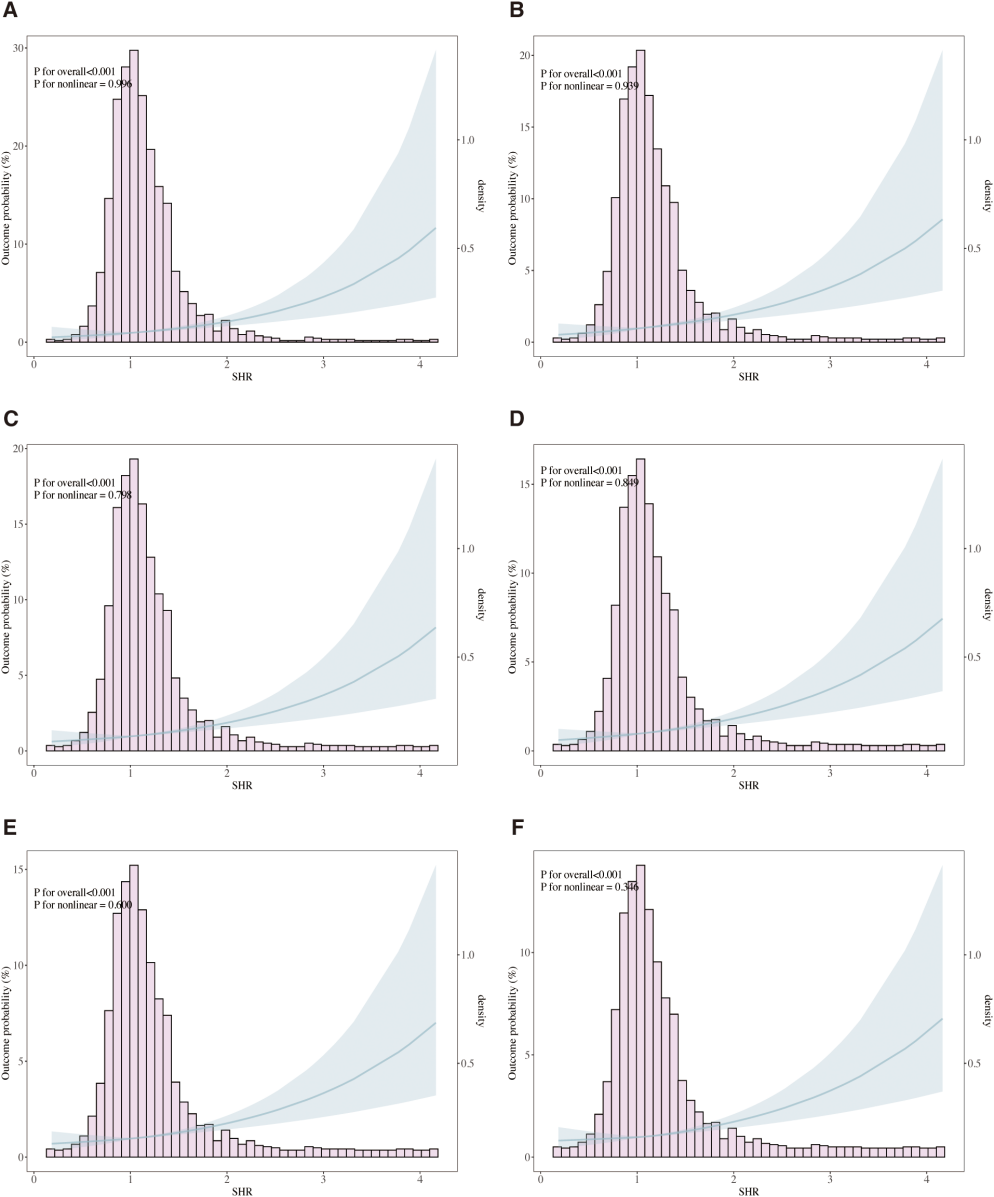
Restricted cubic spline (RCS) curves illustrating the association between stress hyperglycemia ratio (SHR) and all-cause mortality (ACM). The analysis models ICU mortality **(A)**, in-hospital mortality **(B)**, 30-day mortality **(C)**, 90-day mortality **(D)**, 180-day mortality **(E)**, and 1-year mortality **(F)**. Solid lines represent hazard ratios (HRs), and shaded areas denote 95% confidence intervals. Adjusted for age, gender, race, sodium, hemoglobin, WBC, surgical procedure, GCS, CR, ventilation, oxygen delivery, AMI, peripheral vascular disease, ventilator-associated pneumonia, INR, SIRS, malignancy, hypertension and diabetes.

The relationship between SHR and ACM at various time points (ICU, in-hospital, 30 days, 90 days, 180 days, and 1 year) was evaluated through Cox proportional hazards modeling. SHR emerged as a significant predictor of ICU mortality across all analytical models. In the unadjusted model (Model 1), elevated SHR levels were associated with a 3.23-fold higher risk of ICU mortality (95% CI: 2.67–3.97, p < 0.001). This association remained robust in the partially adjusted model (Model 2: HR 3.11, 95% CI: 2.52–3.83, p < 0.001) and persisted in the fully adjusted model (Model 3: HR 2.21, 95% CI: 1.70–2.88, p = 0.002). **Table 2** presents a comprehensive overview of the associations between SHR and ACM at ICU, in-hospital, 30 days, 90 days, and 1 year. When stratified by tertiles, patients in the highest tertile (T3) demonstrated the greatest risk of ICU mortality, with a 3.23-fold increase in Model 1 (95% CI: 2.19–4.76, p < 0.001) and a 1.88-fold increase in Model 3 (95% CI: 1.25–2.83, p = 0.002) compared to those in the lowest tertile (T1). A consistent upward trend in ICU mortality was observed across tertiles (p for trend < 0.001), indicating a dose-response relationship between SHR and adverse outcomes. Similar trends were observed in multivariate Cox regression analyses assessing in-hospital, 30-day, 90-day, 180-day, and 1-year mortality. Additionally, RCS regression modeling demonstrated a linear relationship between SHR and the risk of ICU, in-hospital, 30-day, 90-day, 180-day, and 1-year mortality, with no significant nonlinearity observed at any interval (P for nonlinearity = 0.996, 0.939, 0.798, 0.849, 0.600, and 0.346, respectively), as shown in **Figure 4**. The proportional hazards (PH) assumption was assessed for each Cox model using Schoenfeld residuals. The global tests indicated that the PH assumption was not violated for ICU mortality (p = 0.99), in-hospital mortality (p = 0.619), 30-day mortality (p = 0.136), and 90-day mortality (p = 0.169). However, minor violations were observed for 180-day (p = 0.017) and 1-year mortality (p = 0.003).

**Table 2:** Cox regression analysis of the association between SHR and all-cause mortality.

All-cause mortality	Model 1		Model 2		Model 3	
	HR (95% CI)	*P* value	HR (95% CI)	*P* value	HR (95% CI)	*P* value
ICU mortality						
SHR (overall)	3.23 (2.63, 3.97)	<0.001	3.11 (2.52, 3.83)	<0.001	2.21 (1.7, 2.88)	<0.001
T1	Reference		Reference		Reference	
T2	1.54 (1.00, 2.38)	0.051	1.60 (1.04, 2.48)	0.034	1.48 (0.95, 2.31)	0.081
T3	3.23 (2.19, 4.76)	<0.001	3.13 (2.12, 4.64)	<0.001	1.88 (1.25, 2.83)	0.002
P for trend	<0.001		<0.001		0.002	
In-hospital mortality						
SHR (overall)	2.89 (2.39, 3.49)	<0.001	2.88 (2.38, 3.49)	<0.001	2.01 (1.59, 2.55)	<0.001
T1	Reference		Reference		Reference	
T2	1.56 (1.10, 2.22)	0.012	1.65 (1.16, 2.35)	0.005	1.57 (1.10, 2.24)	0.014
T3	2.89 (2.1, 3.98)	<0.001	2.91 (2.11, 4.02)	<0.001	1.91 (1.36, 2.66)	<0.001
P for trend	<0.001		<0.001		<0.001	
30-day mortality						
SHR (overall)	2.53 (2.10, 3.05)	<0.001	2.64 (2.18, 3.19)	<0.001	1.93 (1.53, 2.43)	<0.001
T1	Reference		Reference		Reference	
T2	1.31 (0.97, 1.76)	0.076	1.42 (1.05, 1.91)	0.021	1.36 (1.01, 1.85)	0.045
T3	2.37 (1.81, 3.10)	<0.001	2.5 (1.90, 3.28)	<0.001	1.75 (1.32, 2.32)	<0.001
P for trend	<0.001		<0.001		<0.001	
90-day mortality						
SHR (overall)	2.45 (2.06, 2.92)	<0.001	2.63 (2.21, 3.14)	<0.001	1.88 (1.53, 2.32)	<0.001
T1	Reference		Reference		Reference	
T2	1.23 (0.95, 1.60)	0.116	1.35 (1.04, 1.76)	0.024	1.27 (0.97, 1.66)	0.078
T3	2.13 (1.68, 2.71)	<0.001	2.32 (1.82, 2.95)	<0.001	1.66 (1.29, 2.13)	<0.001
P for trend	<0.001		<0.001		<0.001	
180-day mortality						
SHR (overall)	2.27 (1.91, 2.69)	<0.001	2.47 (2.07, 2.94)	<0.001	1.80 (1.47, 2.21)	<0.001
T1	Reference		Reference		Reference	
T2	1.20 (0.94, 1.52)	0.136	1.32 (1.04, 1.68)	0.023	1.24 (0.98, 1.59)	0.079
T3	1.96 (1.58, 2.45)	<0.001	2.16 (1.73, 2.69)	<0.001	1.61 (1.28, 2.03)	<0.001
P for trend	<0.001		<0.001		<0.001	
1-year mortality						
SHR (overall)	2.13 (1.8, 2.53)	<0.001	2.35 (1.98, 2.79)	<0.001	1.73 (1.42, 2.1)	<0.001
T1	Reference		Reference		Reference	
T2	1.17 (0.94, 1.45)	0.169	1.29 (1.03, 1.61)	0.025	1.22 (0.97, 1.52)	0.088
T3	1.82 (1.48, 2.23)	<0.001	2.02 (1.64, 2.49)	<0.001	1.54 (1.24, 1.91)	<0.001
P for trend	<0.001		<0.001		<0.001	

Model 1: Non-adjusted.

Model 2: Adjusted for age, gender, race.

Model 3: Adjusted for age, gender, race, sodium, hemoglobin, WBC, surgical procedure, GCS, CR, ventilation, oxygen delivery, AMI, peripheral vascular disease, ventilator-associated pneumonia, INR, SIRS, malignancy, hypertension and diabetes.

### Subgroup and sensitivity analyses

**Figure 5:**
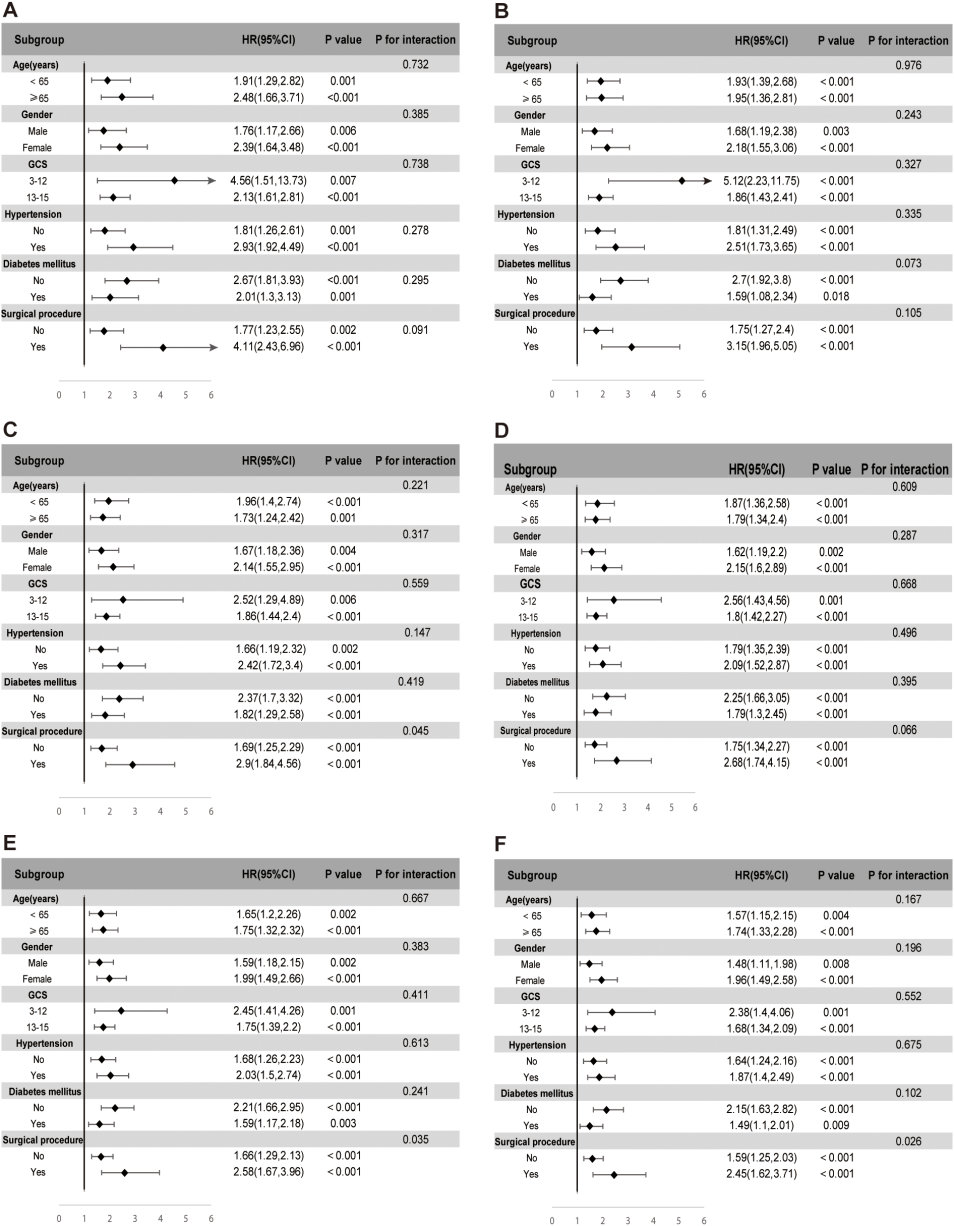
Subgroup analyses evaluating the association between stress hyperglycemia ratio (SHR) and ICU mortality **(A)**, in-hospital mortality **(B)**, 30-day mortality **(C)**, 90-day mortality **(D)**, 180-day mortality **(E)**, and 1-year mortality **(F)**. Each subgroup analysis was adjusted for age, gender, race, sodium, hemoglobin, WBC, surgical procedure, GCS, creatinine, ventilation, oxygen delivery, AMI, peripheral vascular disease, ventilator-associated pneumonia, INR, SIRS, malignancy, hypertension, and diabetes, excluding the subgroup variable itself.

The prognostic value of SHR for predicting ACM was evaluated across various subgroups, including age, gender, GCS scores, hypertension, diabetes mellitus, and surgical procedure. SHR consistently emerged as a significant predictor of mortality at multiple time points, encompassing ICU, in-hospital, 30-day, 90-day, 180-day, and 1-year outcomes, as shown in **Figure 5**.

In the subgroup analysis for ICU mortality, elevated SHR levels were robustly associated with increased risk across age categories. Patients aged ≥65 demonstrated a hazard ratio (HR) of 2.48 (95% CI: 1.66–3.71, p < 0.001), while those under 65 exhibited an HR of 1.91 (95% CI: 1.29–2.82, p = 0.001). Gender-based variations further highlighted this association, with women presenting a higher mortality risk (HR 2.39, 95% CI: 1.64–3.48, p < 0.001) compared to men (HR 1.76, 95% CI: 1.17–2.66, p = 0.006). SHR also retained its predictive value among patients stratified by neurological status, reflected by GCS scores of 3–12 (HR 4.56, 95% CI: 1.51–13.73, p = 0.007) and 13–15 (HR 2.13, 95% CI: 1.61–2.81, p < 0.001). Hypertension significantly amplified the mortality risk associated with SHR, with hypertensive patients demonstrating an HR of 2.93 (95% CI: 1.92–4.49, p < 0.001) compared to an HR of 1.81 (95% CI: 1.26–2.61, p = 0.001) in non-hypertensive individuals. The most pronounced risk, however, was observed in patients undergoing surgical interventions, where SHR conferred an HR of 4.11 (95% CI: 2.43–6.96, p < 0.001), far exceeding the risk observed in non-surgical patients (HR 1.77, 95% CI: 1.23–2.55, p = 0.002). This trend persisted across all subsequent mortality endpoints, including in-hospital, 30-day, 90-day, 180-day, and 1-year follow-up, underscoring the consistent prognostic strength of SHR across different phases of care. While no significant interaction effects were noted within the majority of subgroups, notable interactions emerged in the surgical intervention cohort at the 30-day, 180-day, and 1-year time points.

E-value analysis was conducted to assess the potential influence of unmeasured confounders on the association between SHR and mortality outcomes. As shown in **Table 3**, the calculated E-values for the observed hazard ratios ranged from 2.45 to 3.23, while the E-values for the lower bounds of the 95% confidence intervals ranged from 1.79 to 2.06. Specifically, the E-value for ICU mortality was 3.17 (lower bound: 1.81), and for in-hospital mortality was 3.23 (lower bound: 2.06). For long-term outcomes, including 1-year mortality, the E-value was 2.45 (lower bound: 1.79).

**Table 3:** E-values for the association between SHR and ACM in patients with HS.

	E-Value	E-Value for Lower limit of 95%CI
ICU Mortality	3.17	1.81
In-hospital mortality	3.23	2.06
30-day mortality	2.90	1.97
90-day mortality	2.71	1.90
180-day mortality	2.60	1.87
1-year mortality	2.45	1.79

## Discussion

This study highlights the significant prognostic value of SHR in predicting ACM across multiple time points in patients with HS. Our findings demonstrate that elevated SHR levels are consistently associated with increased mortality during ICU admission, hospitalization, and follow-up periods extending to 30 days, 90 days, 180 days, and 1 year. These results underscore the critical role of metabolic dysregulation in the progression and severity of HS, reinforcing SHR as a valuable biomarker for risk stratification in this patient population.

SHR was first introduced by Roberts et al. in 2015 to account for acute glycemic response relative to long-term glucose control, demonstrating superior prognostic value in critically ill patients compared to absolute blood glucose (11). Since then, numerous studies have consistently validated and expanded on its clinical significance across diverse patient populations and conditions, including cardiovascular diseases (21, 22), infectious diseases (12), and cerebrovascular disorders (16, 17, 23). Among these, intracerebral hemorrhage (ICH) has garnered particular attention, as SHR has been increasingly recognized for its predictive value in hematoma expansion and neurological deterioration. A multicenter analysis of 71,333 ICH patients revealed that elevated SHR was linked to higher in-hospital mortality (OR 2.07, P < 0.001) and hematoma expansion (OR 1.24, P < 0.05). SHR outperformed glycemic gap and absolute blood glucose, achieving the highest predictive accuracy for in-hospital mortality (AUC = 0.88) (16). In a separate analysis of 880 ICH patients from the MIMIC-IV database, the 5-day maximum SHR exhibited superior predictive performance for both in-hospital and 1-year mortality compared to admission glucose and glycemic gap. Interestingly, this association was more pronounced in younger patients, suggesting that acute hyperglycemia may trigger a more aggressive inflammatory response in this population (17). In aneurysmal subarachnoid hemorrhage (aSAH), SHR has been identified as a significant predictor of poor neurological outcomes. A two-center retrospective study of 127 aSAH patients indicated that those in the highest SHR tertile had a 4.12-fold increased risk of poor functional outcomes at 12 months, independent of diabetes status (23). This highlights SHR’s potential role in reflecting systemic stress responses that contribute to vasospasm, delayed cerebral ischemia (DCI), and secondary brain injury in aSAH patients.

The possible mechanisms linking stress hyperglycemia (SH) to the progression of hemorrhagic stroke, including ICH and SAH, involve a combination of inflammatory responses, oxidative stress, vascular endothelial injury, and immune dysfunction. Acute ICH rapidly activates the sympathetic nervous system (SNS) and the hypothalamic-pituitary-adrenal (HPA) axis, leading to the secretion of catecholamines and cortisol, which induce SH (24, 25). This elevation in blood glucose levels triggers the nuclear factor-κB (NF-κB) signaling pathway, resulting in increased expression of pro-inflammatory cytokines such as tumor necrosis factor-α (TNF-α), interleukin-6 (IL-6), and IL-1β (25, 26). The persistent activation of NF-κB further amplifies inflammation in the perilesional tissue, promoting a cytokine storm and exacerbating neuronal apoptosis. Additionally, high mobility group box 1 (HMGB1) released post-ICH binds to the receptor for advanced glycation end-products (RAGE), perpetuating NF-κB activation and intensifying inflammatory cascades (25, 27). In SAH, sustained elevations of IL-1β and TNF-α aggravate cerebrovascular endothelial dysfunction, leading to increased blood-brain barrier (BBB) permeability, vasospasm, and delayed cerebral ischemia (DCI), contributing to neurological damage (28).

SH significantly enhances reactive oxygen species (ROS) production through multiple interconnected mechanisms, resulting in vascular and neuronal injury. High glucose levels activate NADPH oxidase (NOX), particularly the NOX4 isoform, which promotes intracellular ROS generation in endothelial cells, as demonstrated in human umbilical vein endothelial cells (HUVECs) under hyperglycemic conditions (29, 30). Concurrently, hyperglycemia upregulates myeloperoxidase (MPO) activity, further exacerbating ROS accumulation and oxidative stress, which is closely associated with vascular endothelial dysfunction (31). ROS overproduction leads to mitochondrial dysfunction by damaging mitochondrial membranes, reducing membrane potential, and inducing the release of cytochrome C (Cyt C). This cascade activates caspase-9 and caspase-3, driving apoptosis and worsening tissue injury (29). Additionally, ROS disrupt BBB integrity by altering the expression and distribution of tight junction proteins, increasing permeability and contributing to cerebral edema (32).

In ICH models, superoxide production correlates with elevated matrix metalloproteinase-9 (MMP-9) expression, which degrades the basement membrane, further weakening BBB integrity and significantly heightening the risk of secondary hemorrhage and hematoma expansion (33). The link between SH and hematoma expansion likely stems from vascular endothelial injury and inflammatory responses. Hyperglycemia enhances the expression of MMP-9 and NF-κB, undermining vascular wall integrity and increasing the probability of hematoma growth (34). Moreover, aquaporin-4 (AQP4), the most abundant water channel protein in brain tissue, is crucial for BBB maintenance and cerebral edema mitigation. However, hyperglycemia downregulates AQP4 expression, exacerbating cerebral edema and brain injury following ICH (35). SH also compromises immune defense by impairing neutrophil chemotaxis, phagocytosis, and bactericidal activity, reducing the body’s capacity to fend off infections (36). Hyperglycemia inhibits the PI3K/Akt/mTOR pathway, suppressing T cell proliferation and differentiation, thereby weakening immune responses and increasing susceptibility to infections (37). Furthermore, hyperglycemia suppresses complement system activation, reducing the production of C3 convertase and the membrane attack complex (MAC), further diminishing antimicrobial defense mechanisms (38).

In our study, patients in the highest SHR tertile (T3) exhibited a greater prevalence of comorbidities, organ dysfunction, and systemic inflammation, aligning with prior evidence that links acute hyperglycemia to adverse outcomes in critical illnesses (39, 40). Notably, the increased rates of AKI, sepsis, and liver dysfunction observed in T3 emphasize the systemic nature of stress hyperglycemia and its contribution to multiorgan impairment. The strong association between SHR and ACM, which persisted across all adjustment models, further validates the role of hyperglycemia in amplifying inflammatory responses and endothelial dysfunction, ultimately exacerbating patient outcomes (41). The linear relationship between SHR and mortality, as demonstrated by RCS regression, indicates a proportional increase in risk without evidence of a threshold effect. This suggests that even moderate elevations in SHR contribute incrementally to mortality, highlighting the potential benefit of incorporating SHR monitoring into routine clinical practice. The proportional hazards assumption was tested using Schoenfeld residuals. While the assumption held for ICU, in-hospital, 30-day, and 90-day mortality models, minor violations were observed for 180-day and 1-year outcomes. These were not considered to substantially bias the findings, but they highlight the need for cautious interpretation of long-term hazard ratios and the potential value of future time-dependent modeling.

Previous studies have shown that stress-induced hyperglycemia reflects the body’s response to physiological insults, but when disproportionate, it can promote thrombosis, oxidative stress, and further neurological injury (42). Subgroup analyses reinforced the consistency of SHR’s predictive value across diverse patient profiles, with elevated SHR correlating with higher mortality regardless of age, gender, or GCS score. The heightened risk in surgical patients underscores the compounded impact of metabolic stress and surgical trauma, suggesting that this cohort may benefit from closer glycemic management. Interestingly, while no significant interaction was found between SHR and surgical intervention for early mortality outcomes (ICU and in-hospital mortality), a significant interaction emerged for long-term outcomes, including 30-day, 180-day, and 1-year mortality. This suggests that although surgical stress may not immediately amplify the detrimental effects of stress hyperglycemia, it could contribute to a delayed impact on patient prognosis over time. One possible explanation is that surgical procedures, particularly in the context of hemorrhagic stroke, induce sustained metabolic stress and inflammatory activation. These physiological responses may synergize with elevated glucose fluctuations to exacerbate endothelial dysfunction, impair organ recovery, and promote secondary complications such as infections or thrombotic events. In this context, SHR may serve not only as a marker of acute stress but also as a predictor of poor recovery trajectories in post-surgical patients. These findings underscore the need for closer glycemic monitoring and potentially more aggressive glucose management strategies in surgical HS patients with elevated SHR. Further studies are warranted to explore the underlying mechanisms and to determine whether early intervention targeting stress hyperglycemia can improve long-term outcomes in this population.

Furthermore, it is important to consider the possibility of reverse causality. Rather than SHR being a direct driver of poor prognosis, elevated SHR may instead reflect the severity of the underlying critical illness. In critically ill patients, heightened stress responses—mediated by systemic inflammation, sympathetic nervous system activation, and hypothalamic-pituitary-adrenal (HPA) axis stimulation—can lead to transient hyperglycemia, even in the absence of pre-existing diabetes. This acute metabolic response may serve as a marker of physiological decompensation, rather than being an independent pathophysiological factor. Given the retrospective and observational nature of our study, causal inference remains limited. Therefore, future mechanistic studies and interventional trials are warranted to clarify whether stress hyperglycemia is simply a surrogate marker or a modifiable risk factor contributing directly to increased mortality in patients with hemorrhagic stroke.

Paradoxically, certain indicators such as lower age, reduced RBC counts, descended sodium and lower HbA1c levels were observed in the highest SHR tertile. This seemingly contradictory observation may reflect the acute and transient nature of hyperglycemia in younger patients with fewer baseline comorbidities, who may exhibit robust metabolic responses to stress. Alternatively, the lower HbA1c levels in T3 may indicate that patients with previously well-controlled glucose levels experience exaggerated hyperglycemia during acute illness, emphasizing the disproportionate impact of acute glycemic excursions in otherwise metabolically healthy individuals (43).

Interestingly, we also observed a lower prevalence of hyperlipidemia in the highest SHR tertile (T3) compared to T1 and T2. One plausible explanation for this counterintuitive finding is the presence of acute metabolic disturbances in critically ill patients. During acute illness, especially in the context of hemorrhagic stroke or intensive care, systemic inflammatory responses and metabolic stress can suppress hepatic lipid synthesis and accelerate lipid catabolism, leading to transient reductions in serum lipid levels such as total cholesterol and triglycerides. This phenomenon has been well-documented in critical care settings and may lead to underestimation or underdiagnosis of pre-existing hyperlipidemia, particularly in patients with high SHR values (44, 45). Additionally, selection bias may partly explain this inverse association. Patients with extremely high SHR often represent those with more severe disease or metabolic decompensation who may not have had sufficient time for complete clinical history documentation, including lipid profiles. In emergency or unconscious states, the lack of prior medical records could result in misclassification of chronic conditions such as hyperlipidemia. Moreover, preadmission use of lipid-lowering agents like statins—more common in patients with cardiovascular risk—might also reduce lipid levels or prevent a clinical diagnosis of hyperlipidemia during ICU admission. Finally, survivorship bias cannot be excluded. Patients with both high SHR and uncontrolled dyslipidemia may have died before hospital admission or lacked sufficient data for inclusion, thus skewing the distribution. Together, these factors may account for the unexpected inverse relationship observed between SHR and hyperlipidemia. Further research is warranted to delineate the impact of acute-phase metabolic responses and potential selection artifacts in this context.

The prolonged ICU and hospital stay observed in T3 patients reflect the increased severity and complexity of their clinical course. The association between elevated SHR and greater need for mechanical ventilation, vasopressor support, and surgical intervention underscores the relationship between metabolic dysregulation and the escalation of care requirements. This aligns with earlier research suggesting that hyperglycemia in critical illness is not merely a reflection of disease severity but actively contributes to worse outcomes through mechanisms such as endothelial dysfunction, immune dysregulation, and impaired tissue perfusion (46, 47).

## Limitations and future directions

Despite the robustness of our findings, certain limitations must be acknowledged. First, this study included only critically ill hemorrhagic stroke (HS) patients admitted to the ICU, thereby excluding those with milder forms of HS who did not require intensive care. As stress hyperglycemia is often more pronounced in severely ill patients, this inclusion criterion may exaggerate the observed effects of SHR and limit the generalizability of our findings. Second, patients without available HbA1c measurements were excluded due to the requirement of this parameter for SHR calculation. This may have introduced selection bias, as HbA1c is more likely to be measured in patients with known diabetes or in those who survived long enough for laboratory testing. Consequently, ultra-acute fatalities and certain patient subgroups may have been underrepresented, potentially affecting the representativeness of the study cohort.

Additionally, patients with ICU stays shorter than 24 hours were excluded. While this criterion is commonly used in ICU-based studies to ensure data completeness and exclude extreme cases, it may have inadvertently removed two important groups: (1) patients with extremely severe disease who died shortly after ICU admission, and (2) patients with mild illness requiring only brief observation. Both scenarios could introduce selection bias, potentially shifting the SHR–mortality association toward more stable patients.

There is also the possibility of treatment bias. ICU teams may have implemented glucose control strategies—such as insulin therapy—more aggressively in certain patients, which could have mitigated the harmful impact of stress hyperglycemia on outcomes. However, due to limitations of the database, we lacked information on post-admission glycemic trajectories and insulin treatment, making it difficult to assess whether interventions targeting glucose control influenced the association between SHR and mortality. This represents an important area for future research.

To address these limitations, future studies should focus on prospective, multicenter cohorts to validate the prognostic utility of SHR across diverse patient populations and healthcare settings. Randomized controlled trials (RCTs) assessing the efficacy of targeted interventions, such as glucose modulation strategies or anti-inflammatory therapies, could offer critical insights into whether mitigating SHR improves outcomes in HS patients. Furthermore, mechanistic research at the molecular and cellular levels is warranted to dissect the underlying pathways linking stress hyperglycemia to neuroinflammation, endothelial injury, and secondary brain damage. The integration of advanced imaging modalities and biomarker profiling in future studies may provide a more comprehensive evaluation of metabolic disturbances and their progression over time. By bridging these knowledge gaps, future investigations can enhance risk stratification, refine predictive models, and inform the development of tailored therapeutic approaches aimed at improving survival and functional outcomes in critically ill patients with HS.

## Conclusion

In This study demonstrates a robust and independent association between stress hyperglycemia ratio (SHR) and all-cause mortality across multiple time points in patients with hemorrhagic stroke. While SHR does not directly measure inflammation or organ dysfunction, it may serve as an indirect marker of acute metabolic stress, which is often accompanied by systemic inflammatory responses and multi-organ impairment in critically ill patients. Given the retrospective nature of this study, causality cannot be definitively established, and residual confounding may persist despite multivariable adjustments. Nevertheless, SHR shows promise as a practical and non-invasive tool for early risk stratification in neurocritical care settings, warranting further prospective validation and mechanistic investigation.

## Data Availability

The raw data supporting the conclusions of this article will be made available by the authors, without undue reservation.

## Supplementary material

Supplementary Figure 1 Schoenfeld residuals for icu mortality model.

Supplementary Figure 2 Schoenfeld residuals for in-hospital mortality model.

Supplementary Figure 3 Schoenfeld residuals for 30-day mortality model.

Supplementary Figure 4 Schoenfeld residuals for 90-day mortality model.

Supplementary Figure 5 Schoenfeld residuals for 180-day mortality model.

Supplementary Figure 6 Schoenfeld residuals for 1-year mortality model.
