# Supplementary figures and images for: Unveiling the role of stress hyperglycemia in predicting mortality for critically ill hemorrhagic stroke patients: insights from MIMIC-IV
Source: Front Endocrinol (Lausanne). 2025 May 2;16:1558352. doi: 10.3389/fendo.2025.1558352 (PMC12081252; doi:10.3389/fendo.2025.1558352)

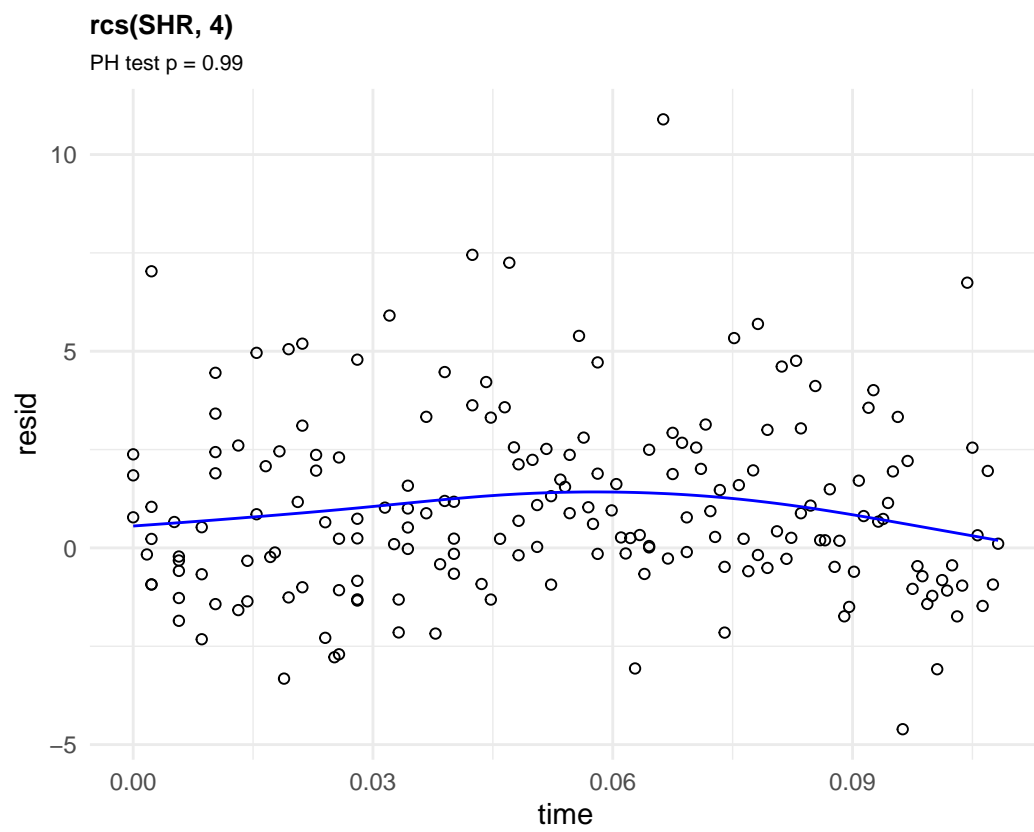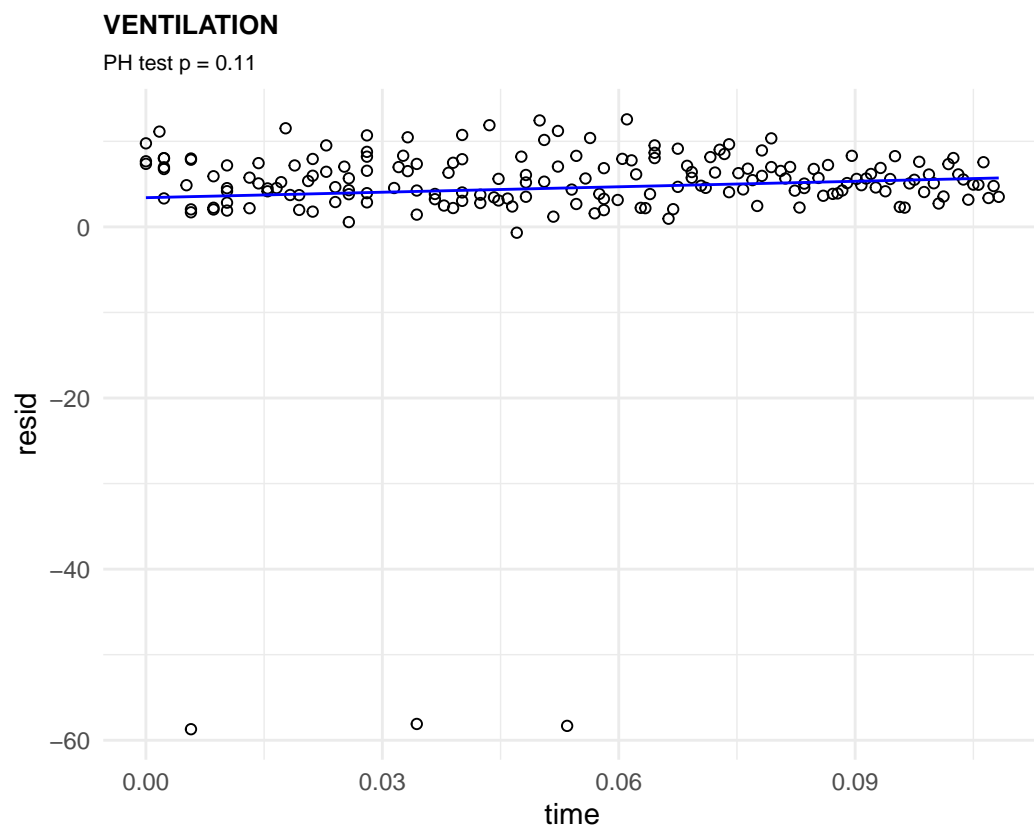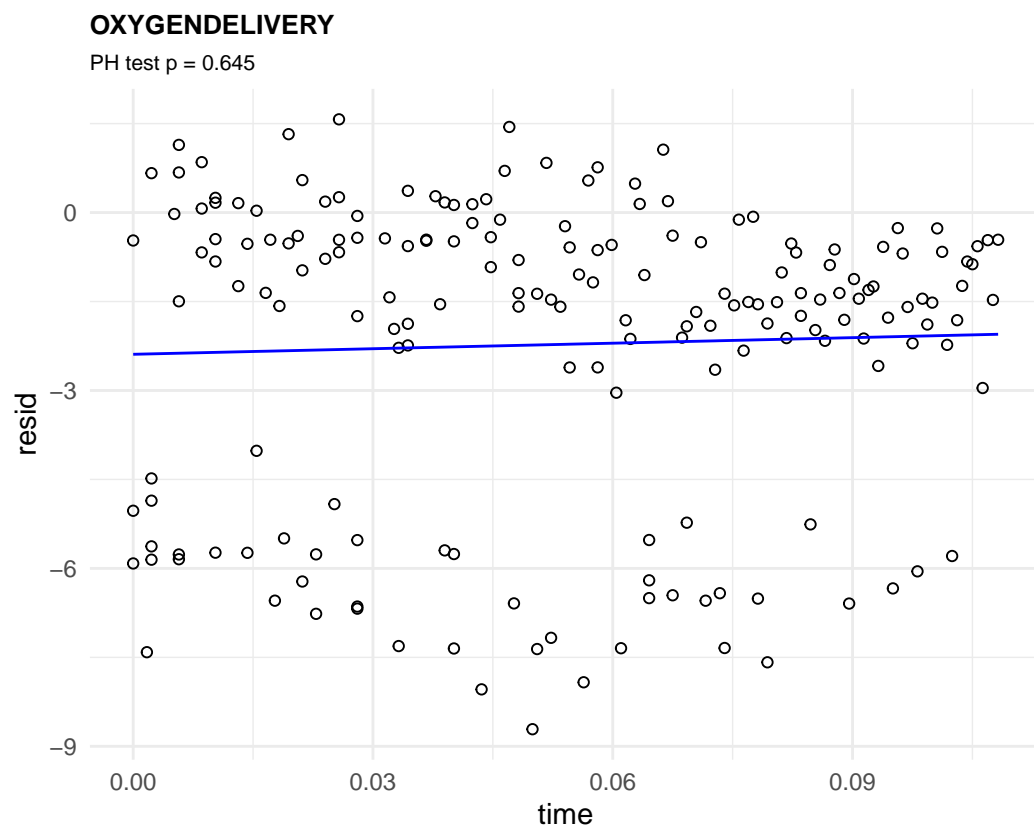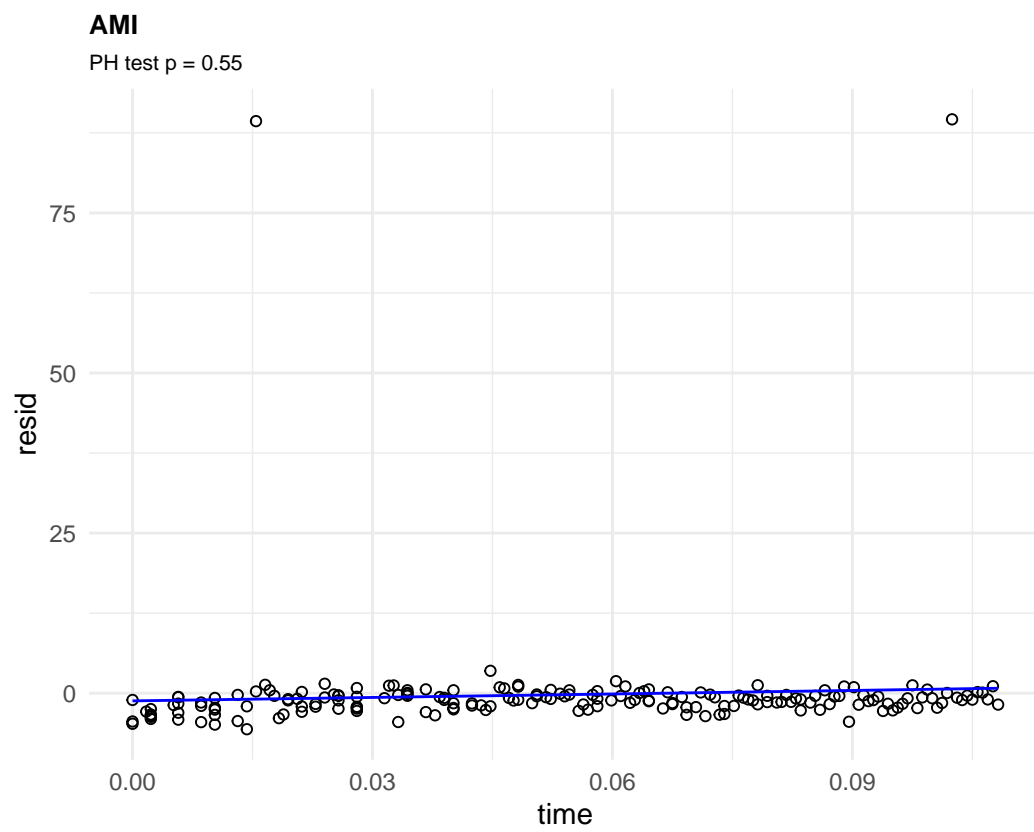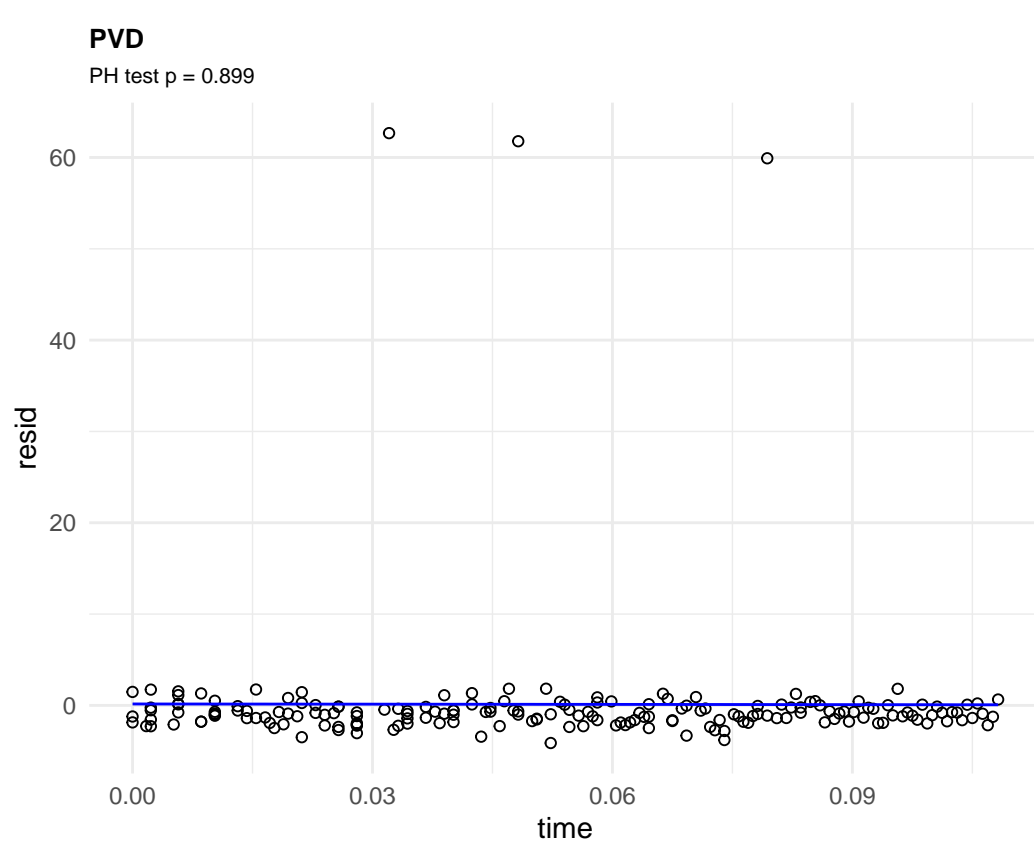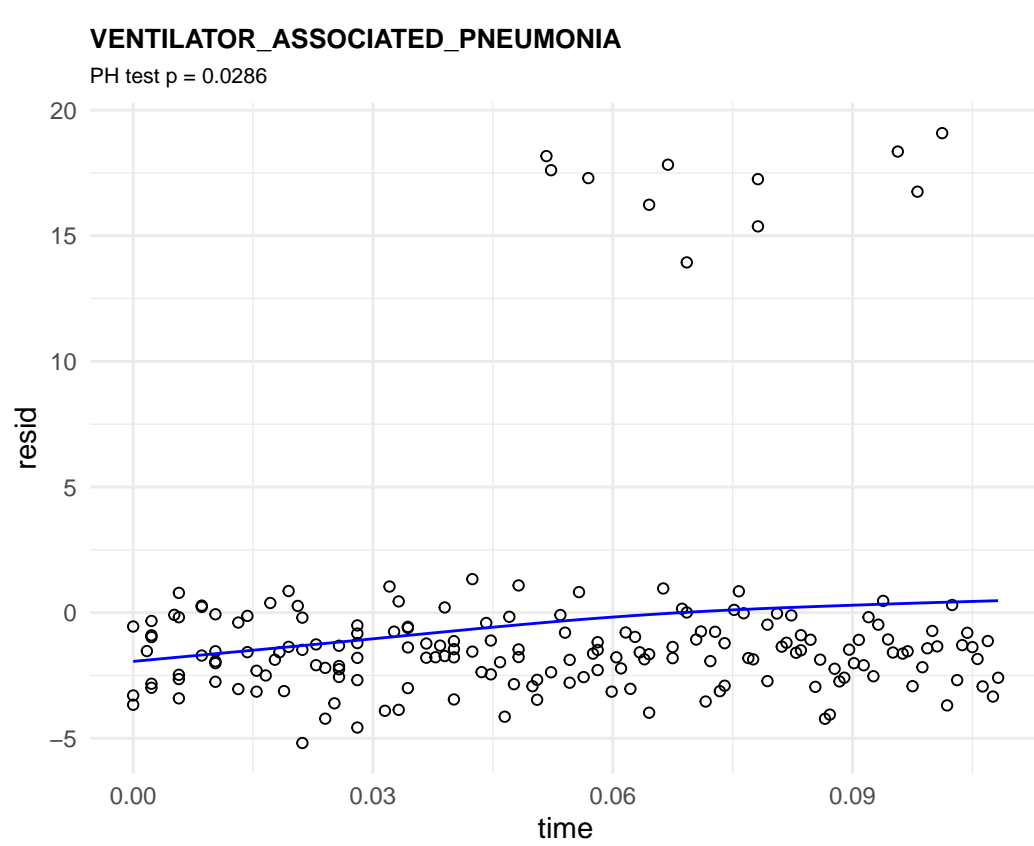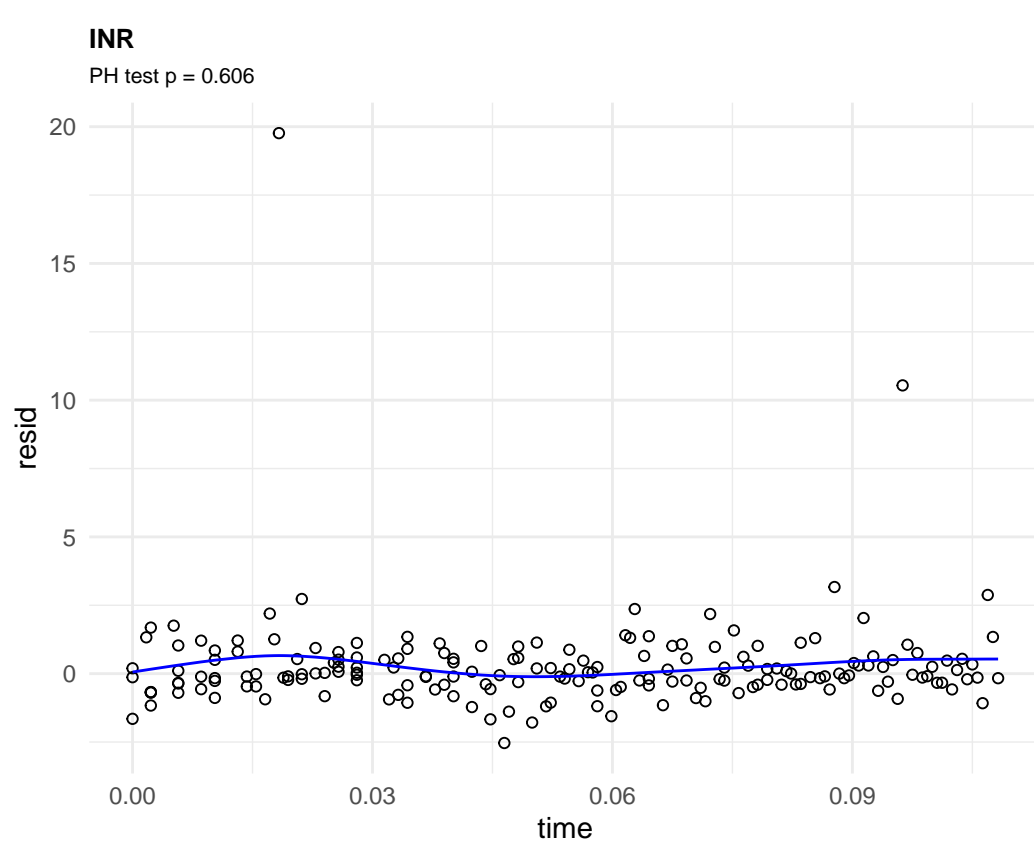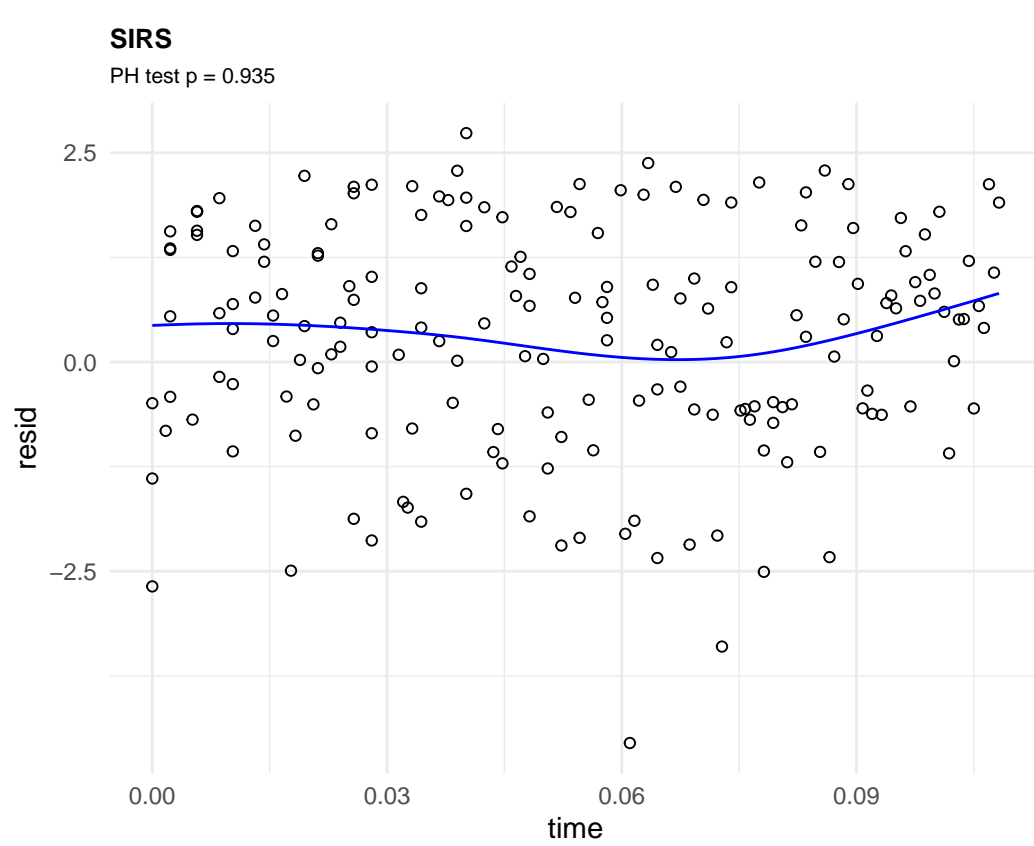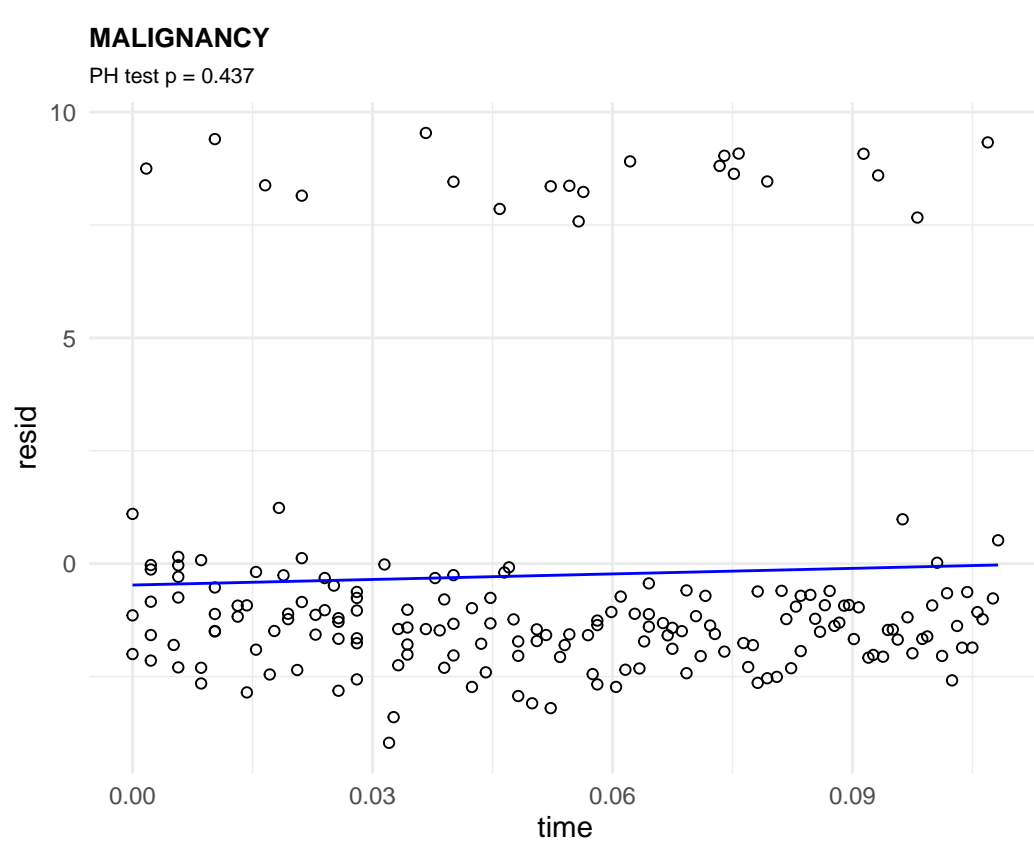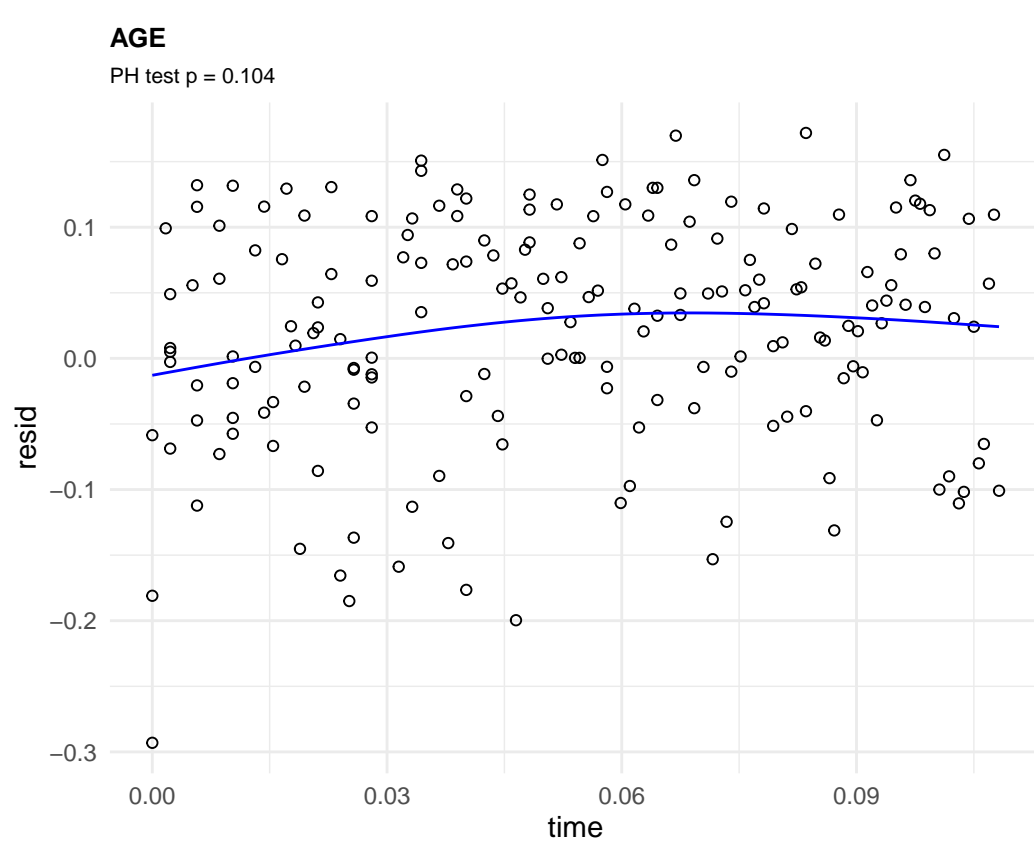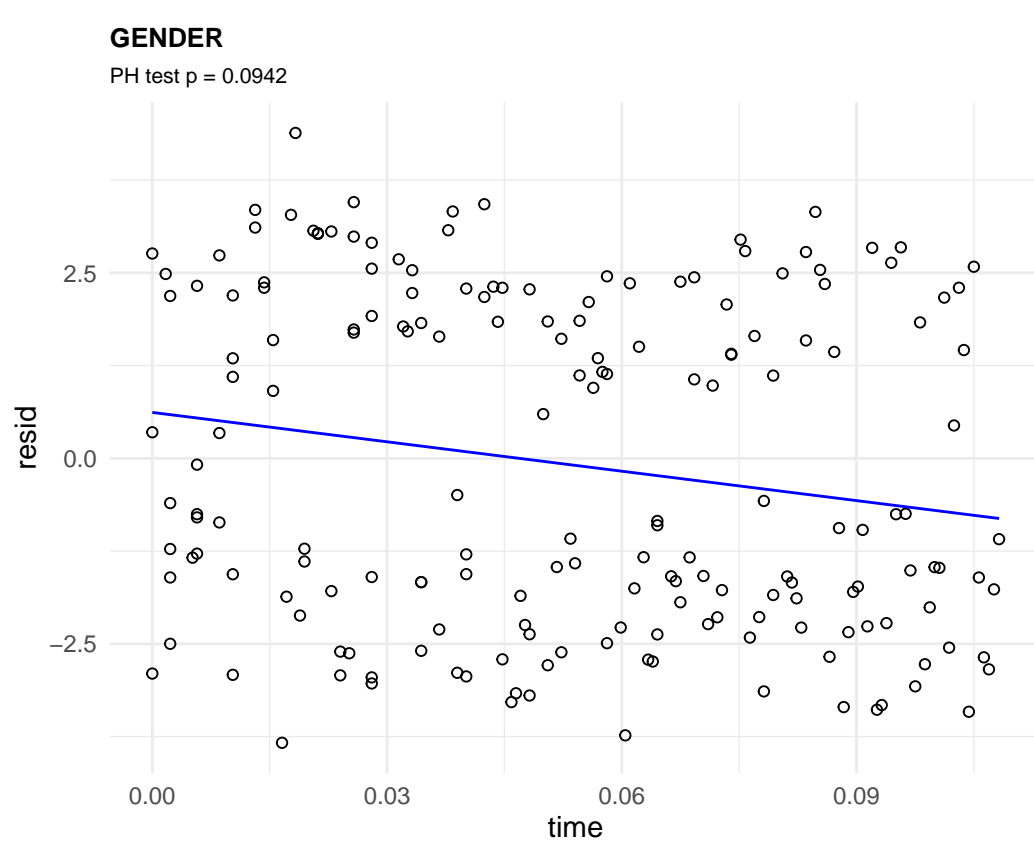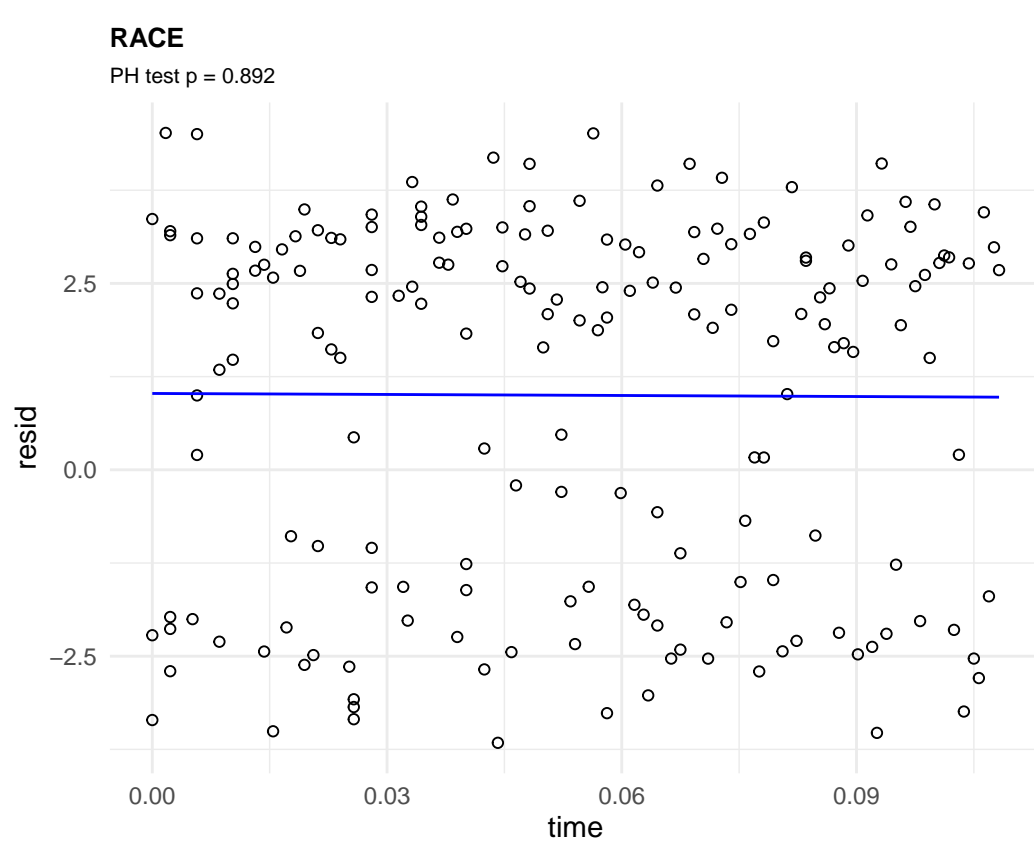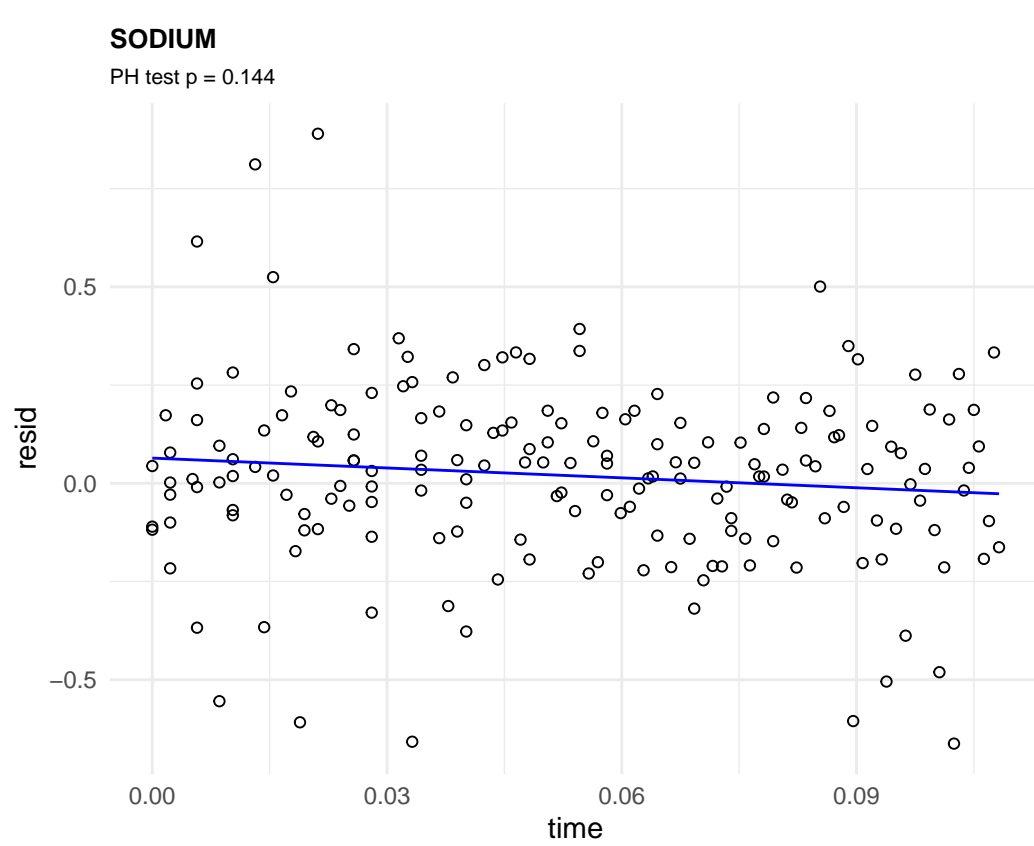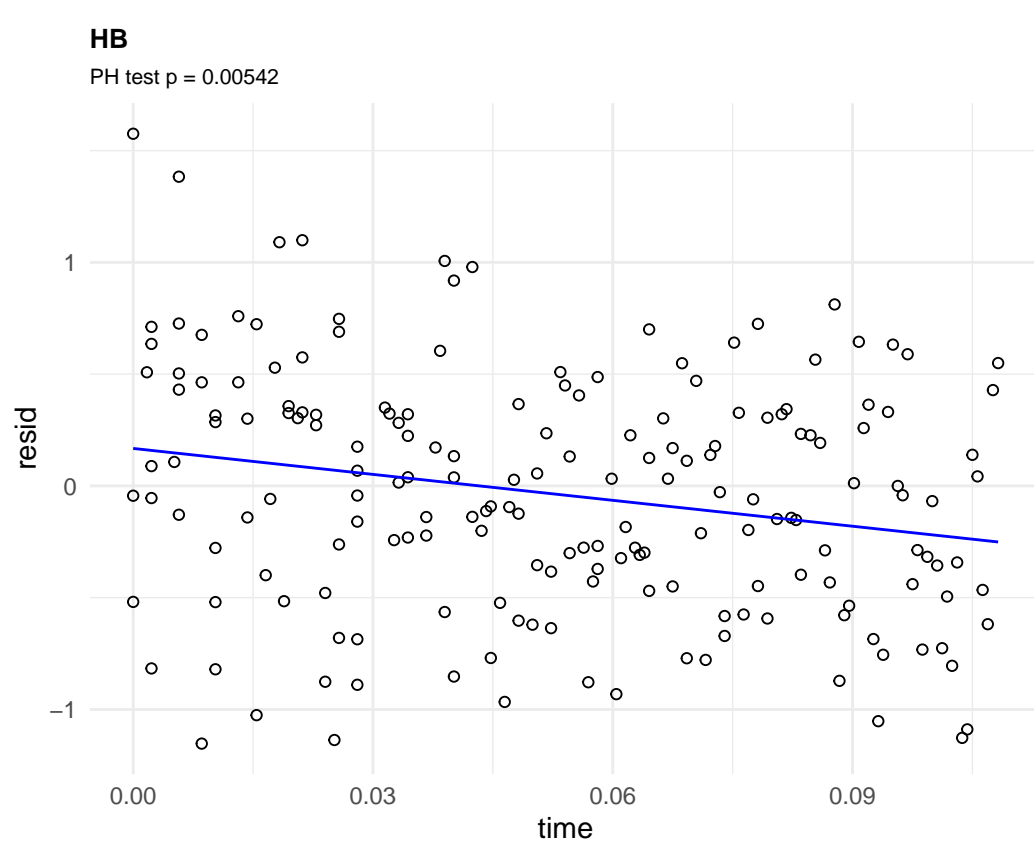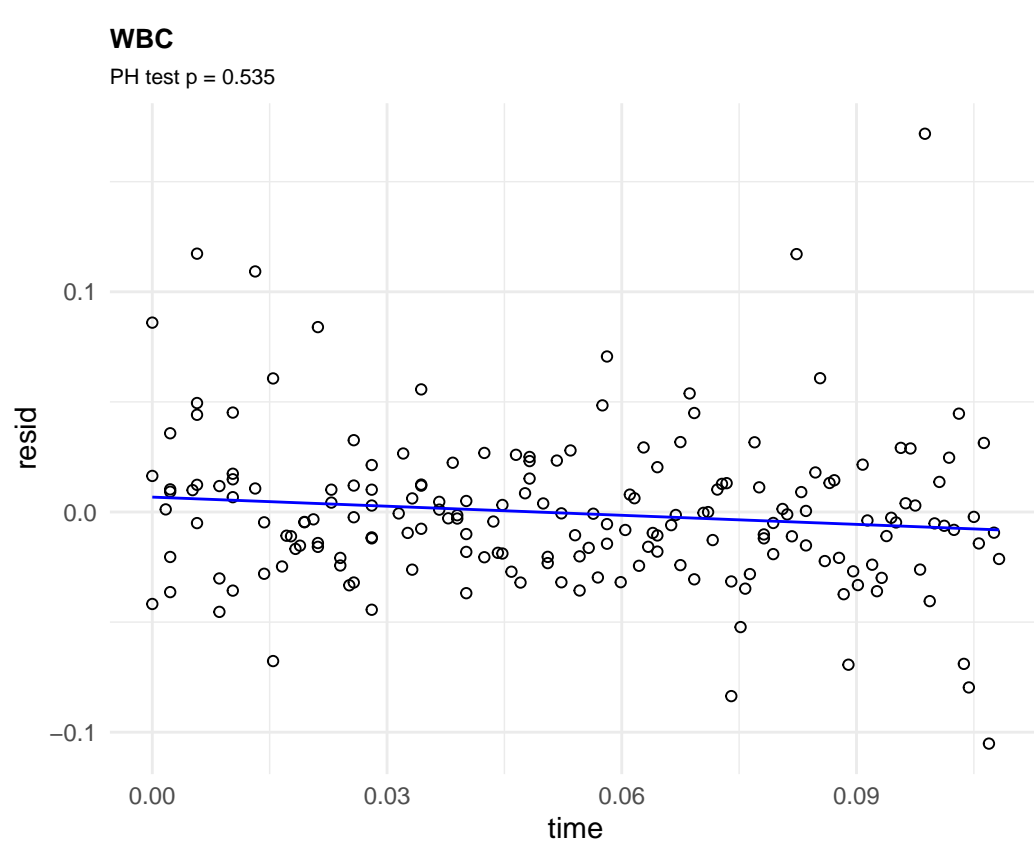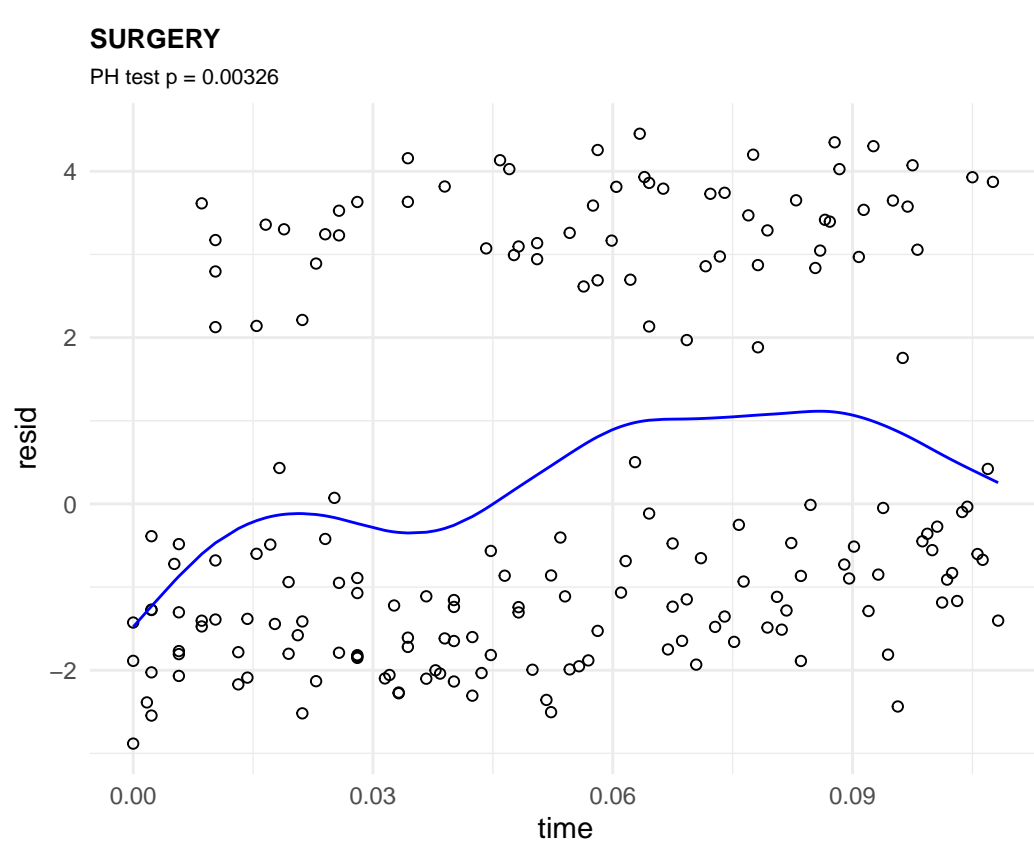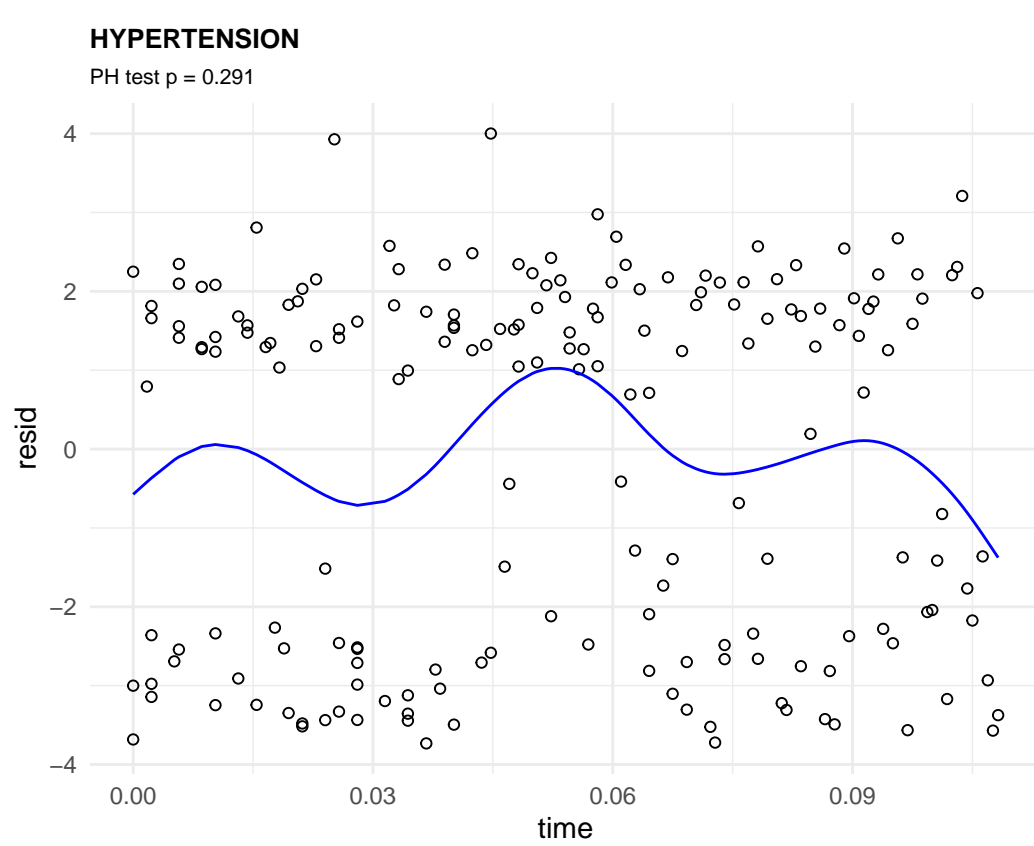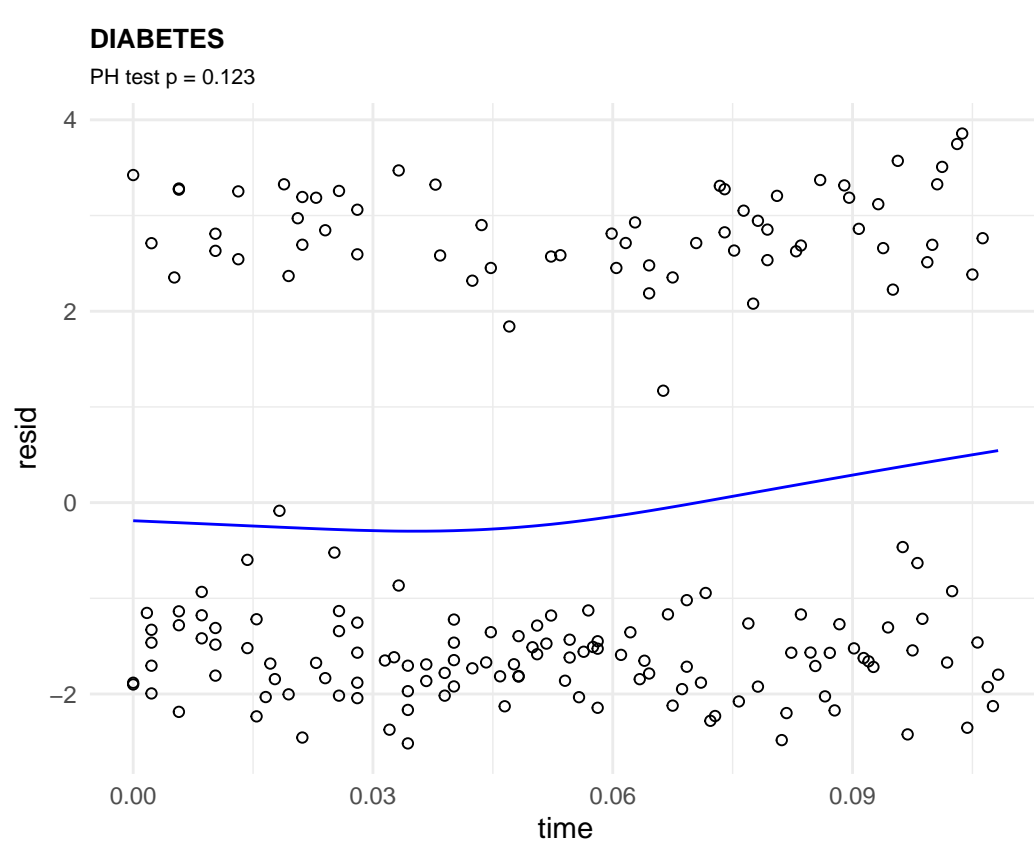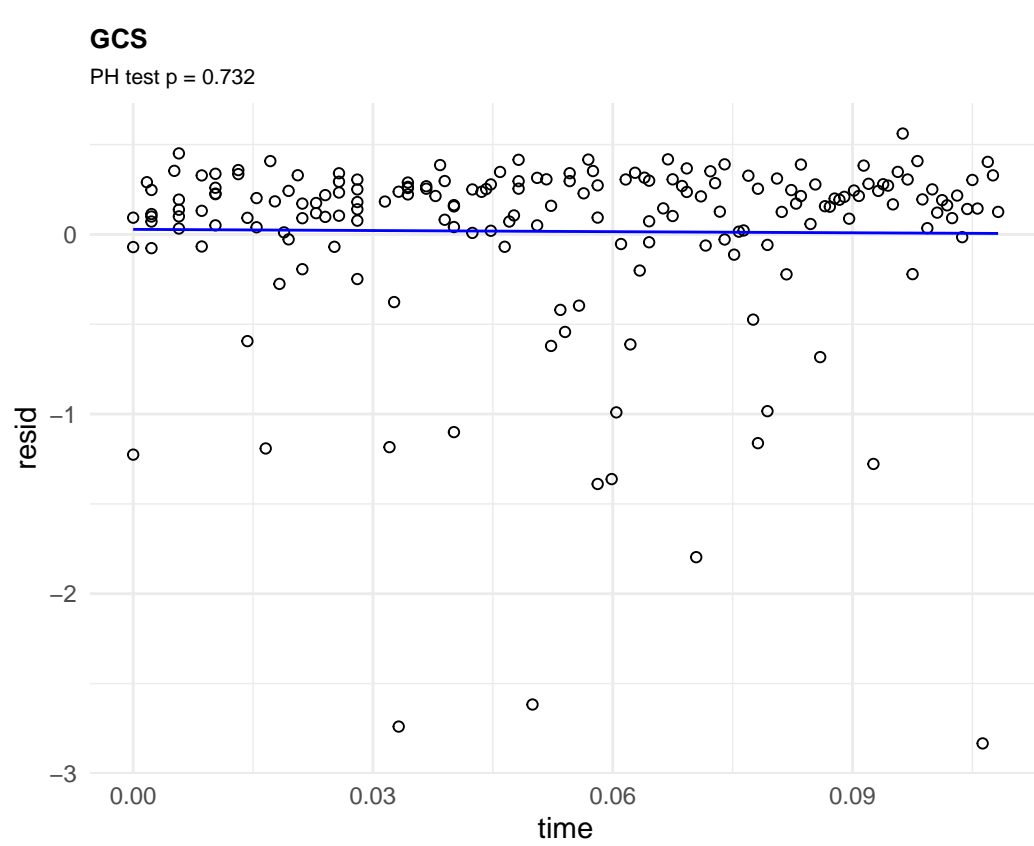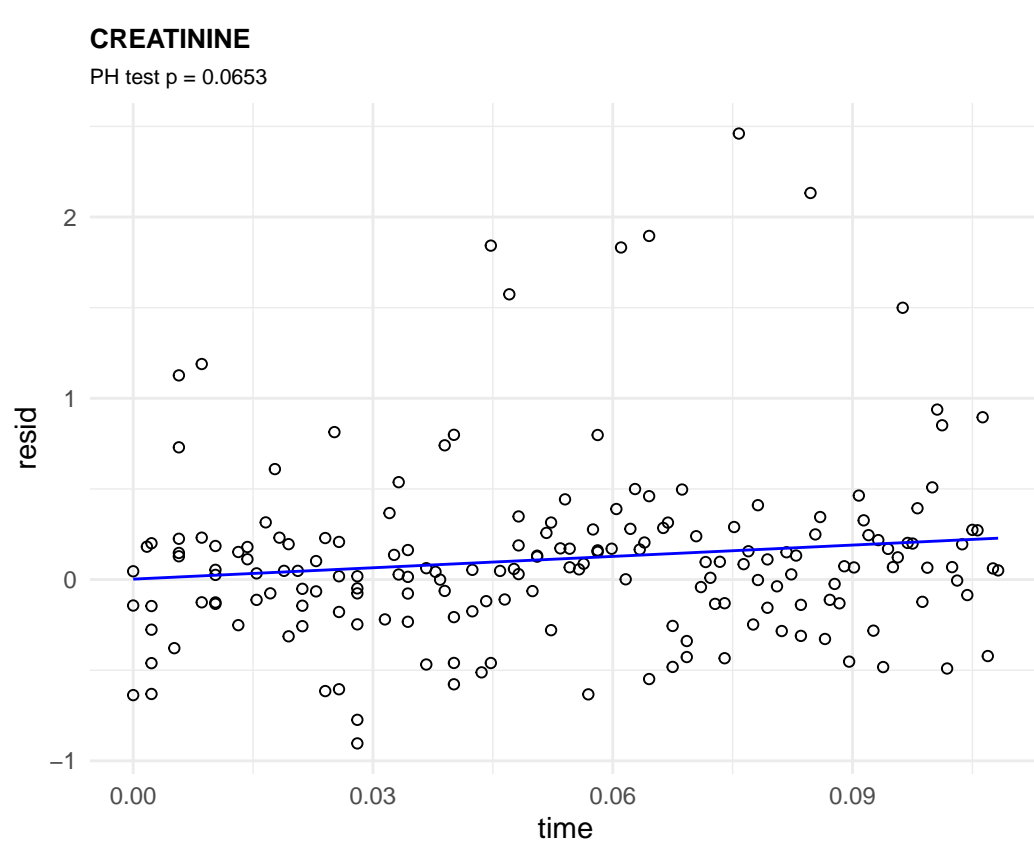

Supplement: Supplementary Figure 1 — Schoenfeld residuals for icu mortality model. [file DataSheet1.pdf]

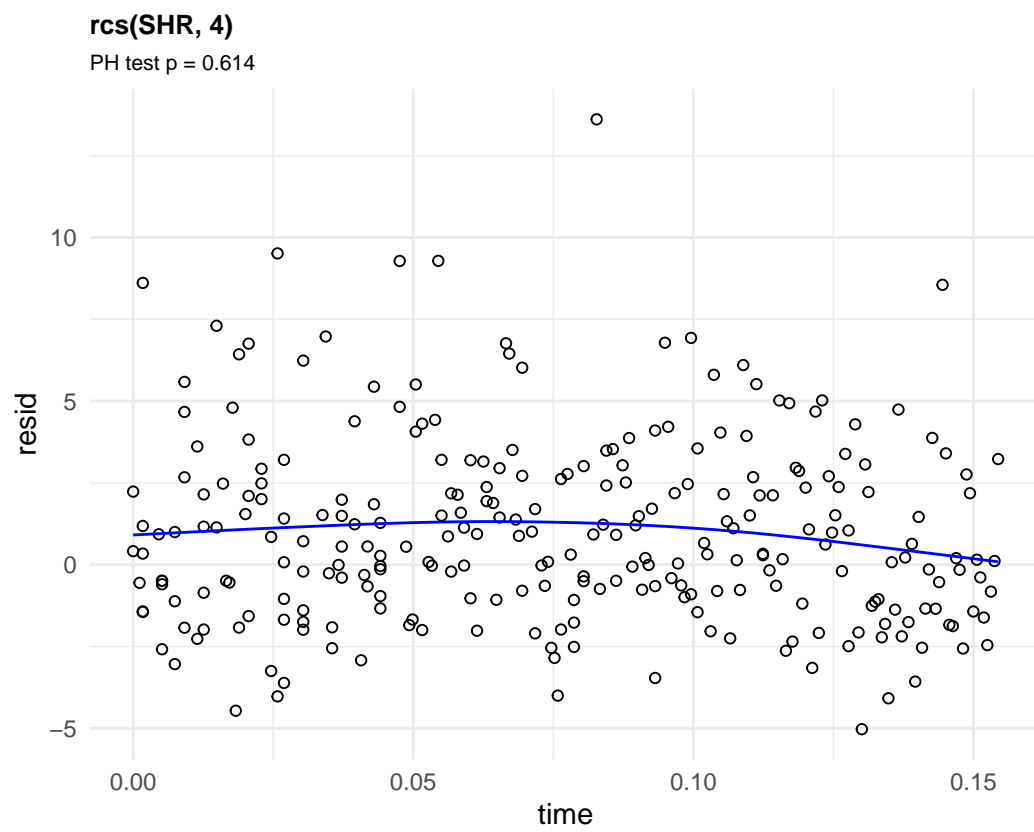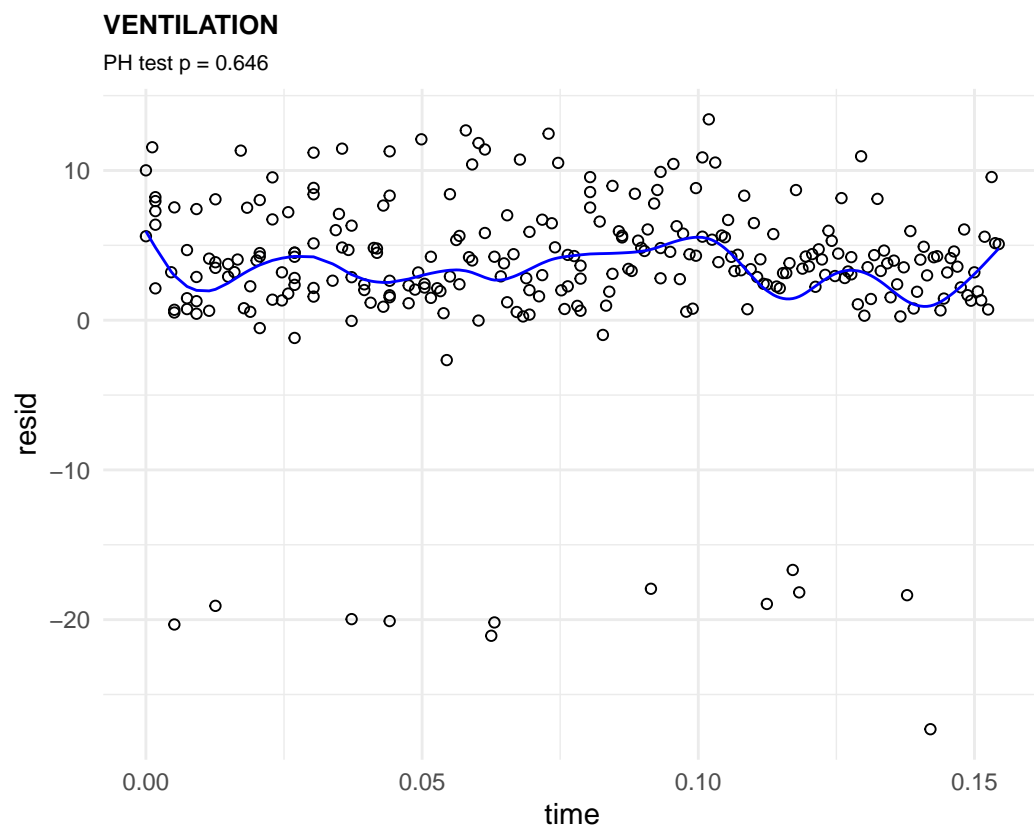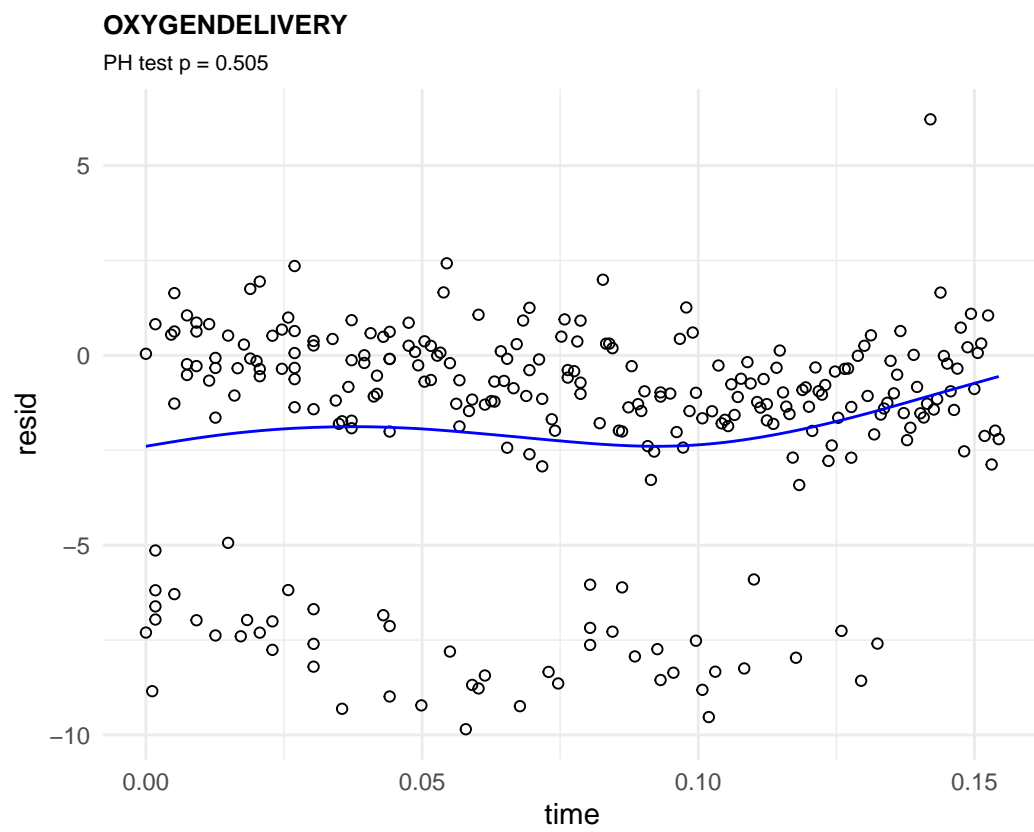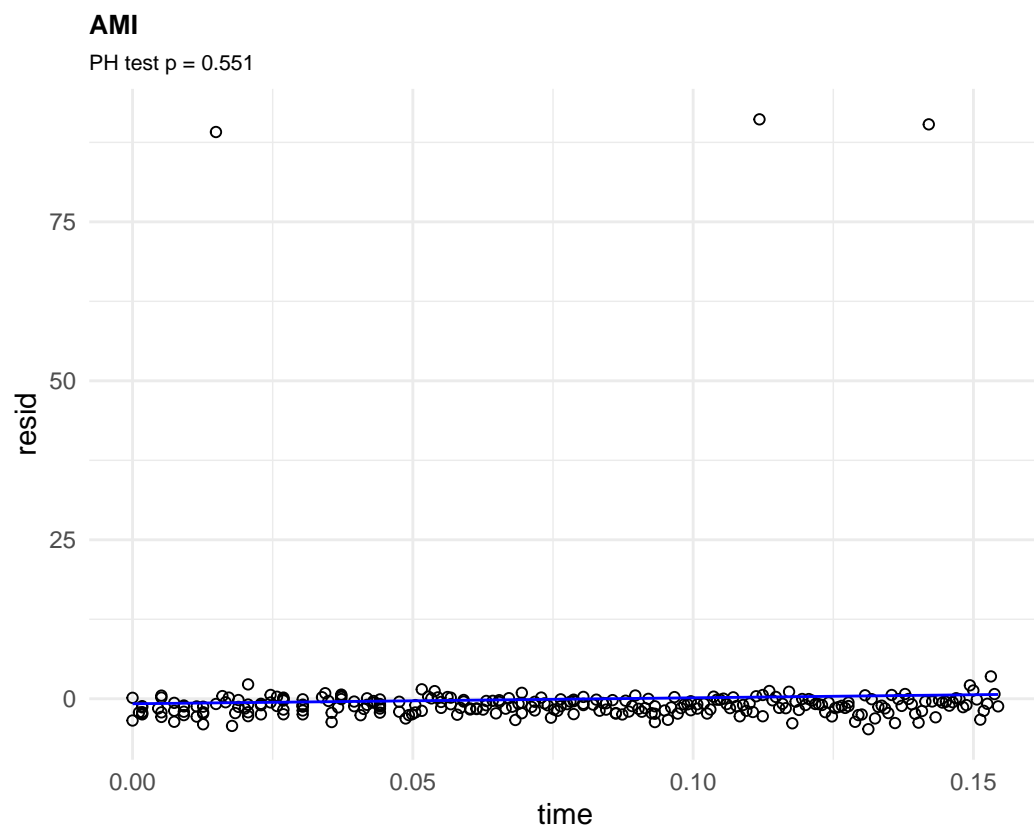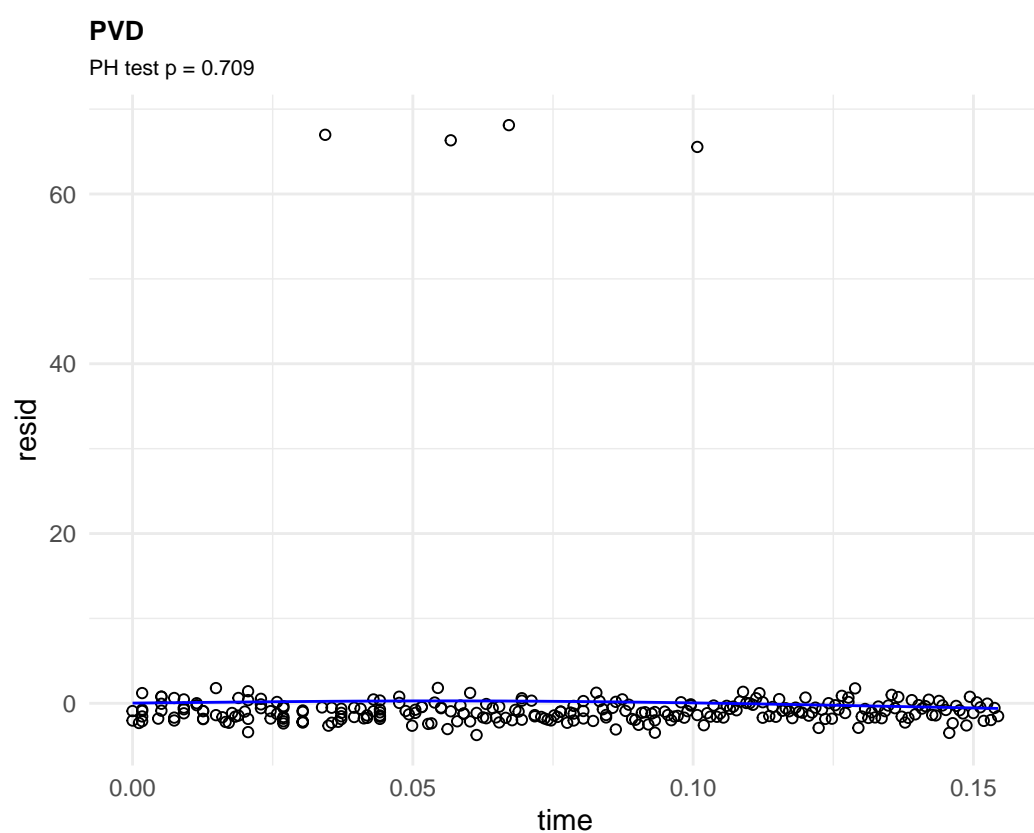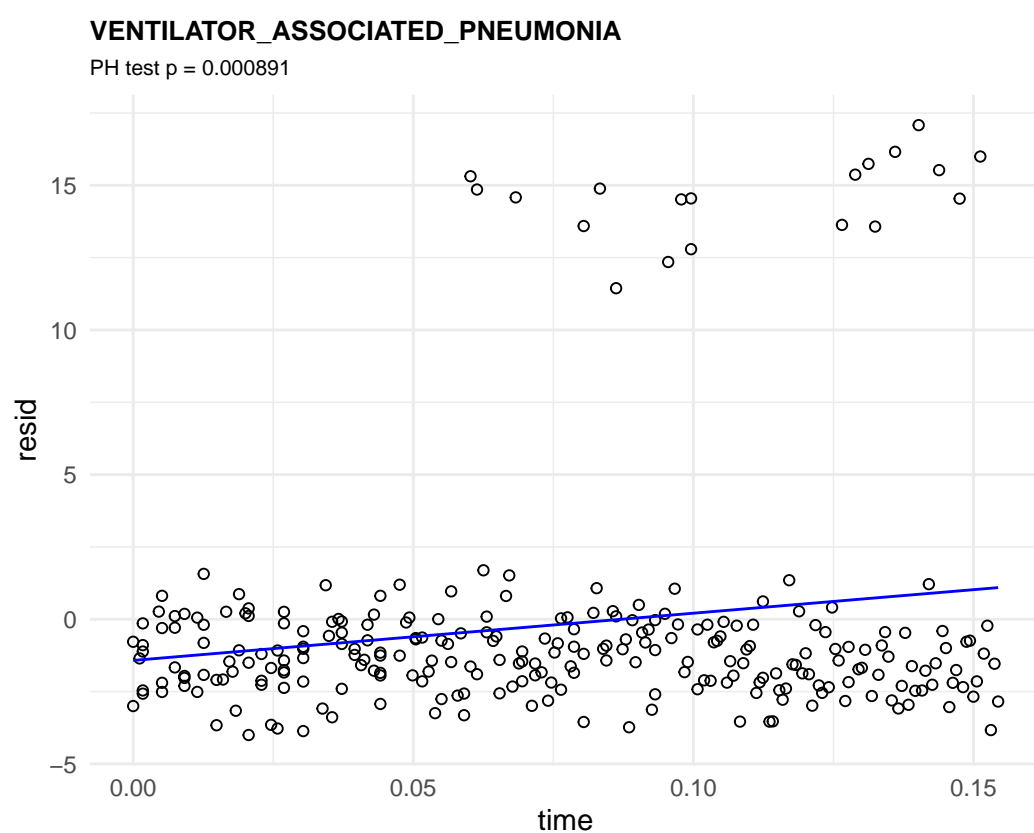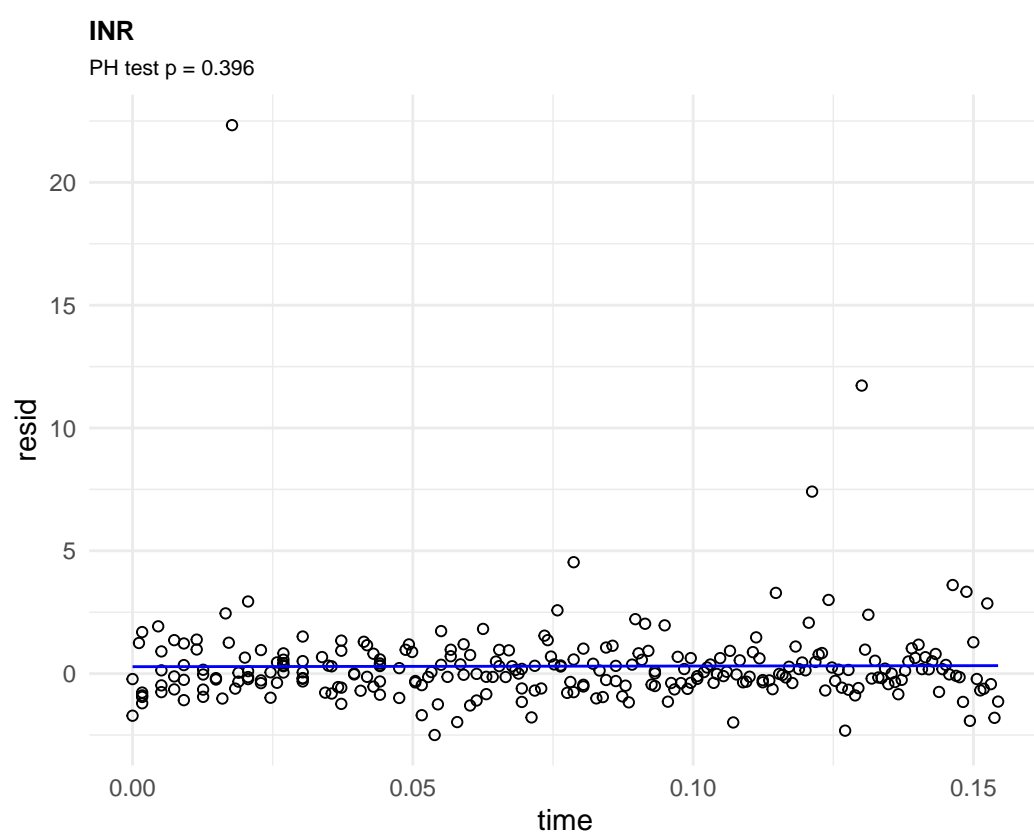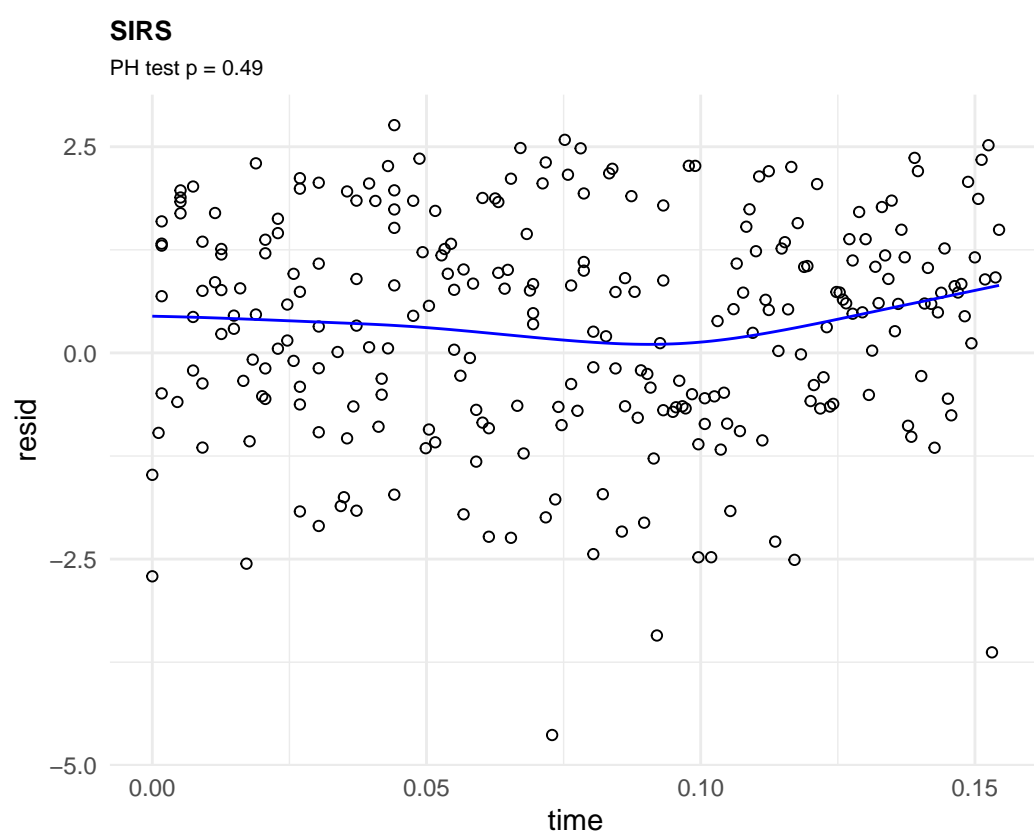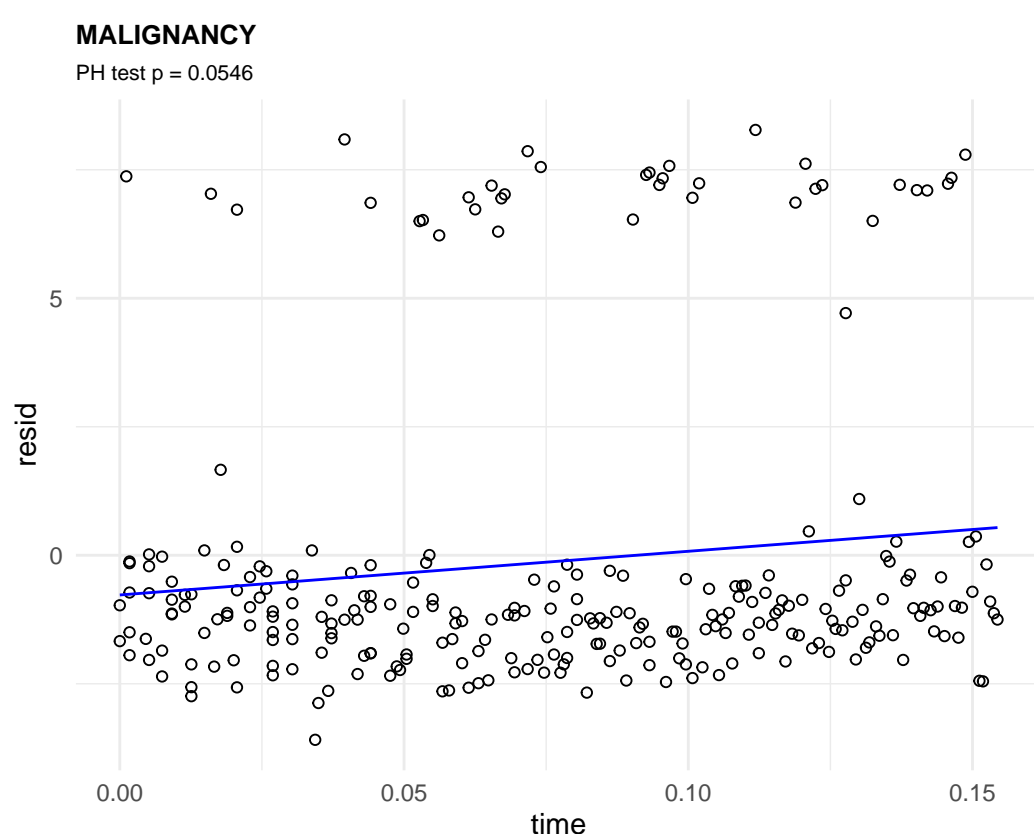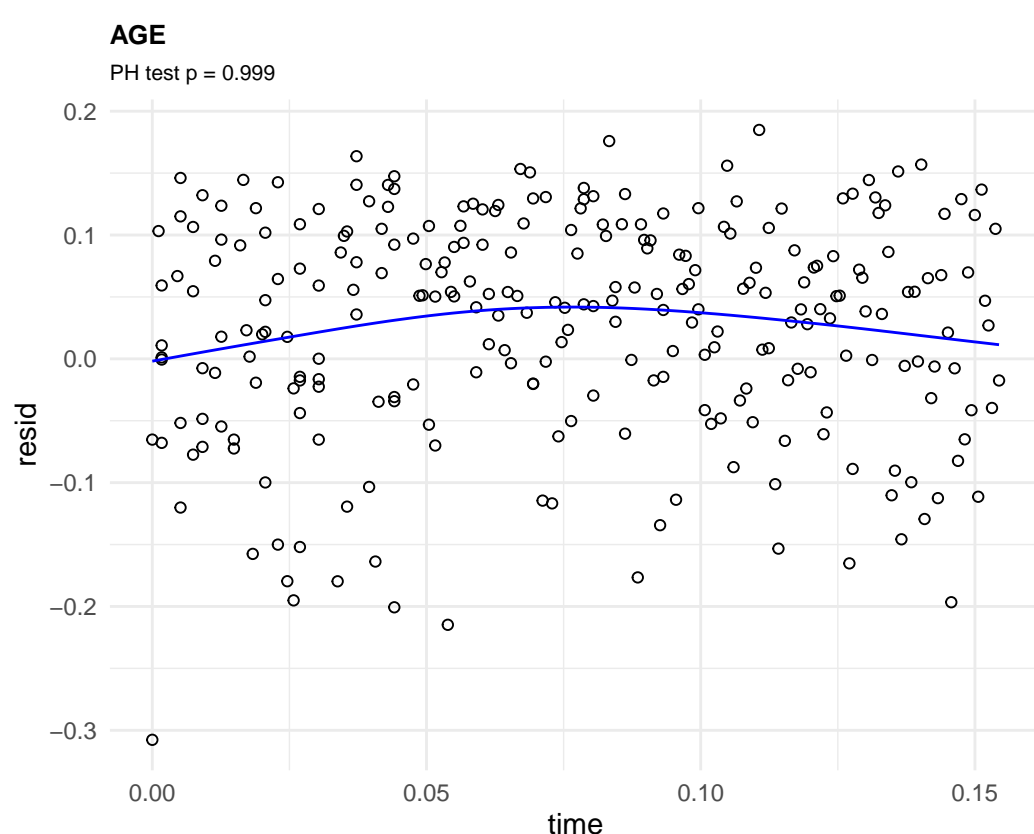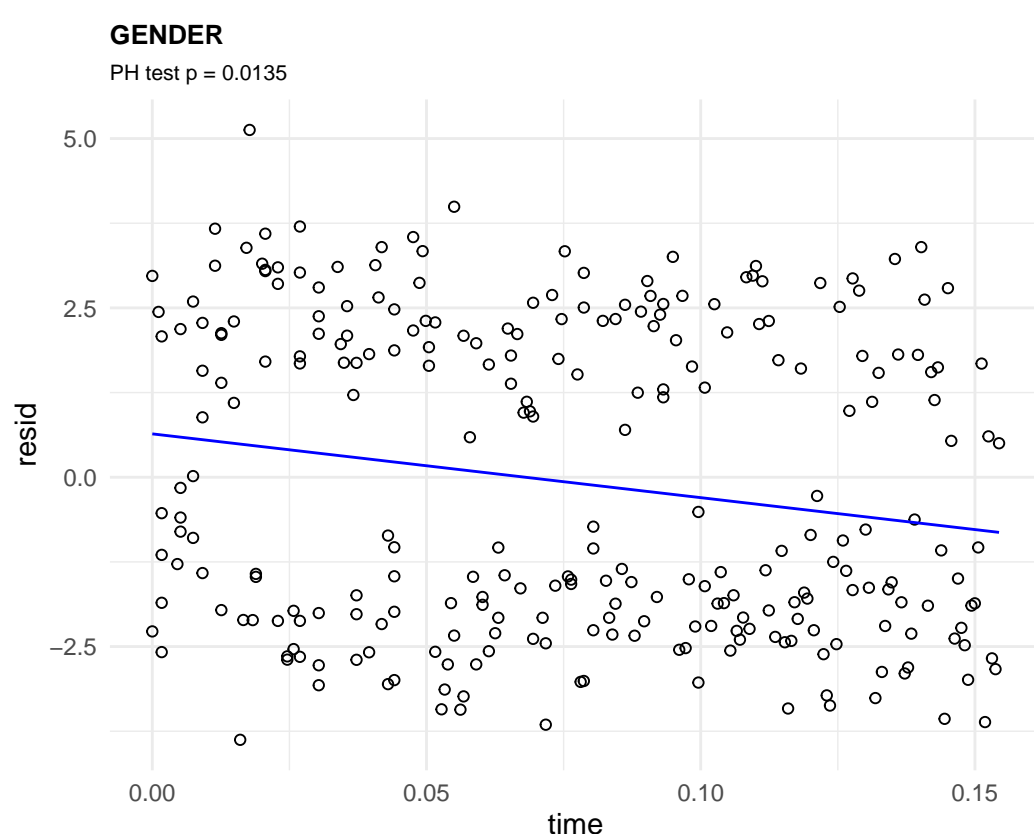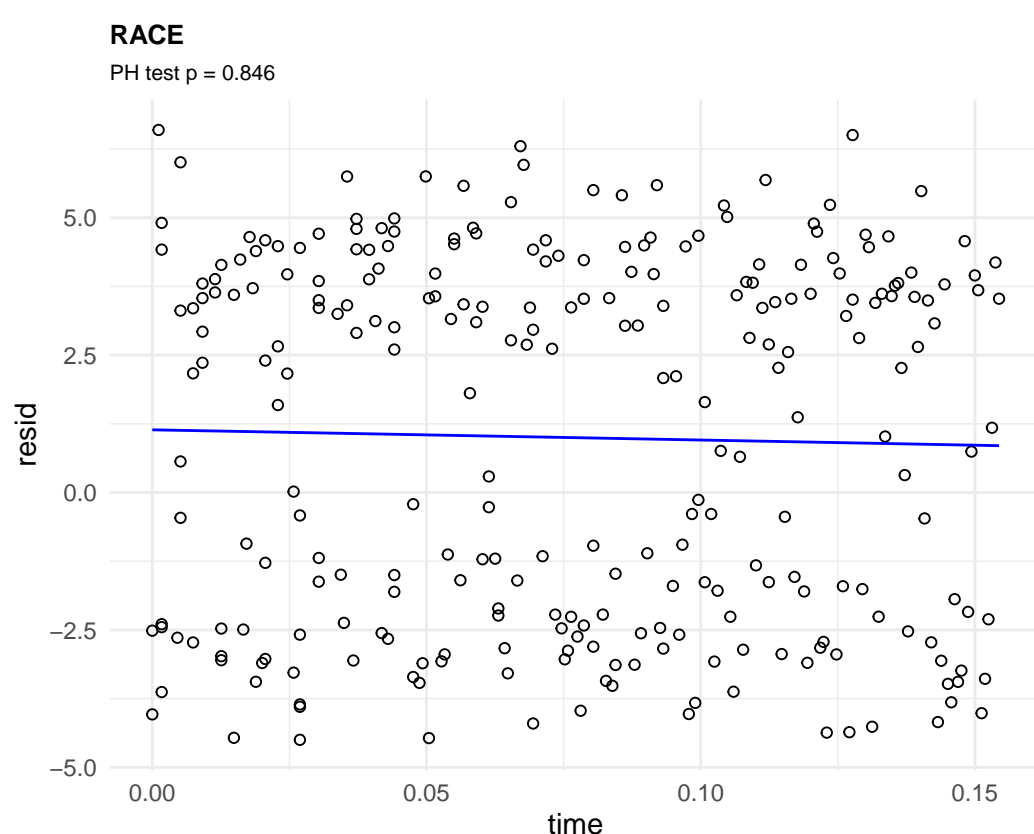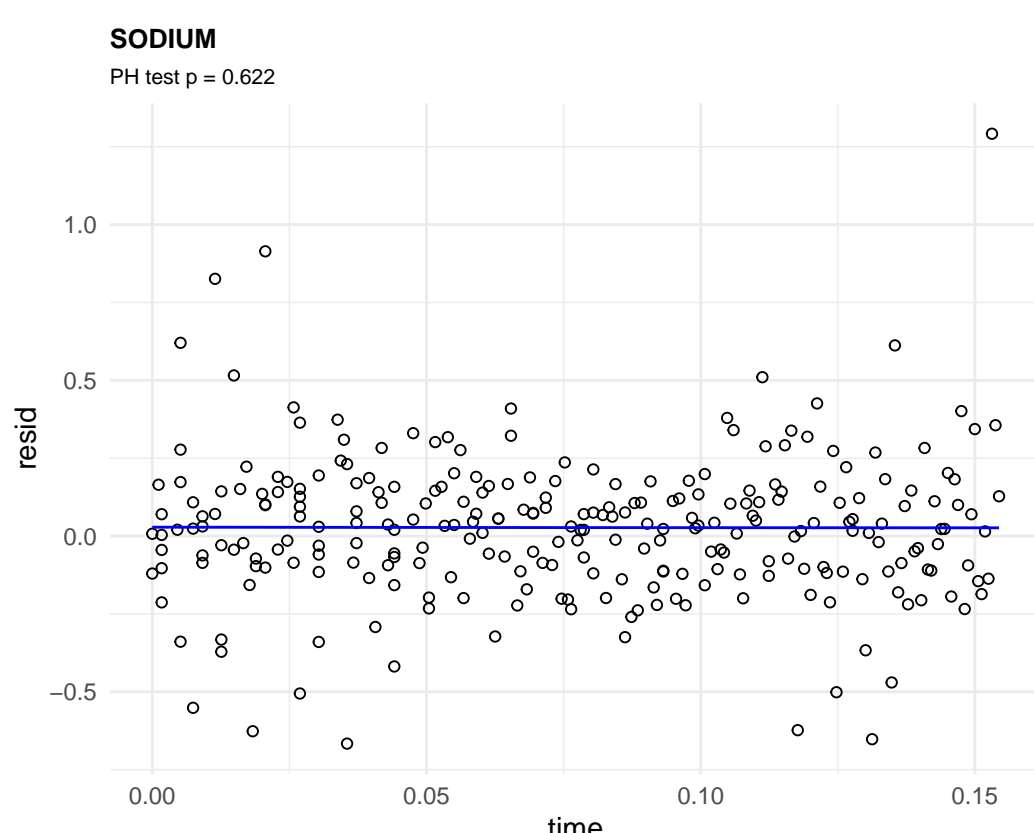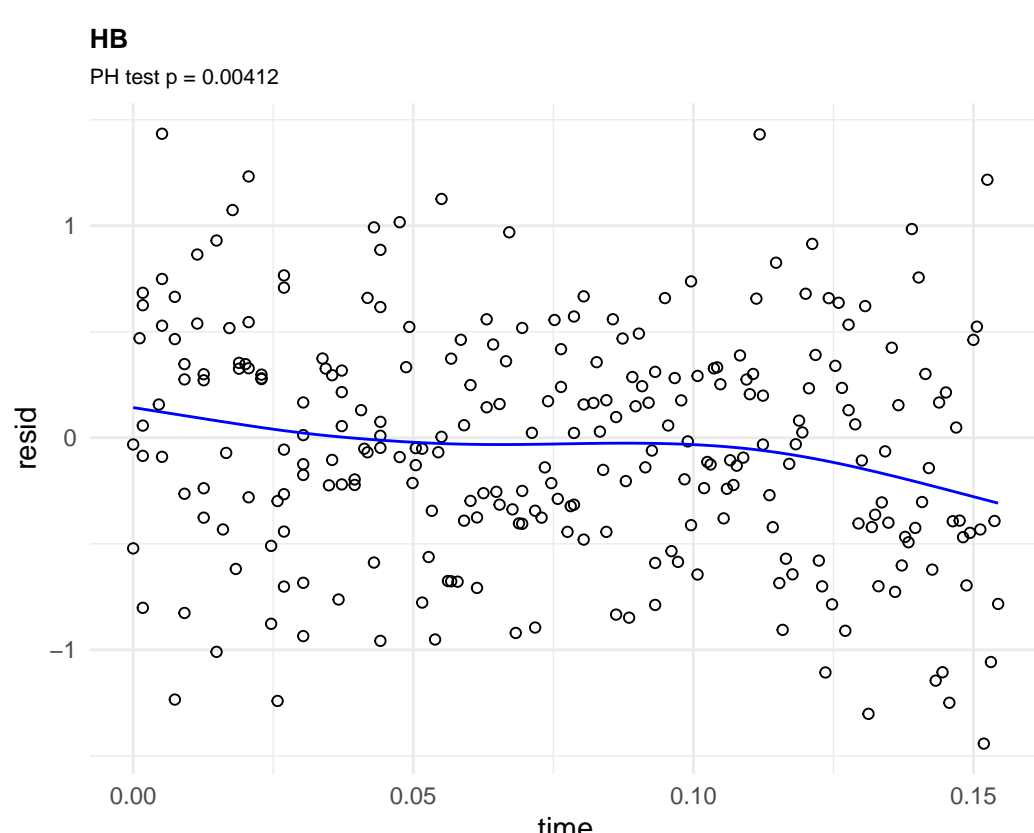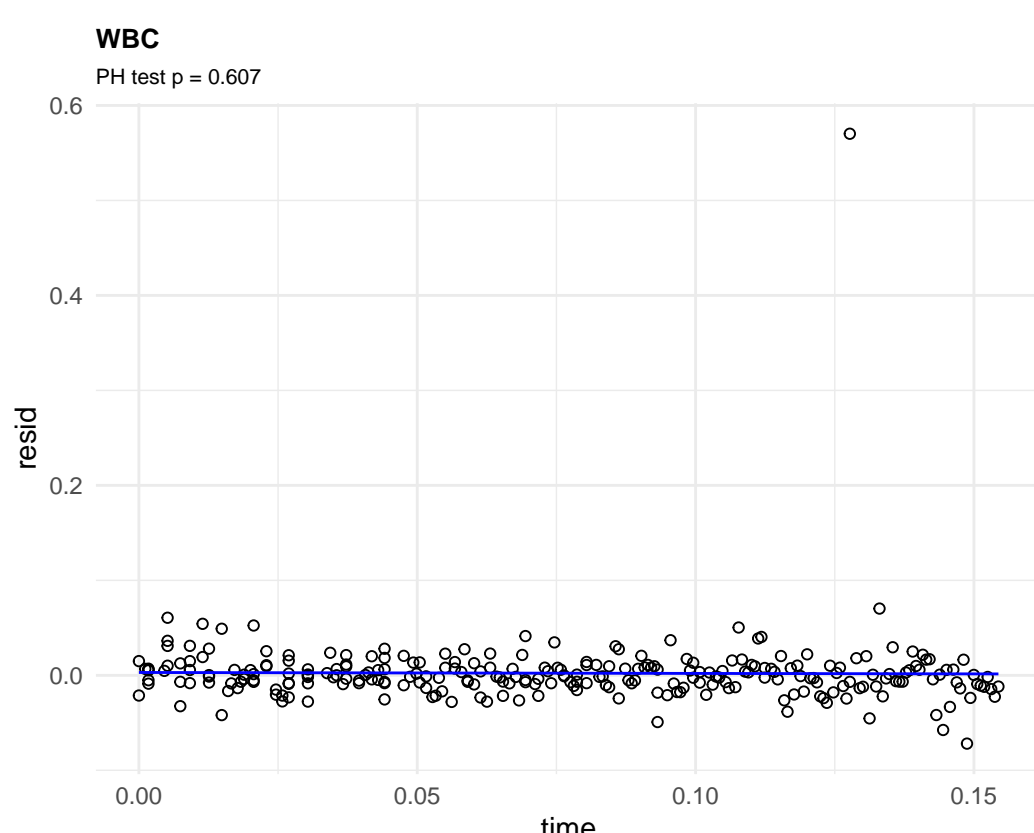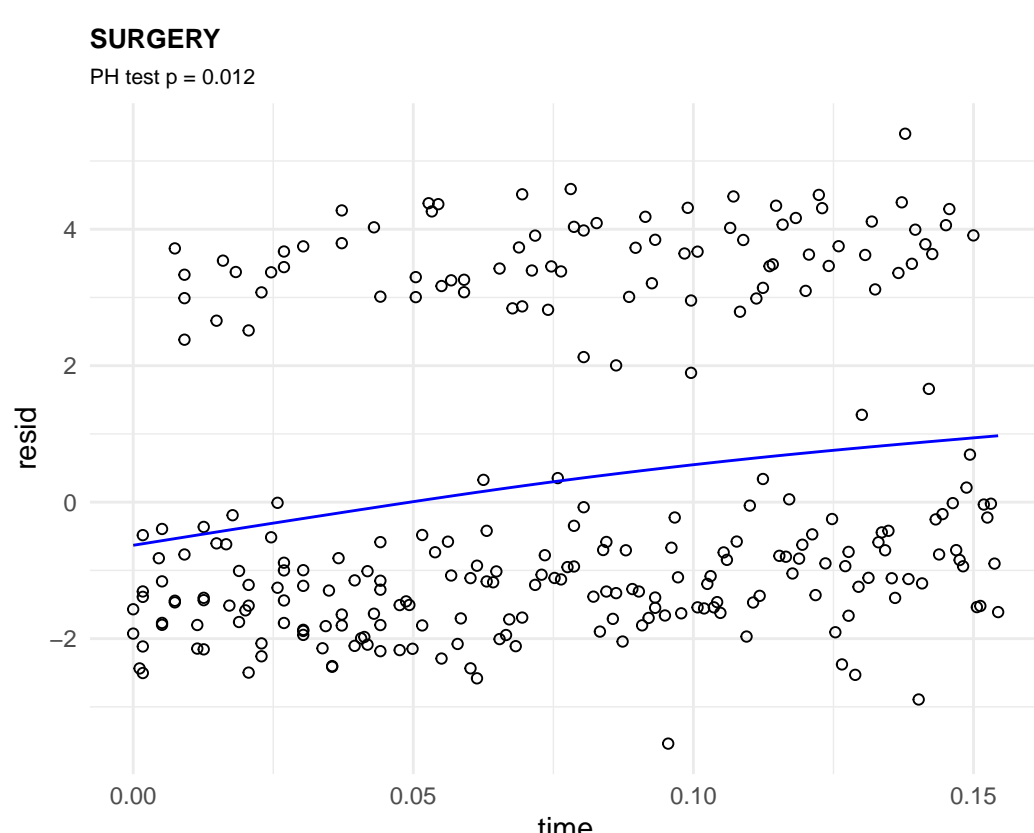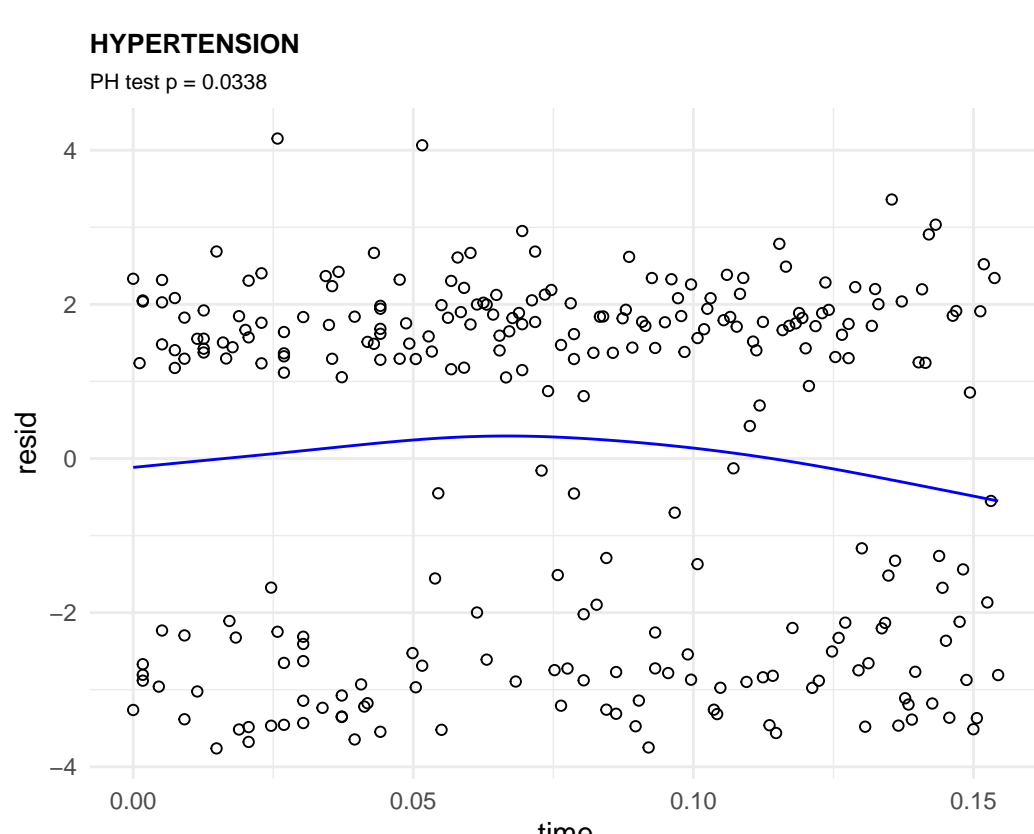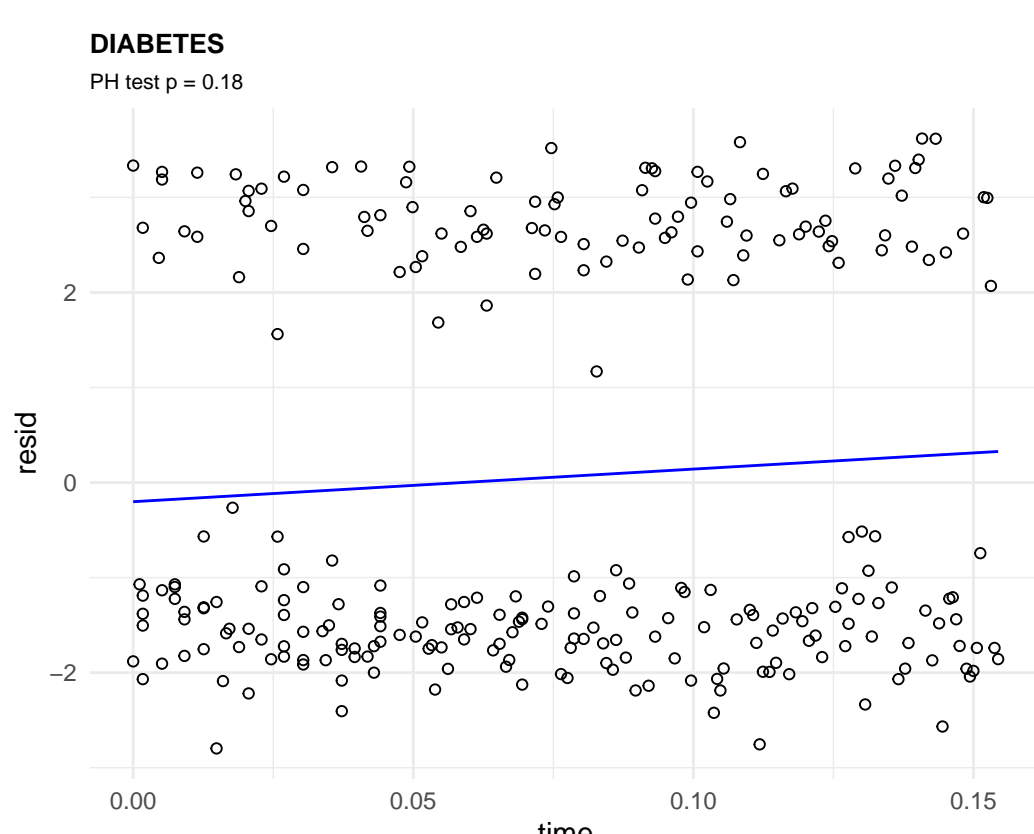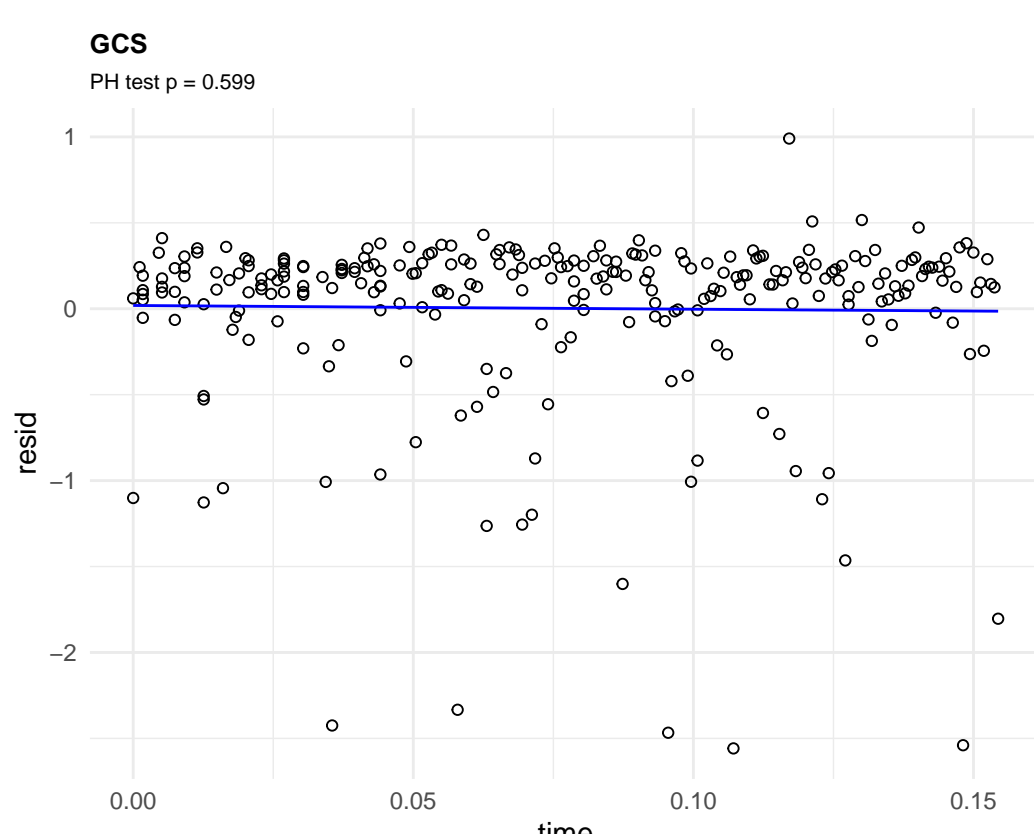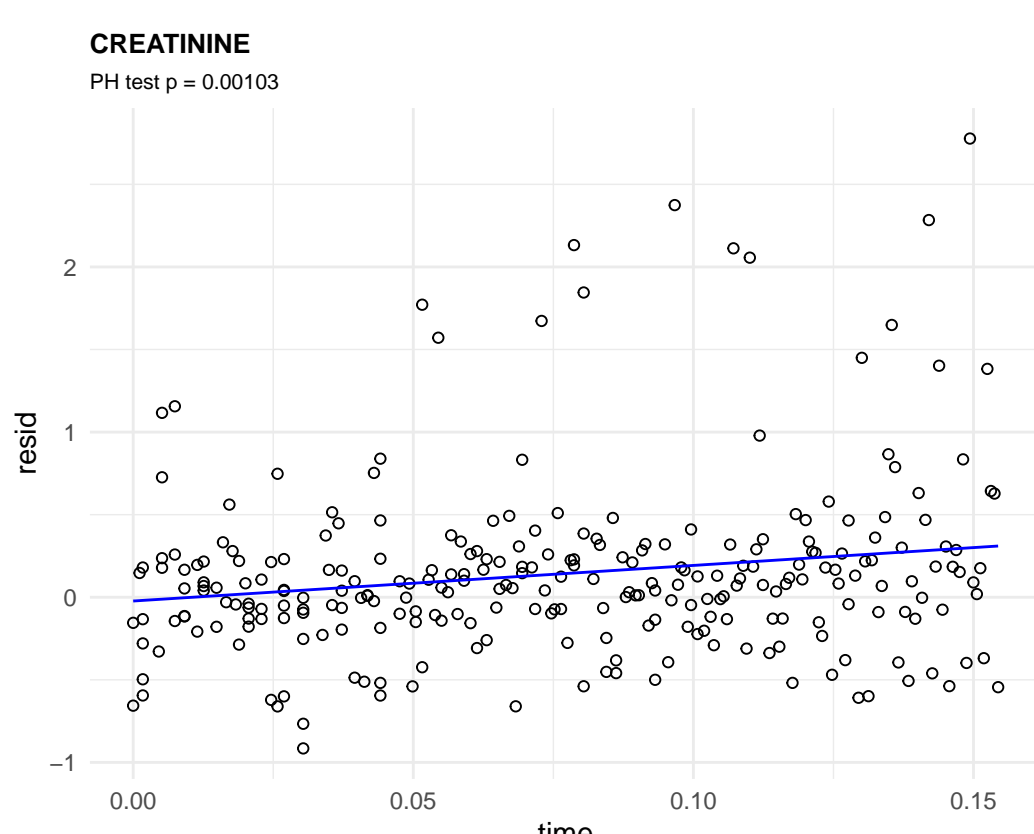

Supplement: Supplementary Figure 2 — Schoenfeld residuals for in-hospital mortality model. [file DataSheet2.pdf]

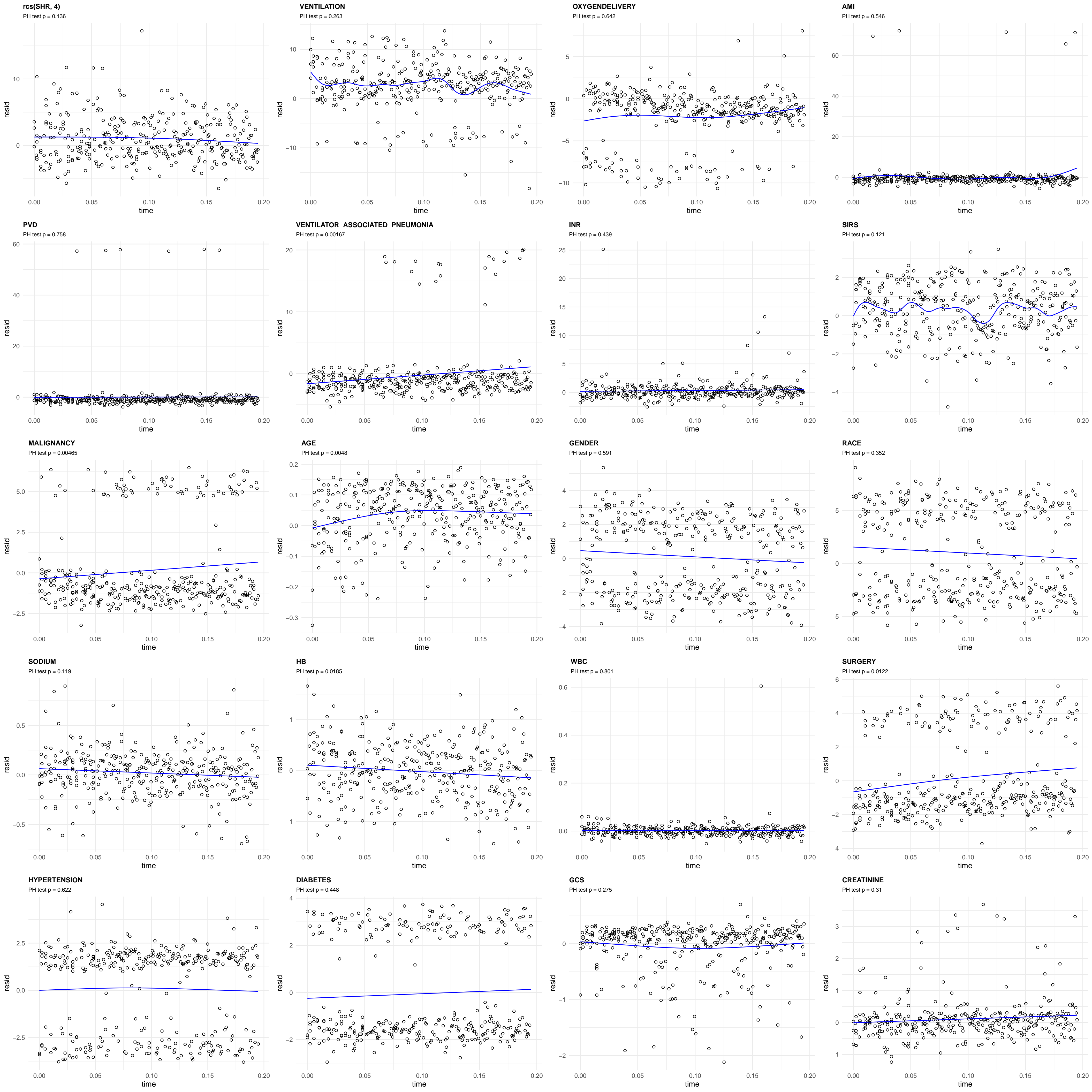

Supplement: Supplementary Figure 3 — Schoenfeld residuals for 30-day mortality model. [file DataSheet3.pdf]

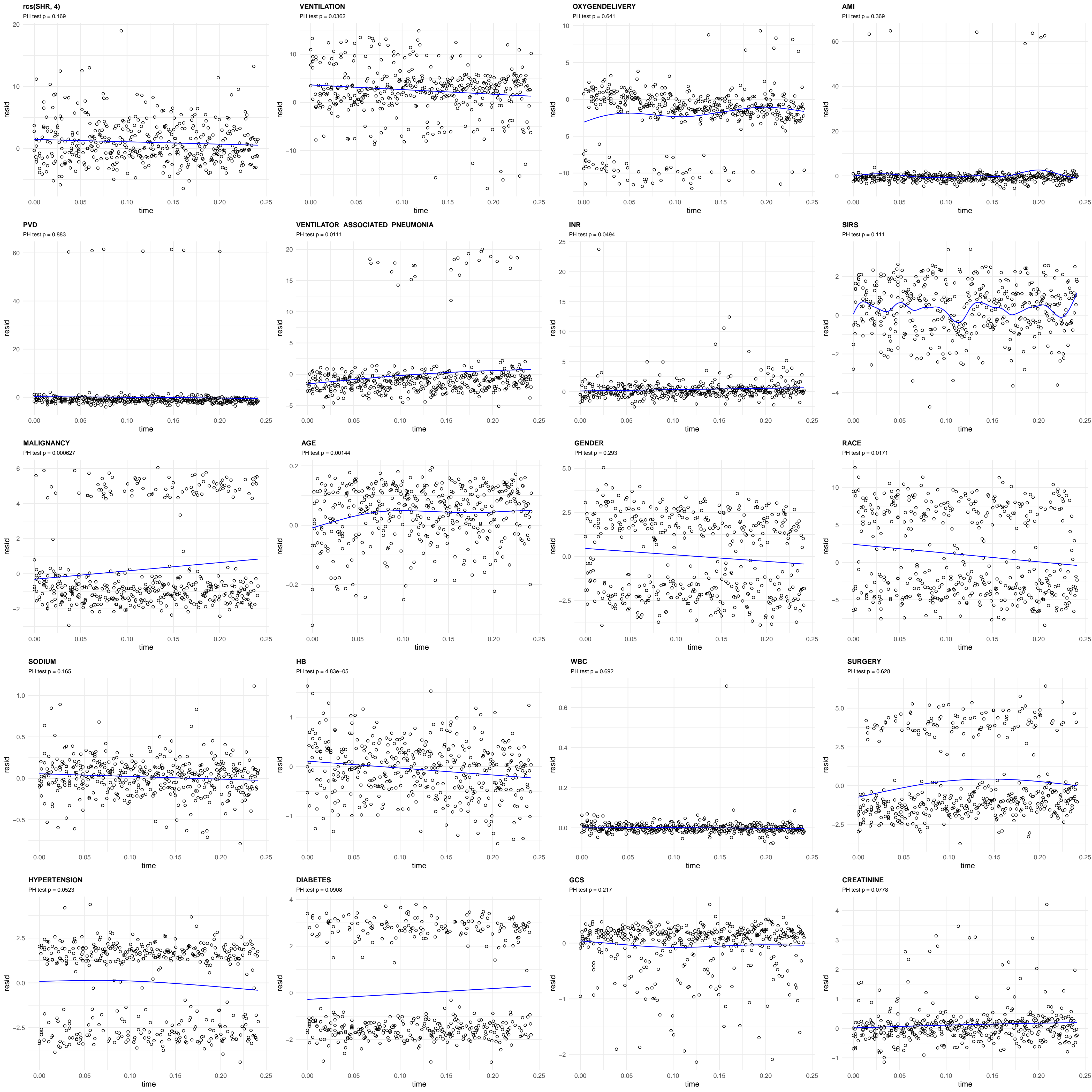

Supplement: Supplementary Figure 4 — Schoenfeld residuals for 90-day mortality model. [file DataSheet4.pdf]

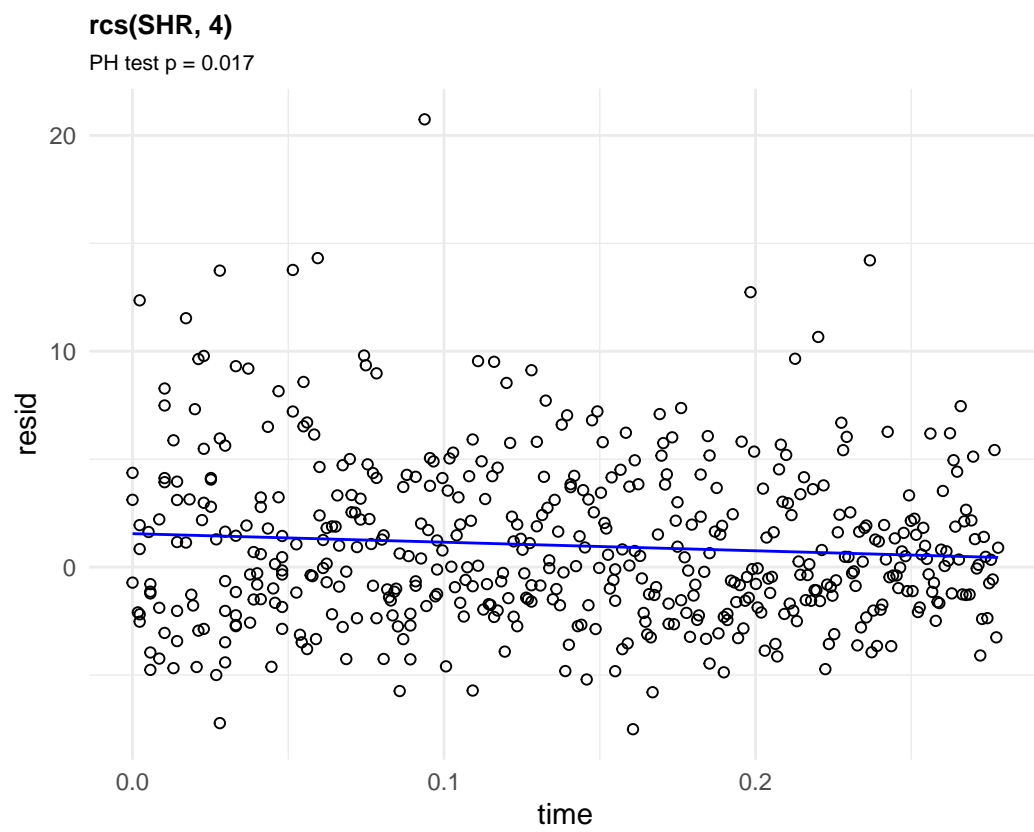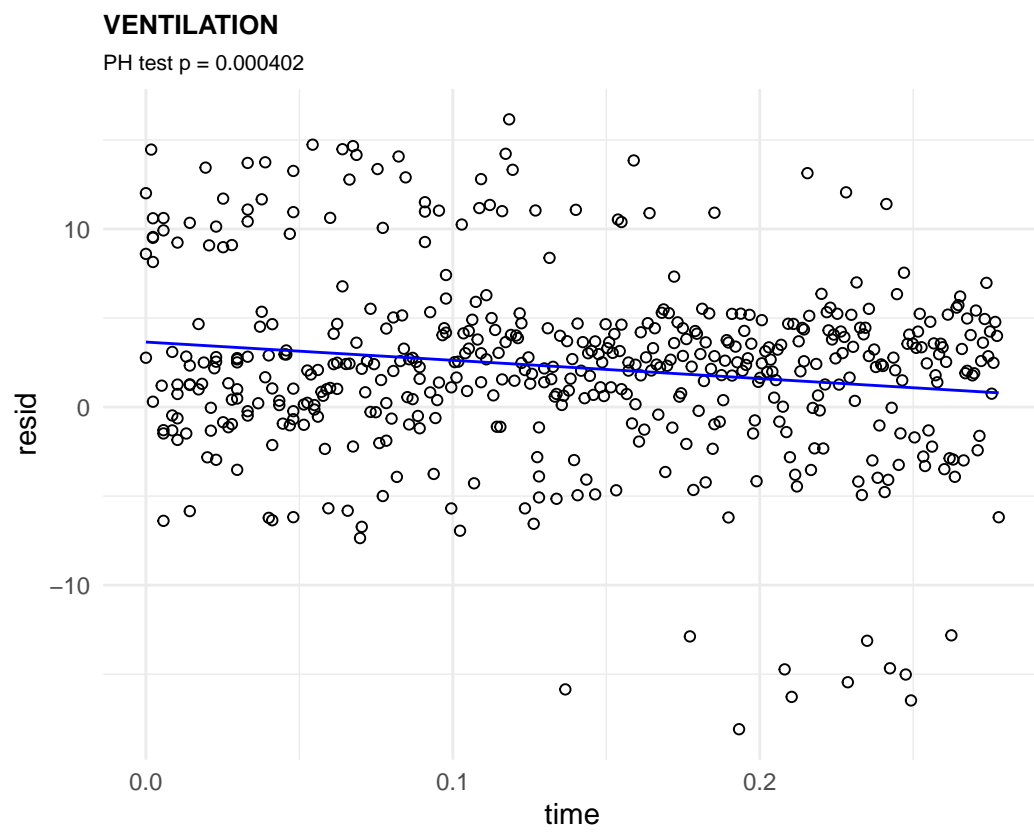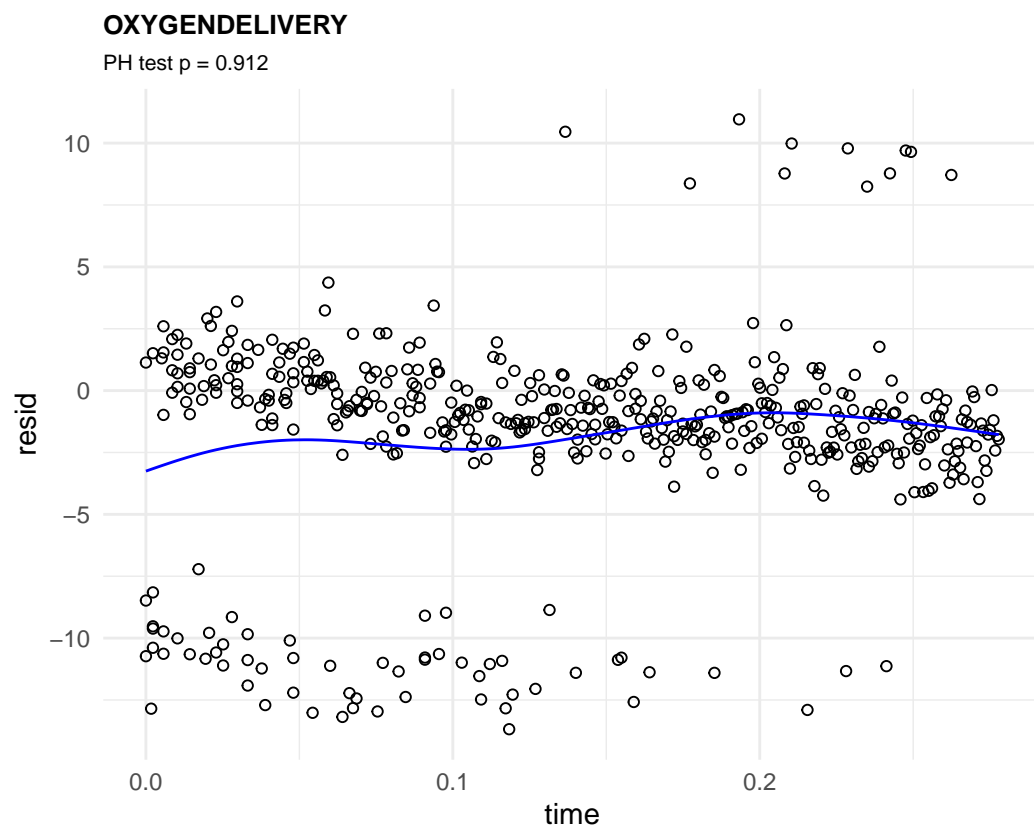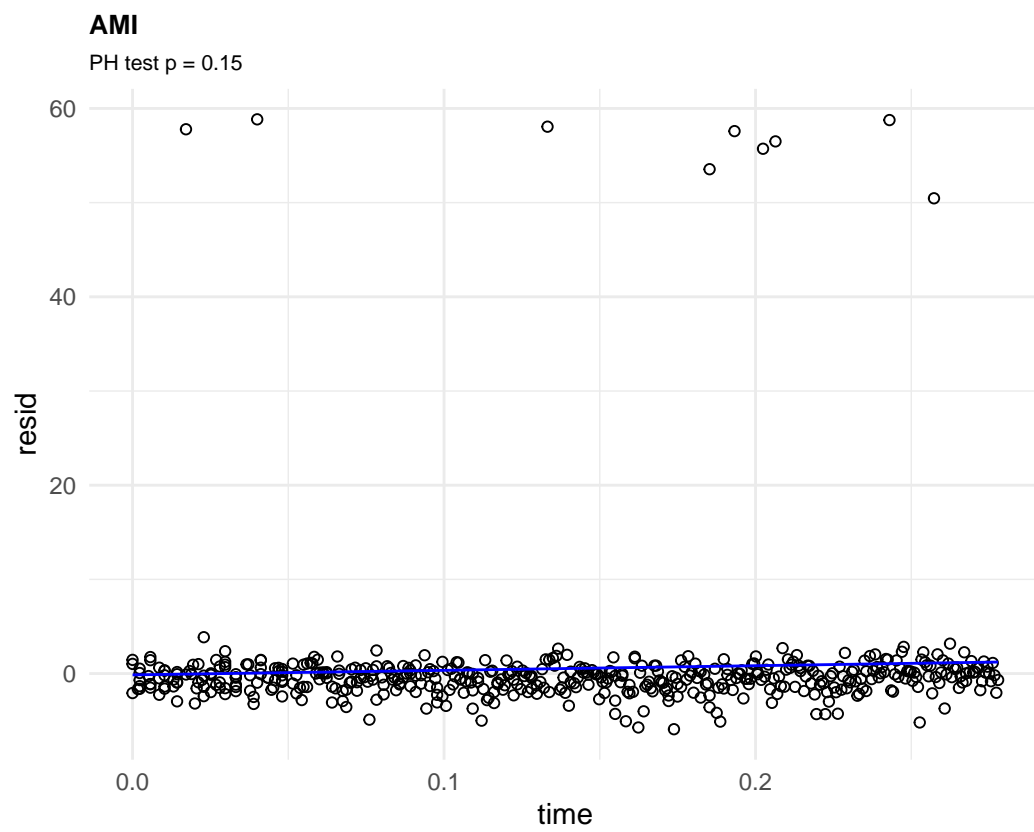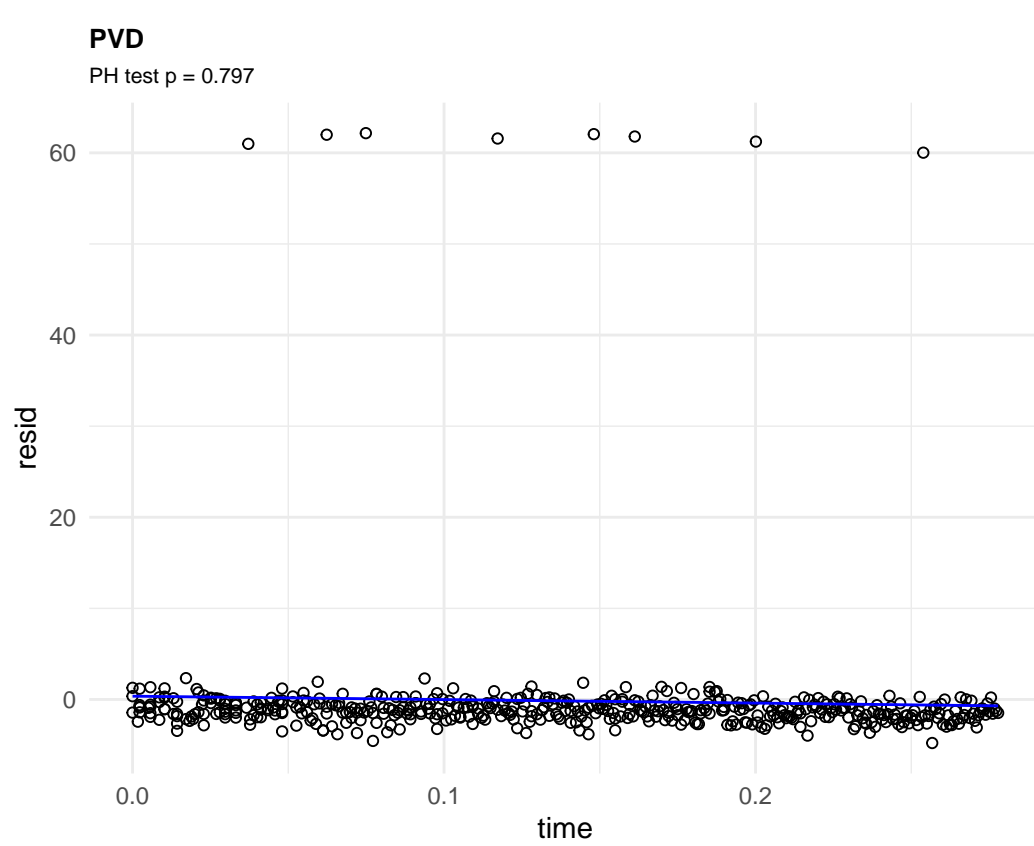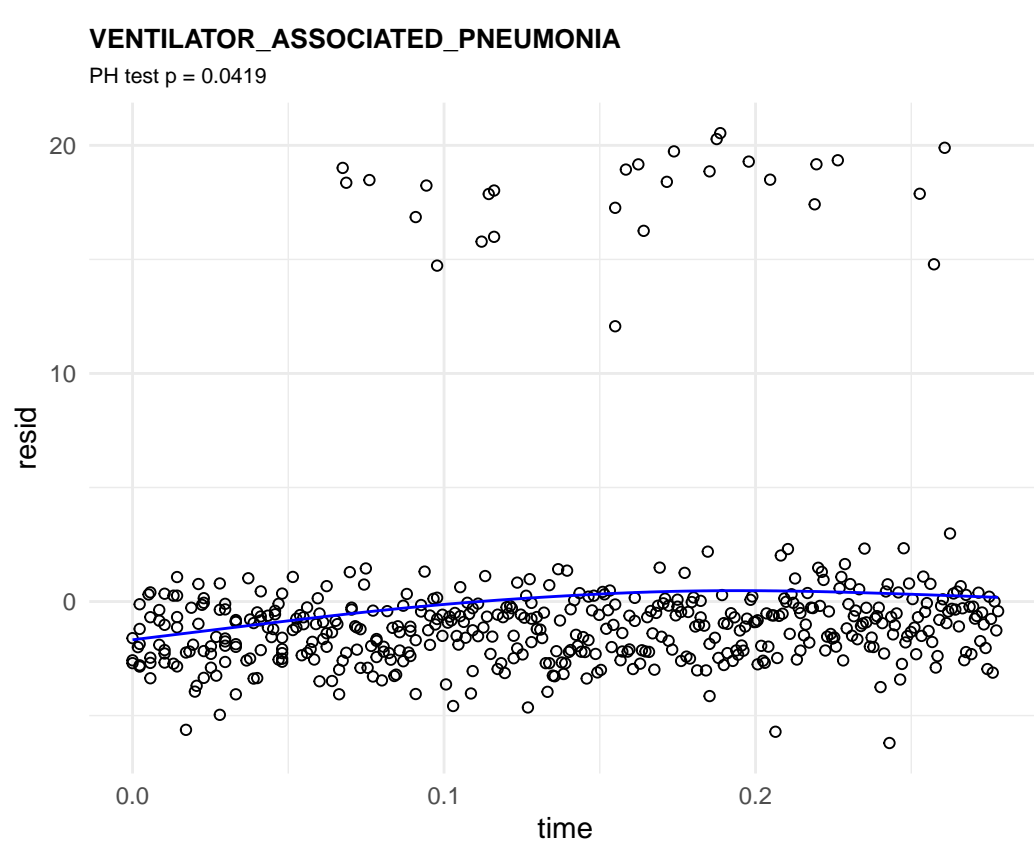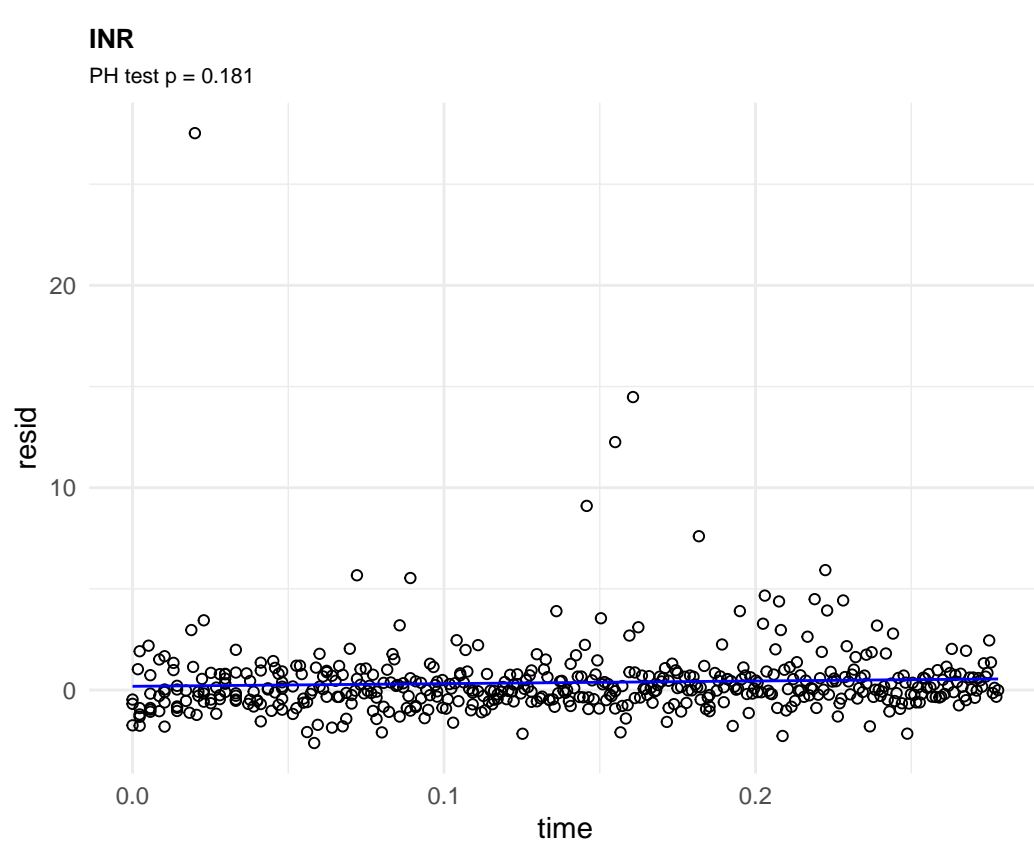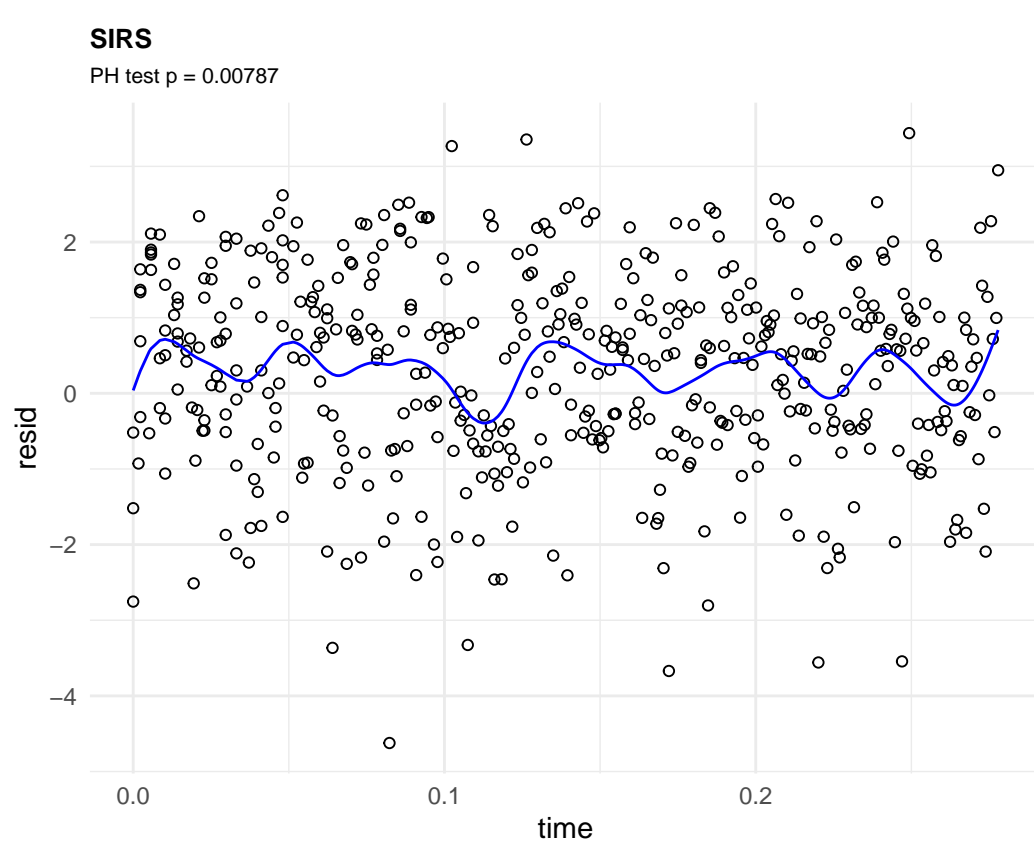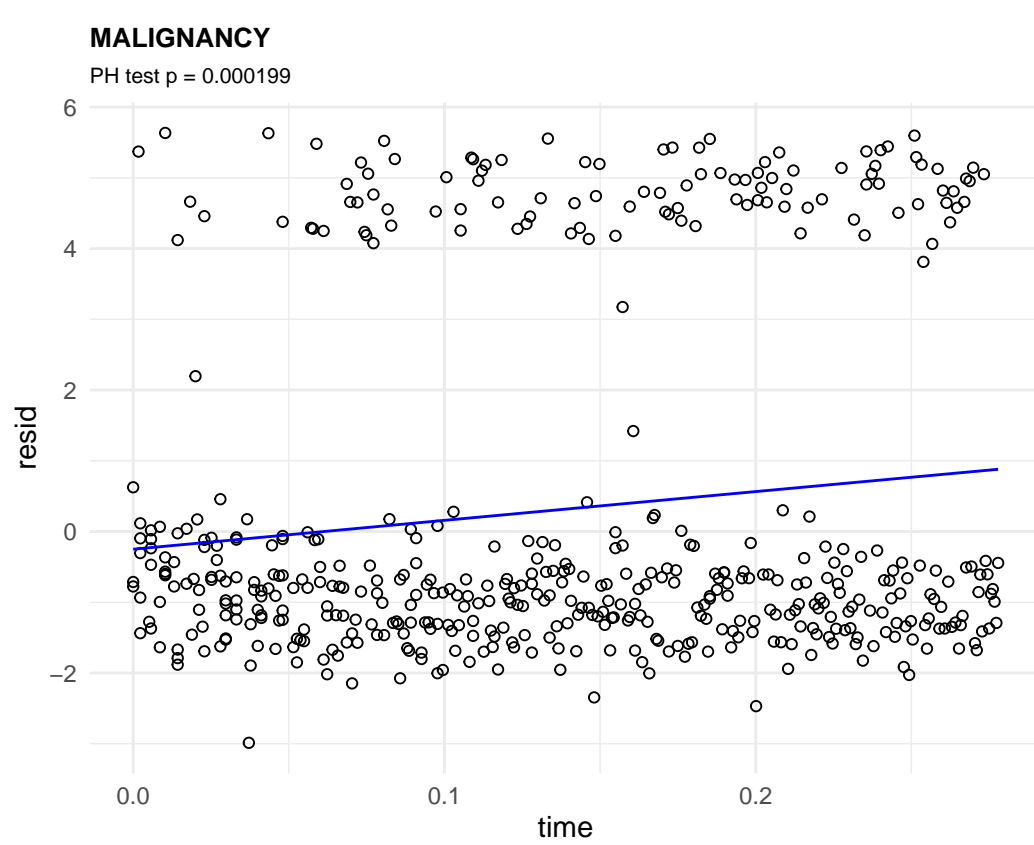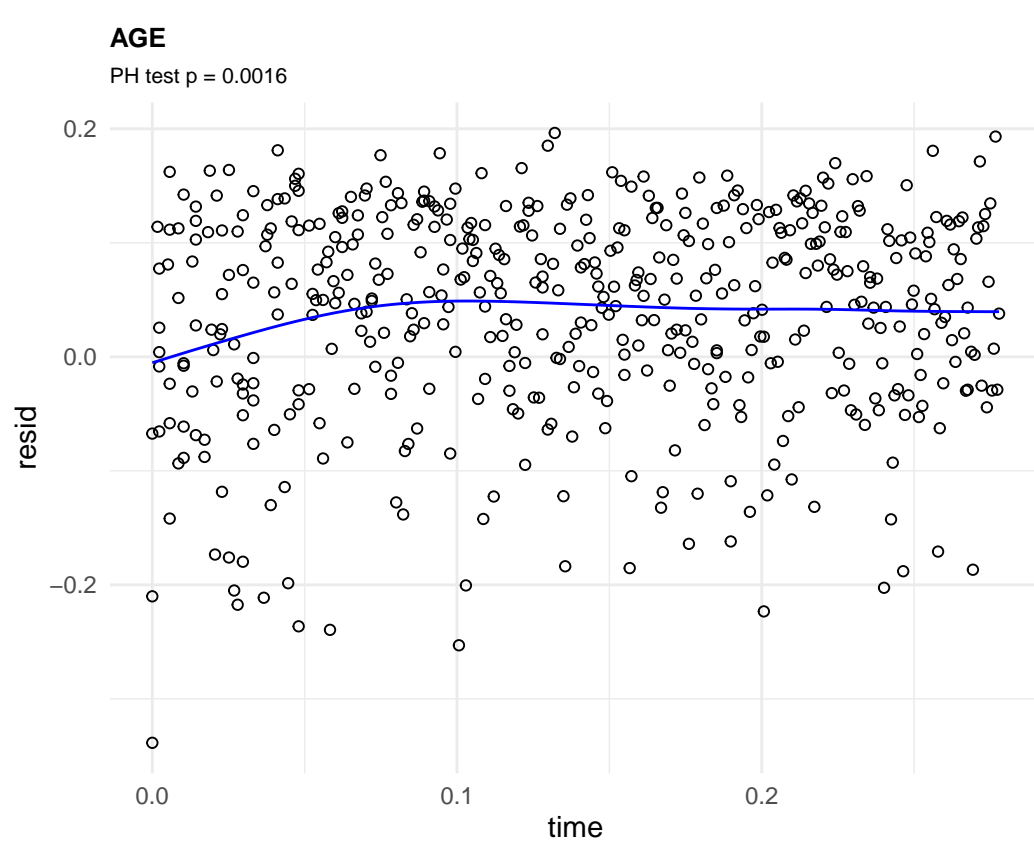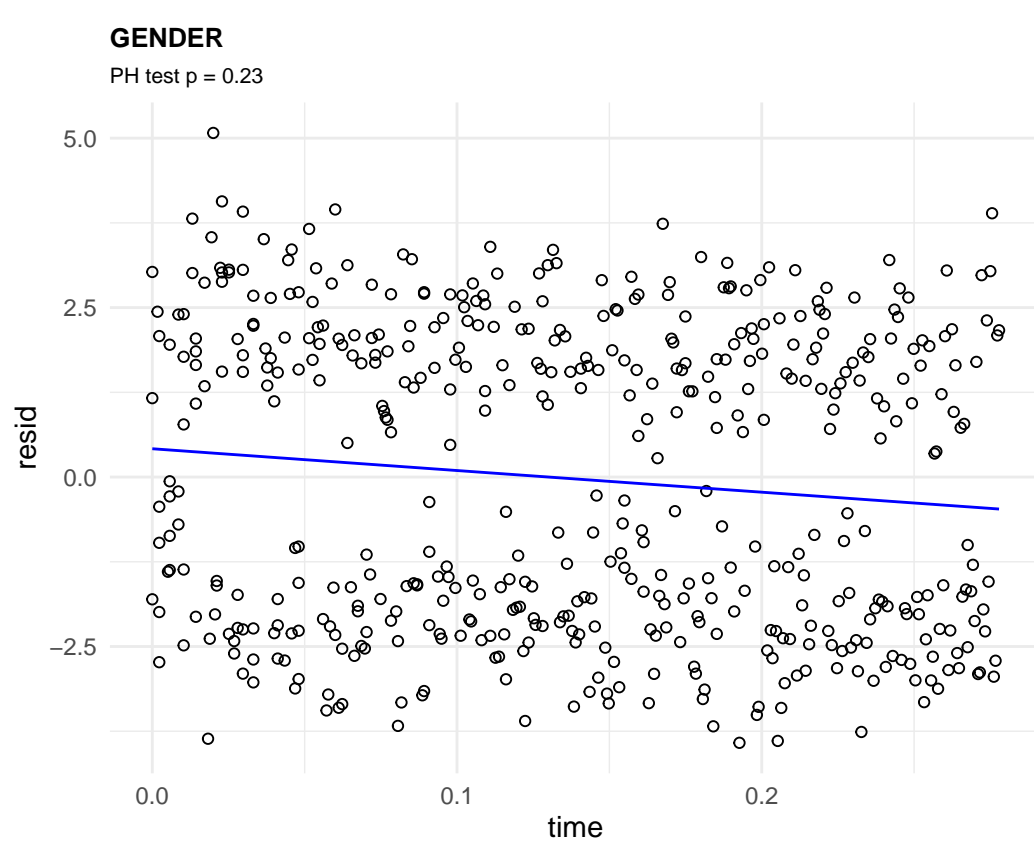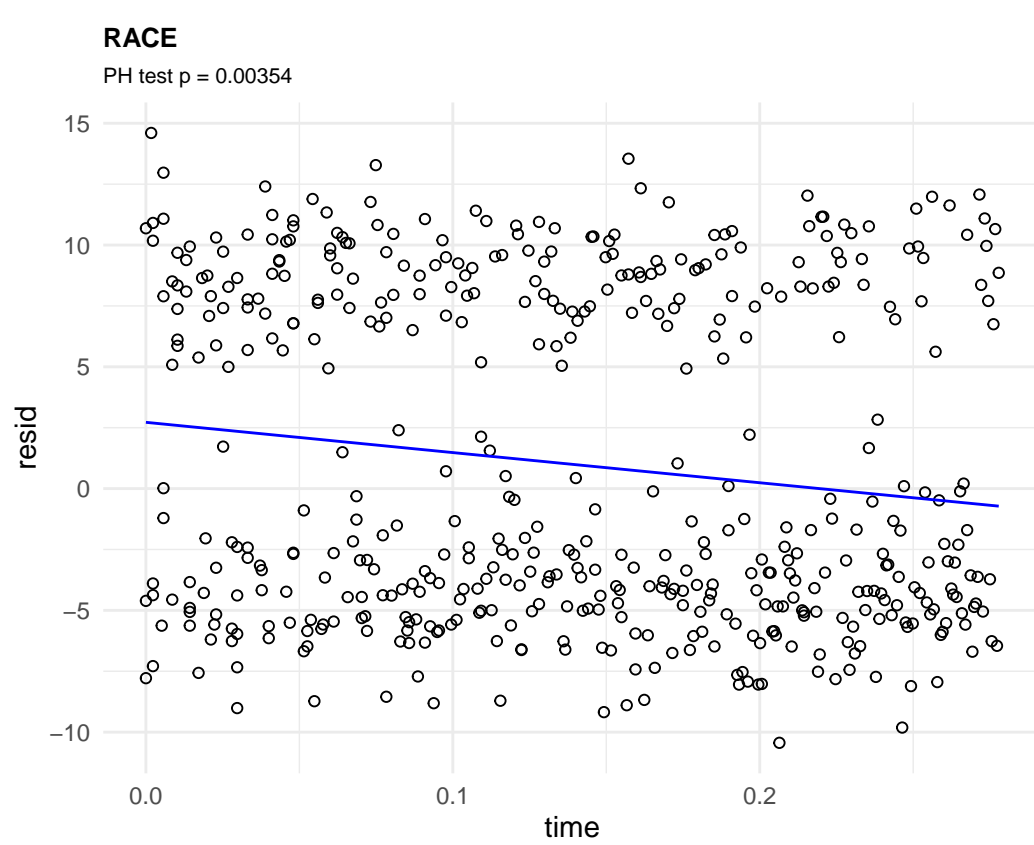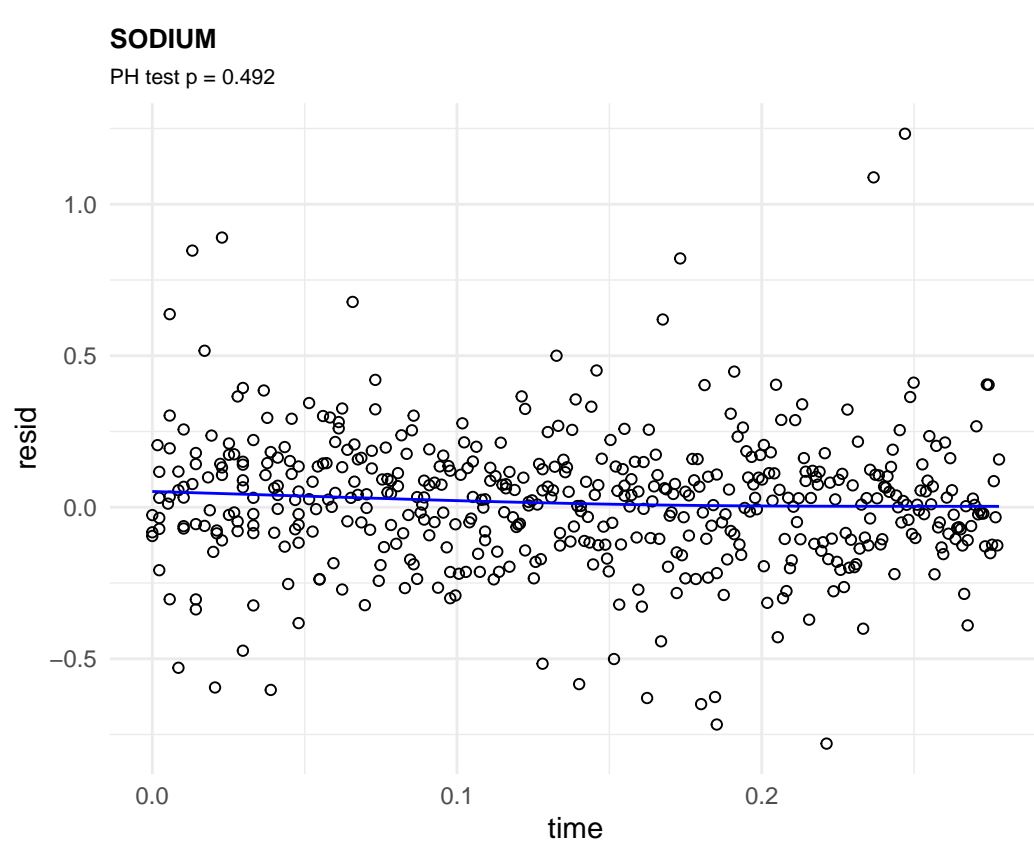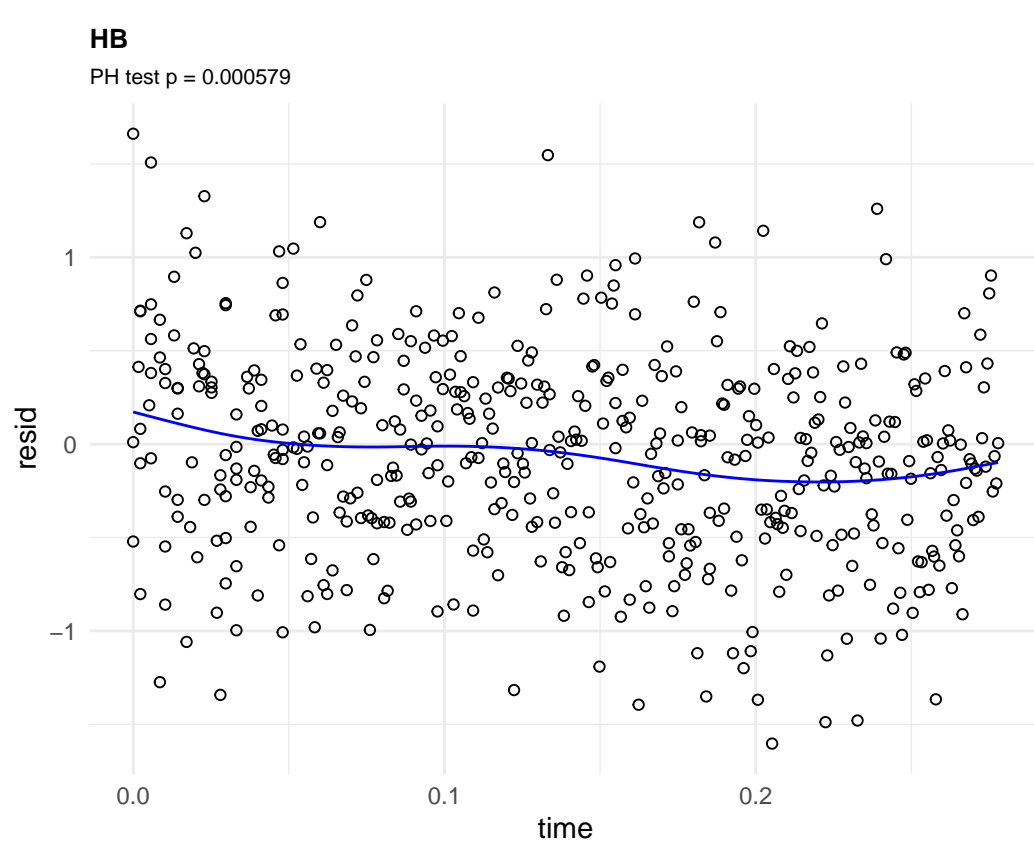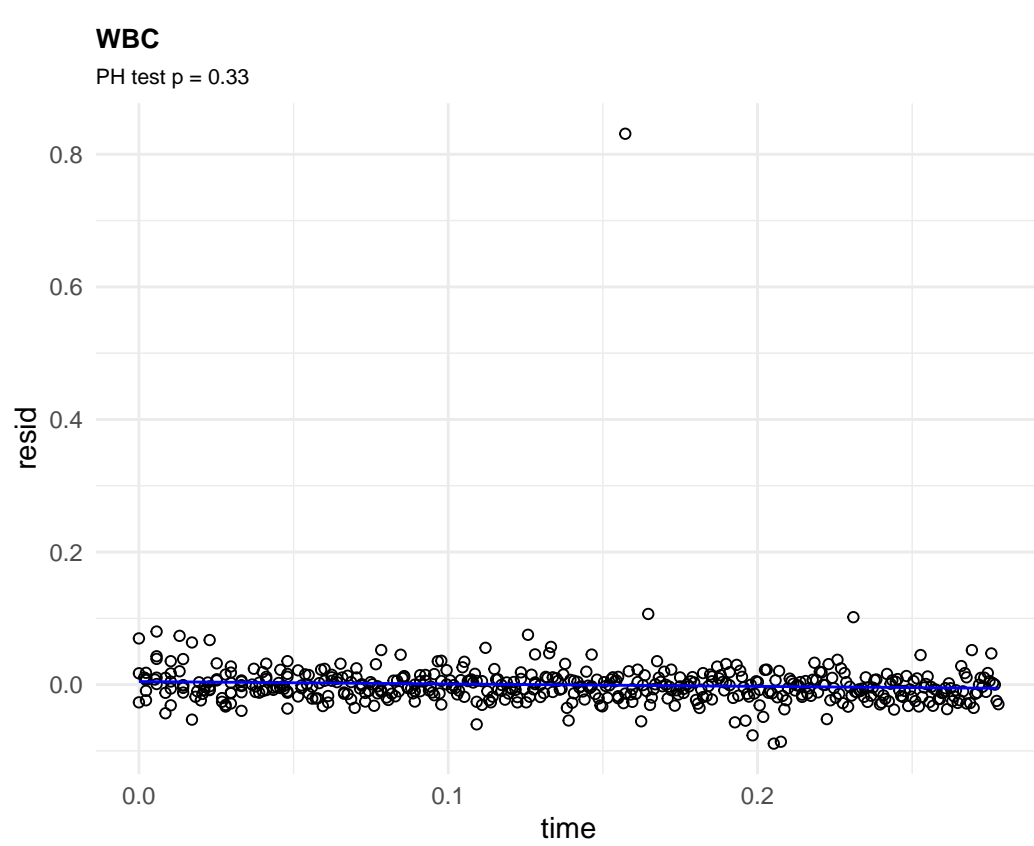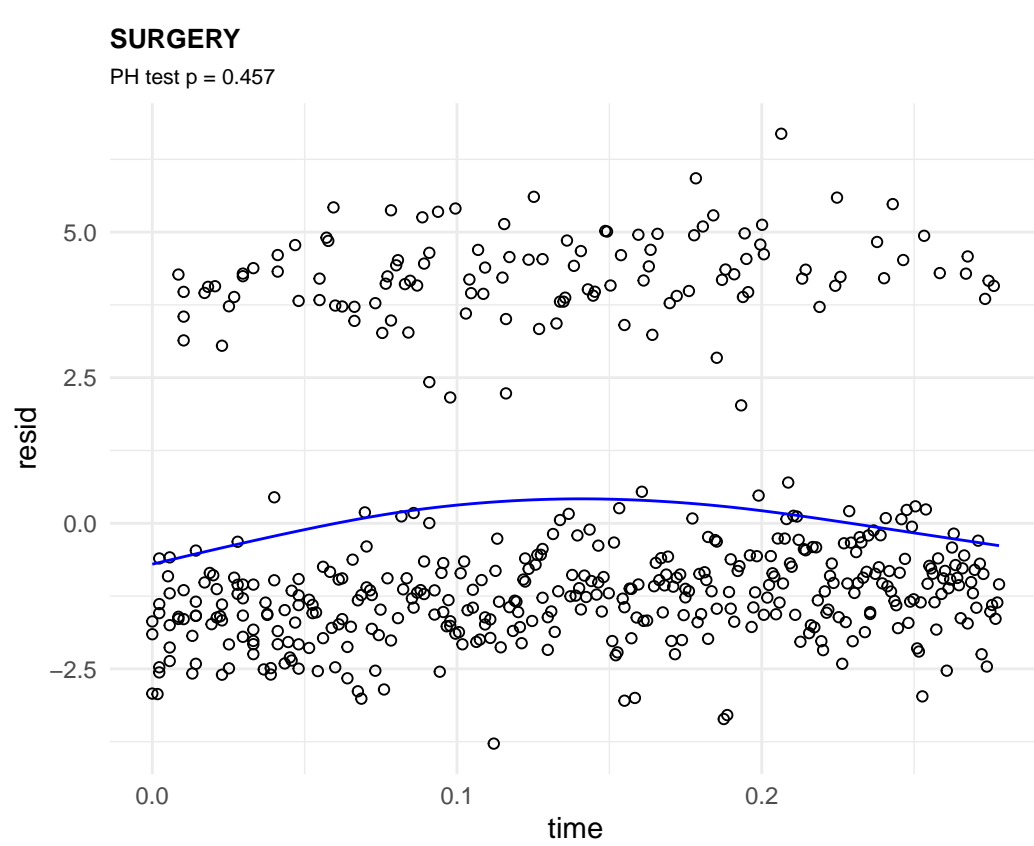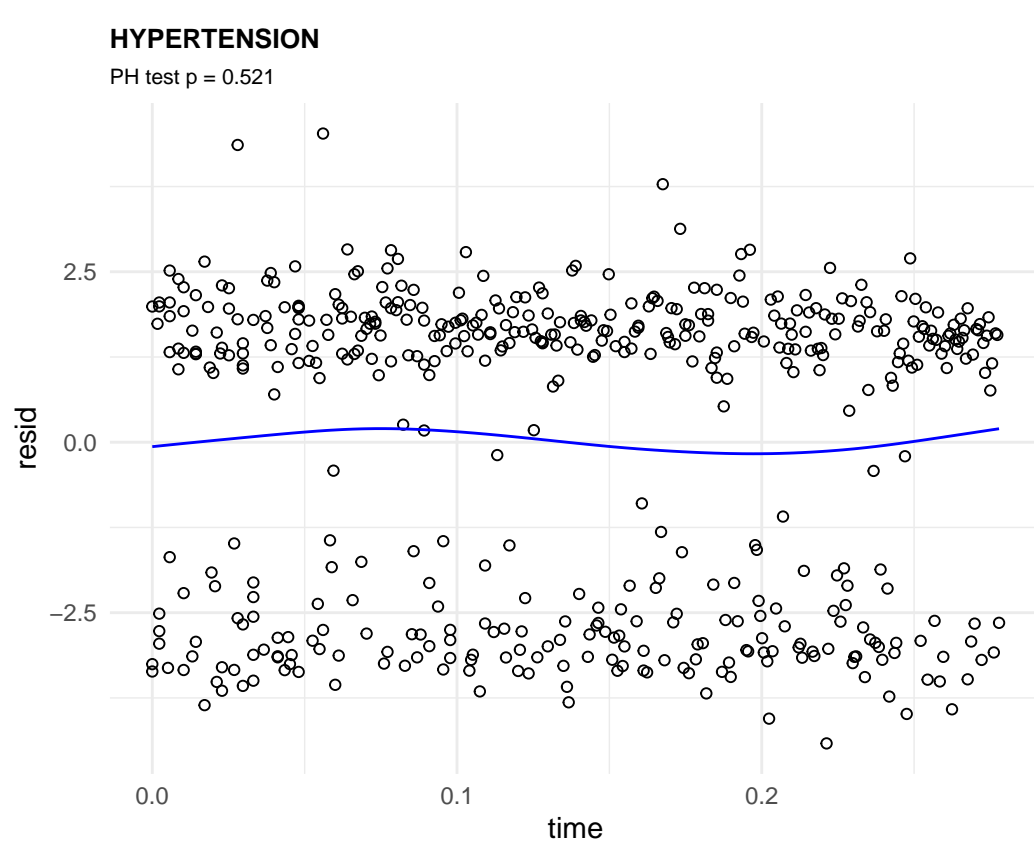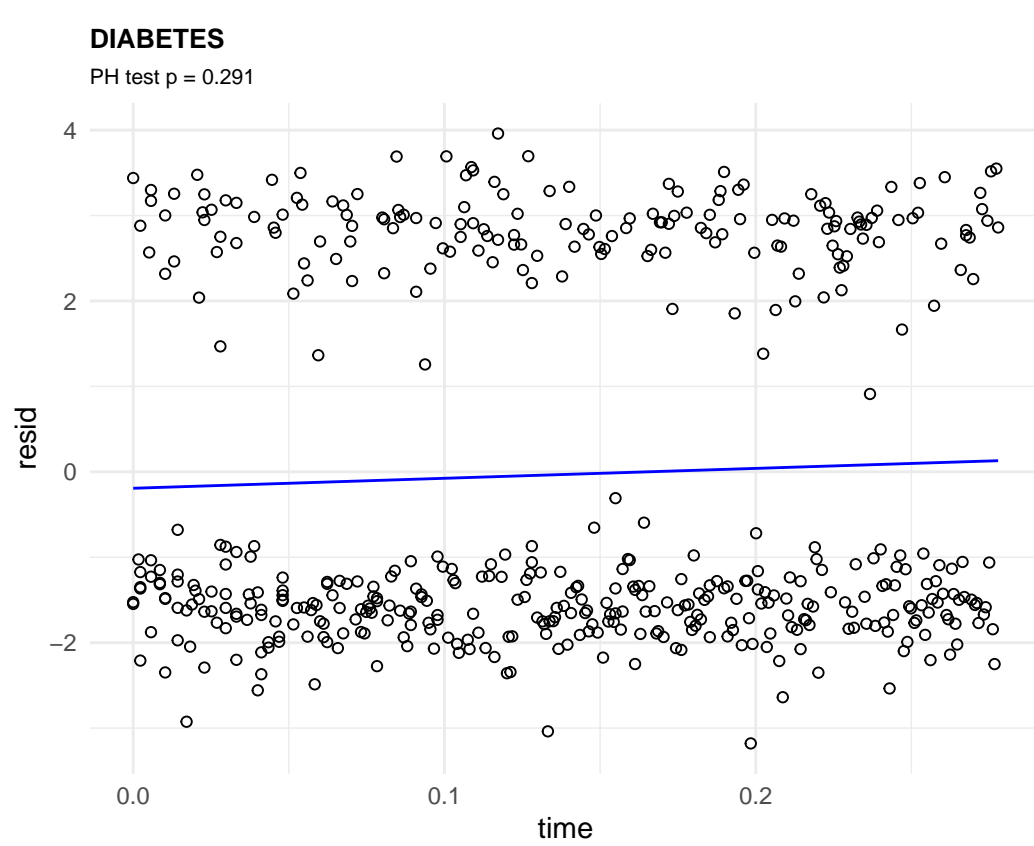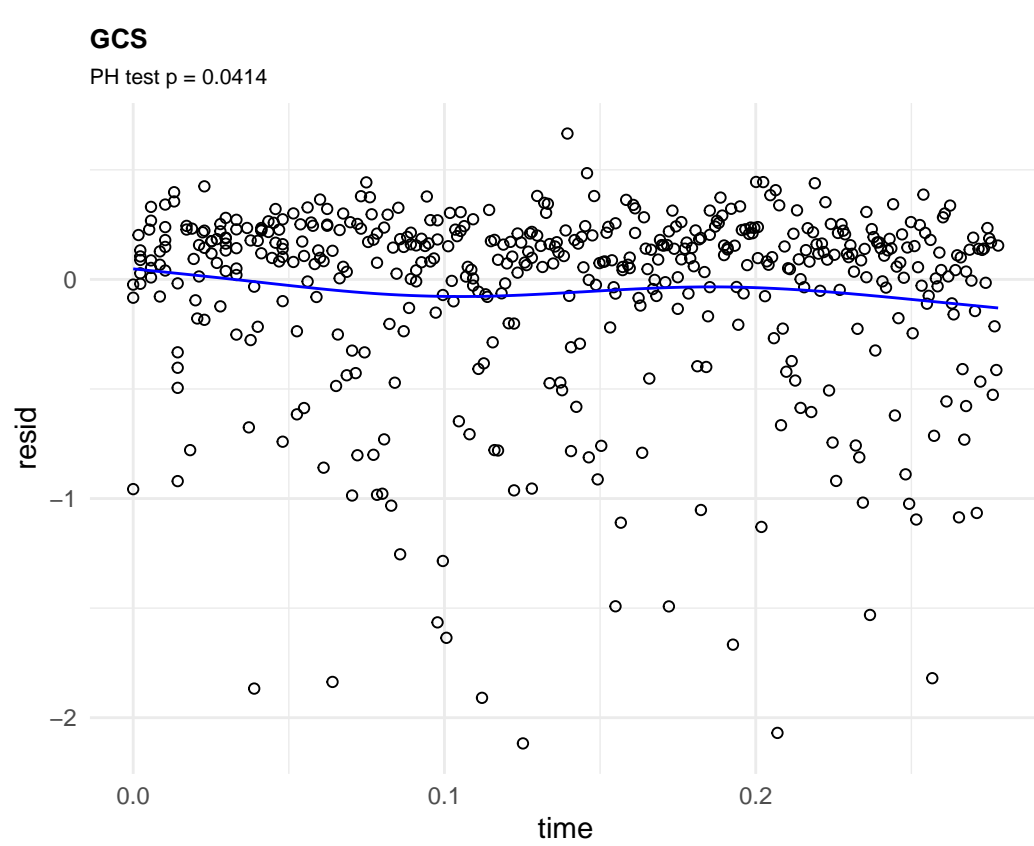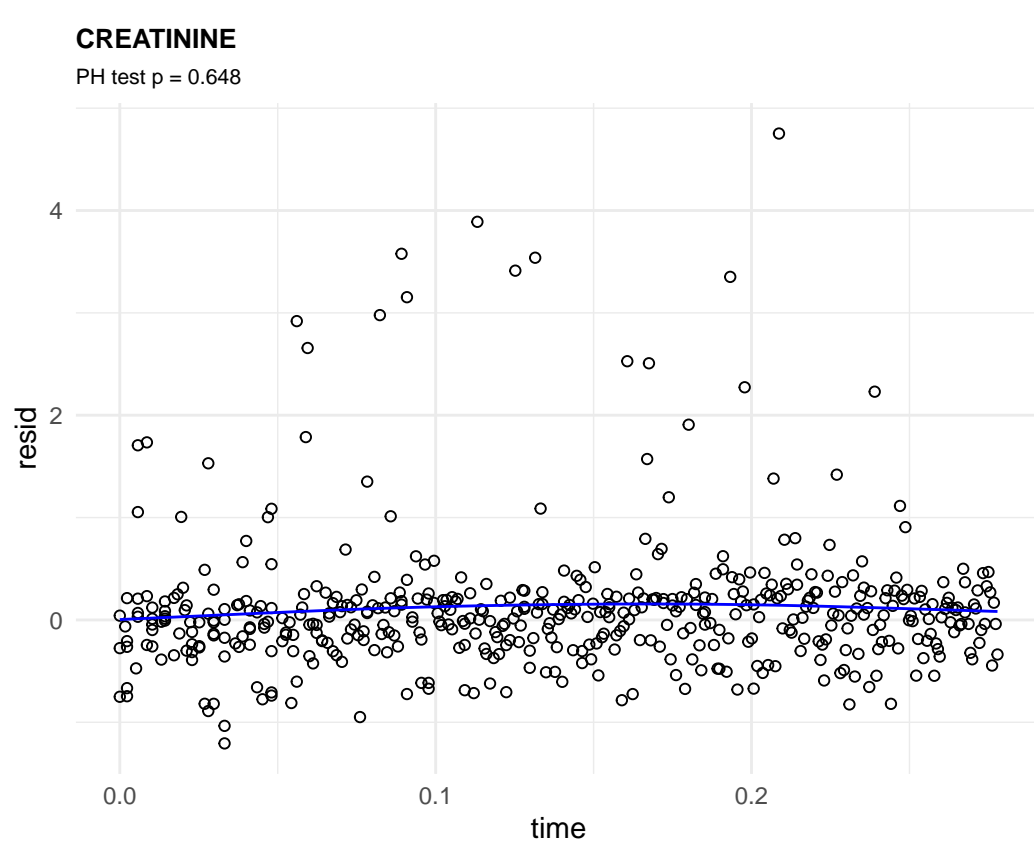

Supplement: Supplementary Figure 5 — Schoenfeld residuals for 180-day mortality model. [file DataSheet5.pdf]

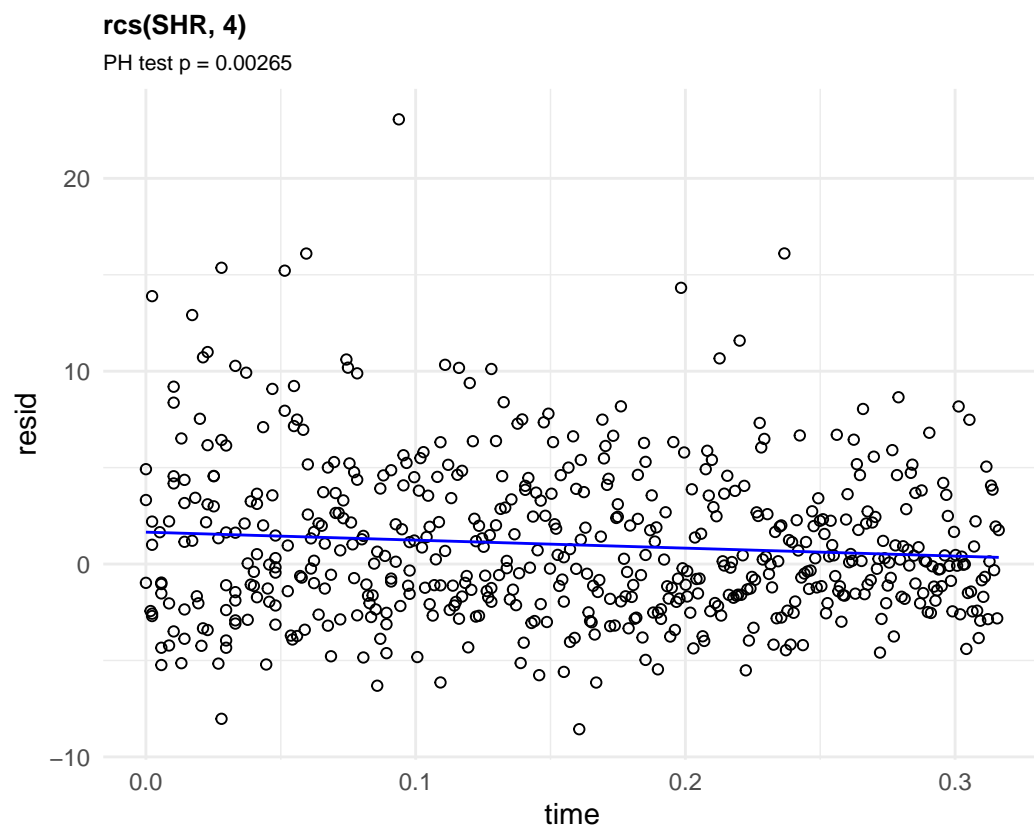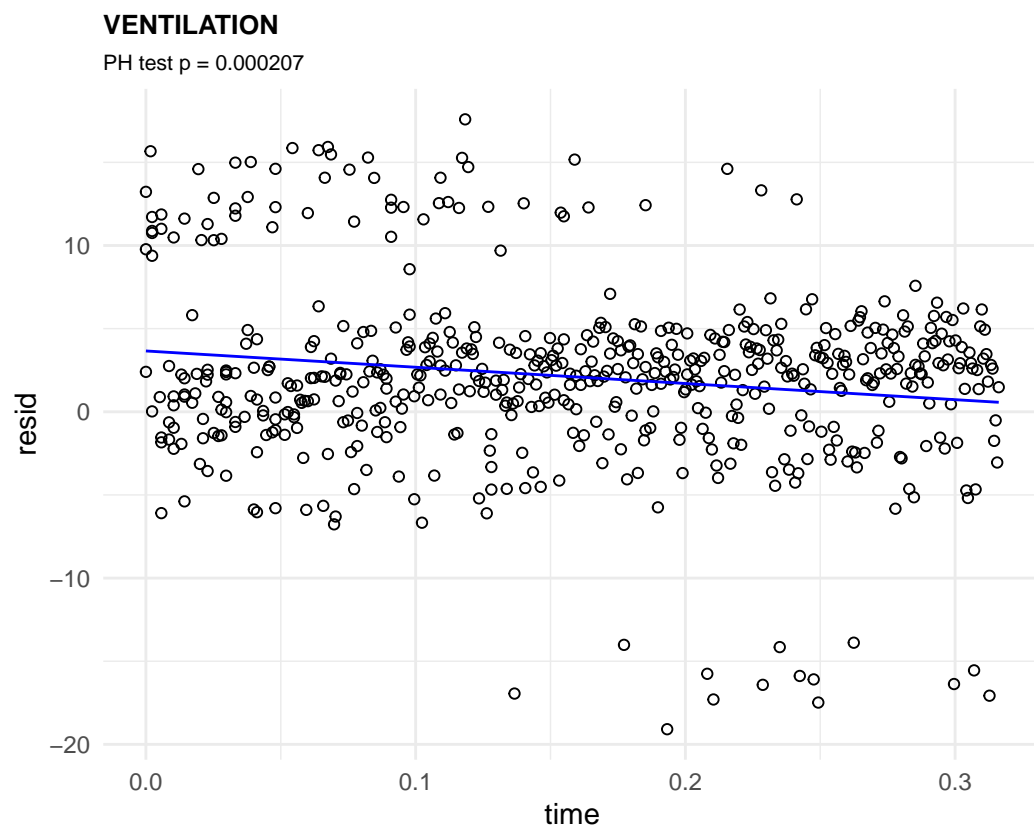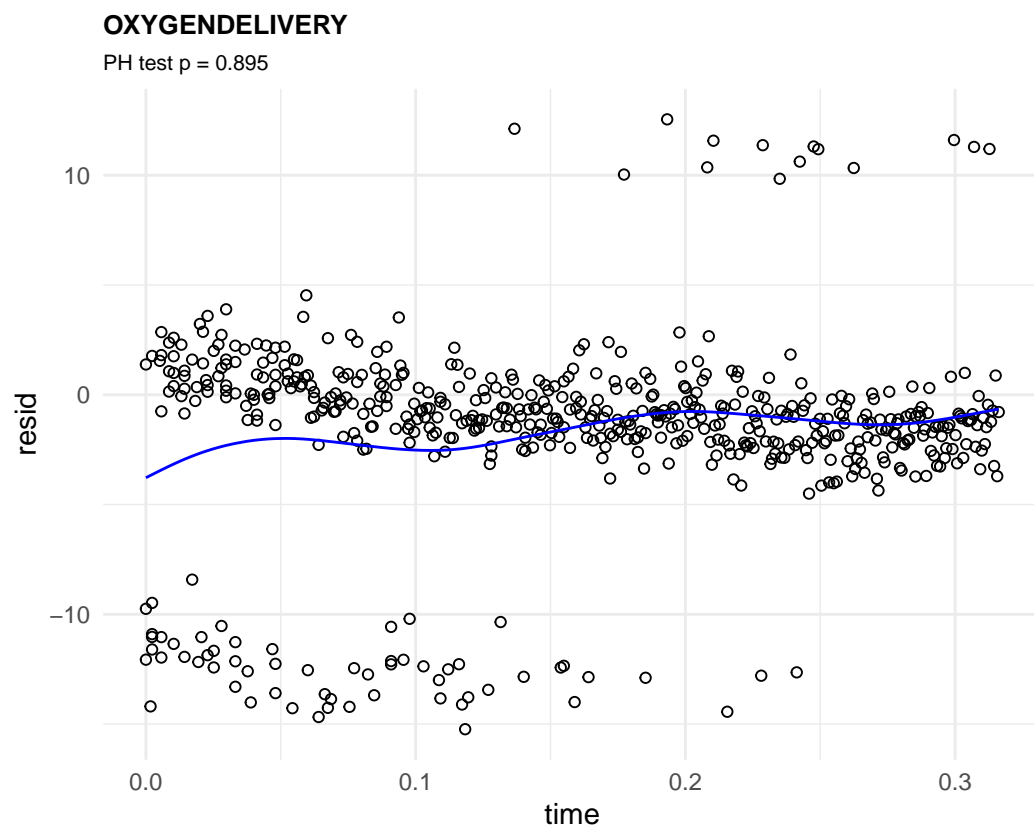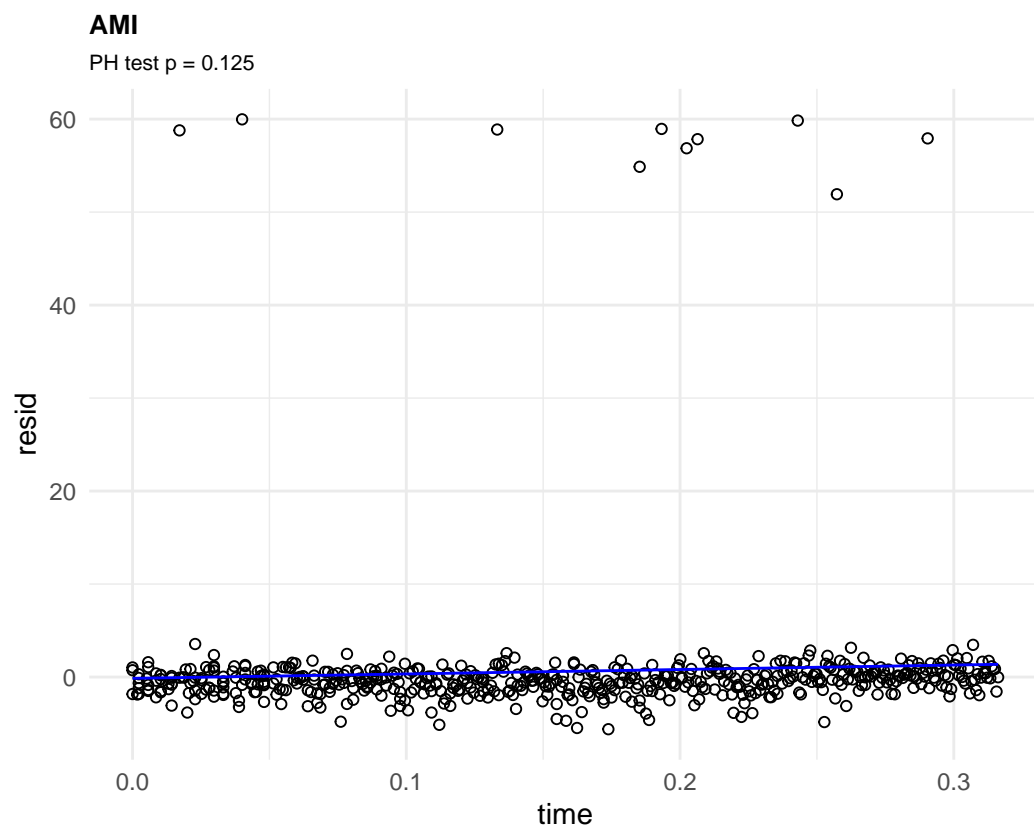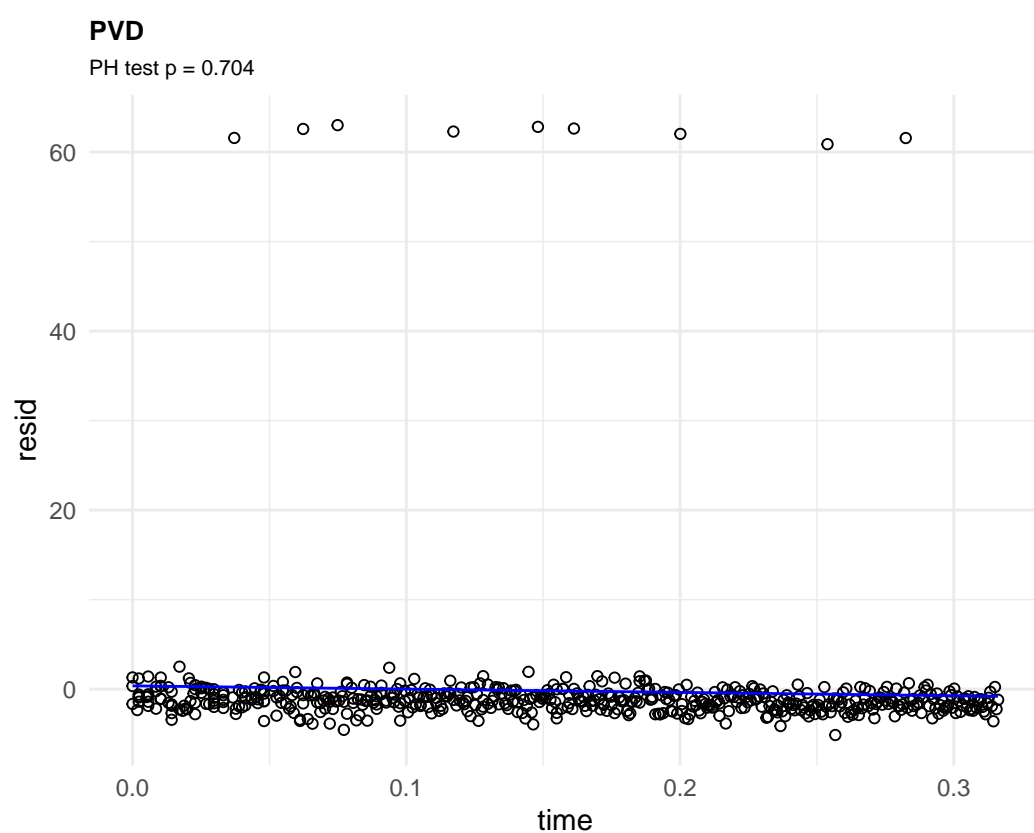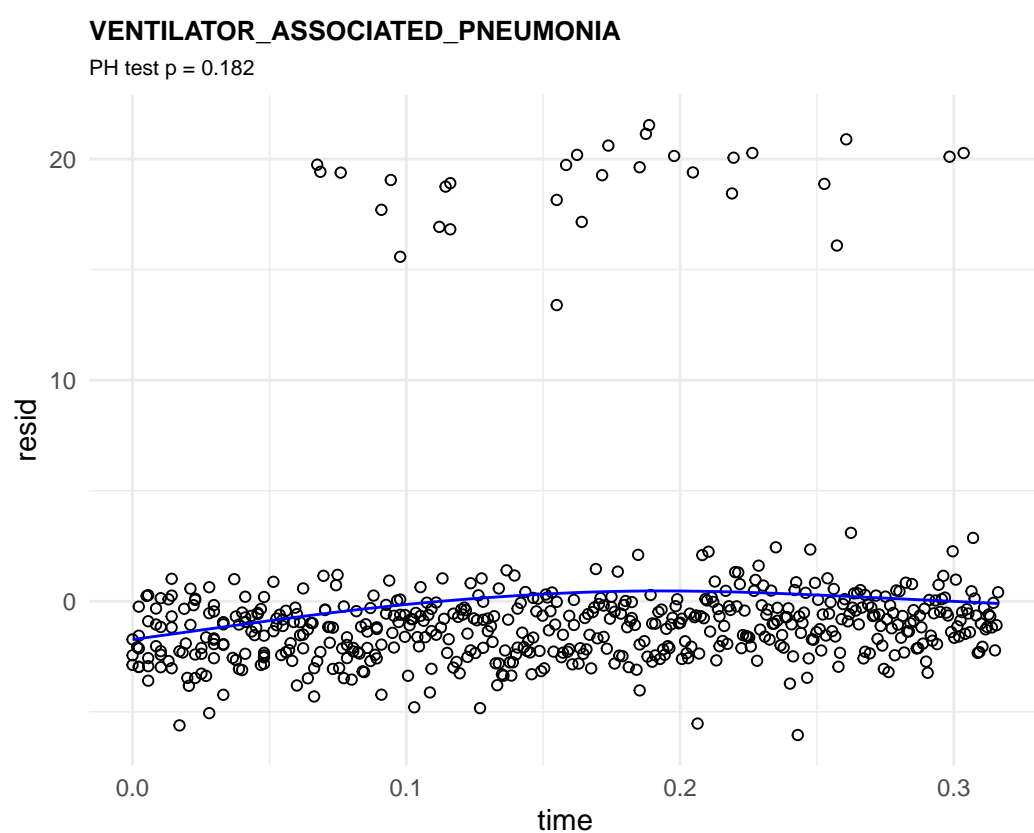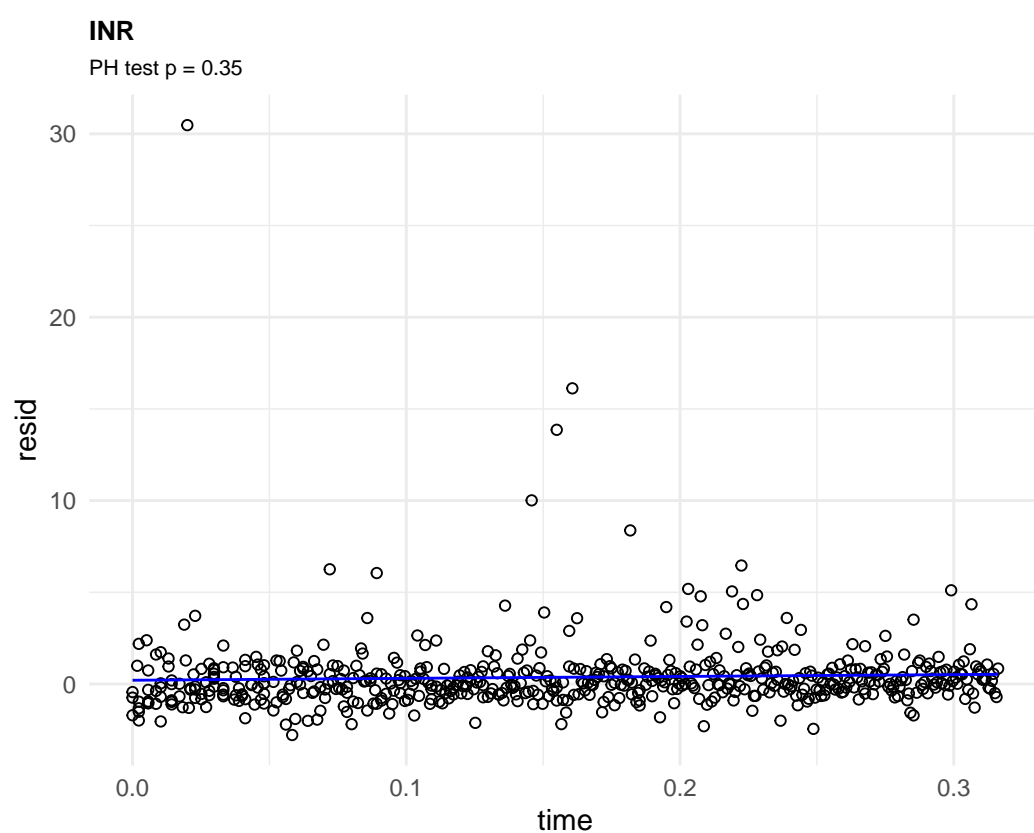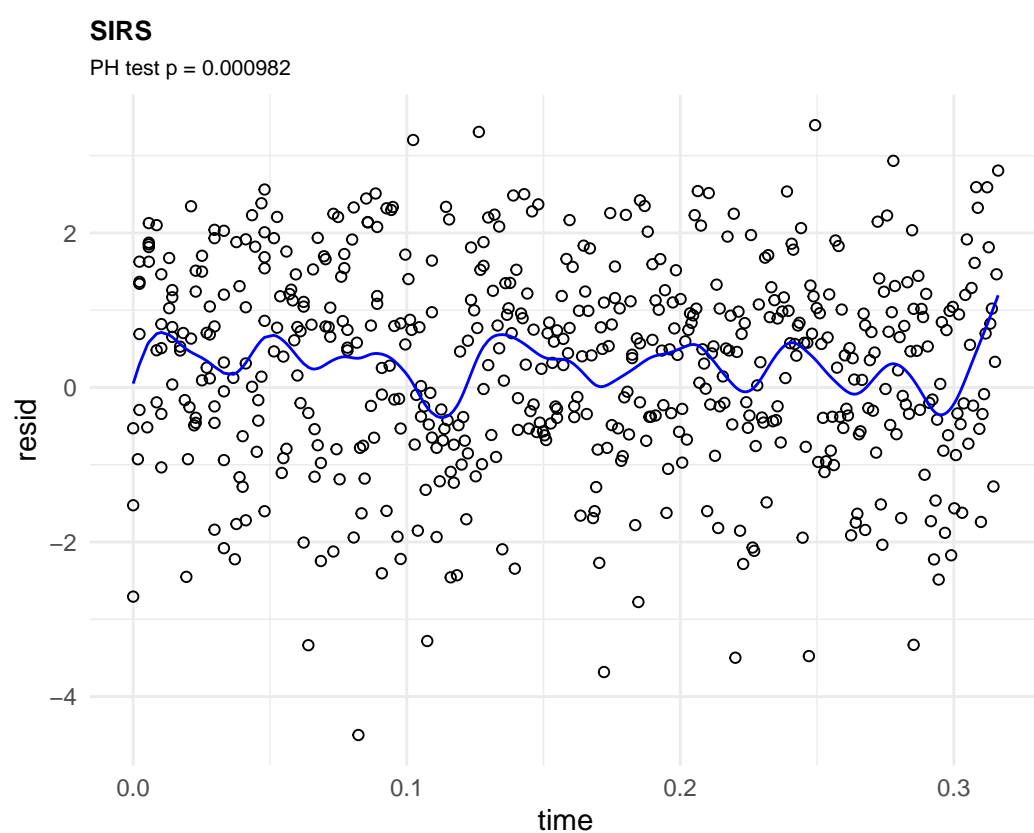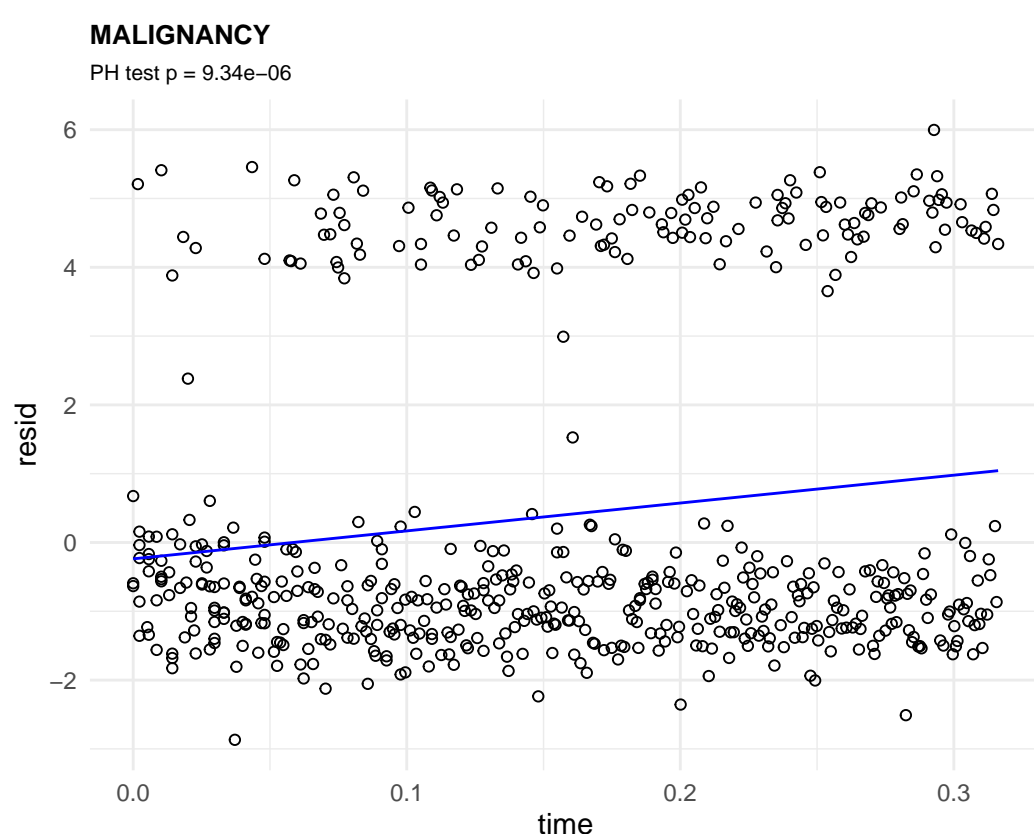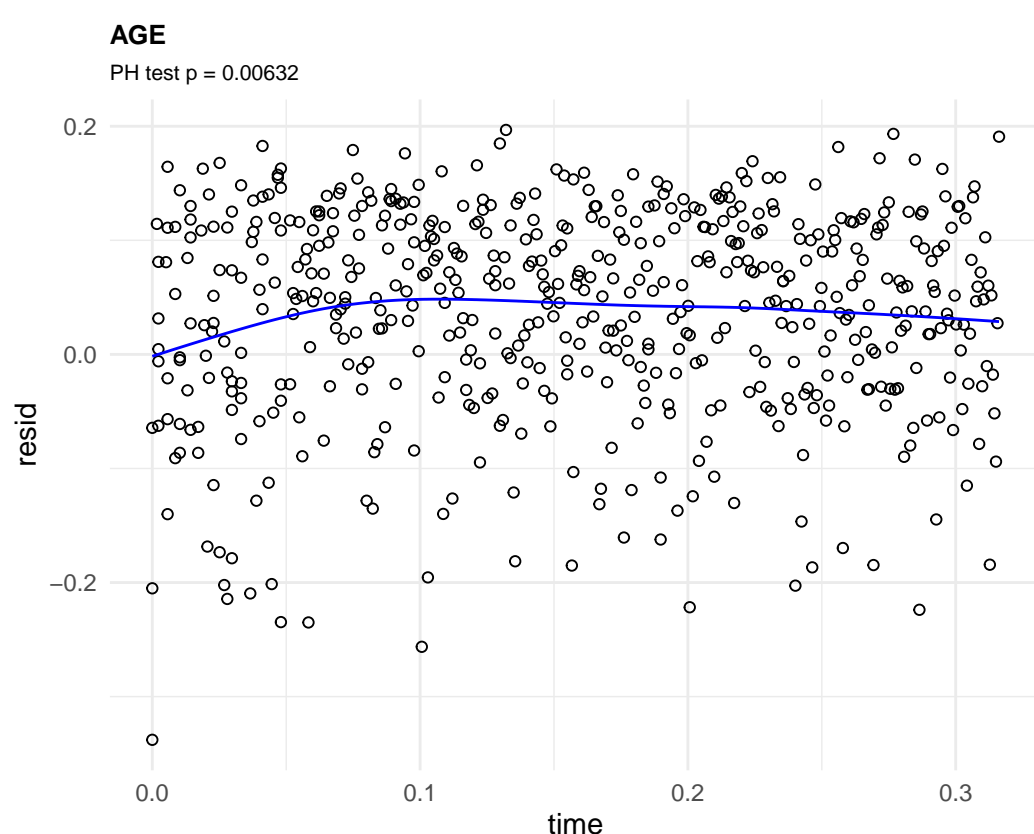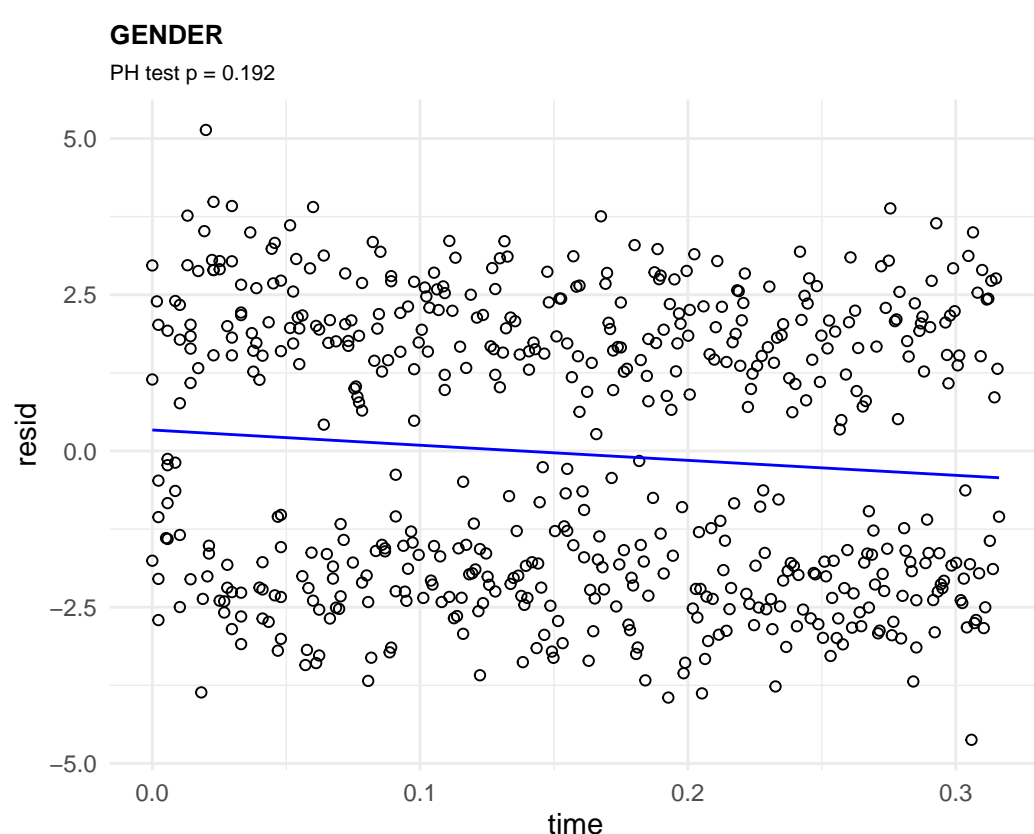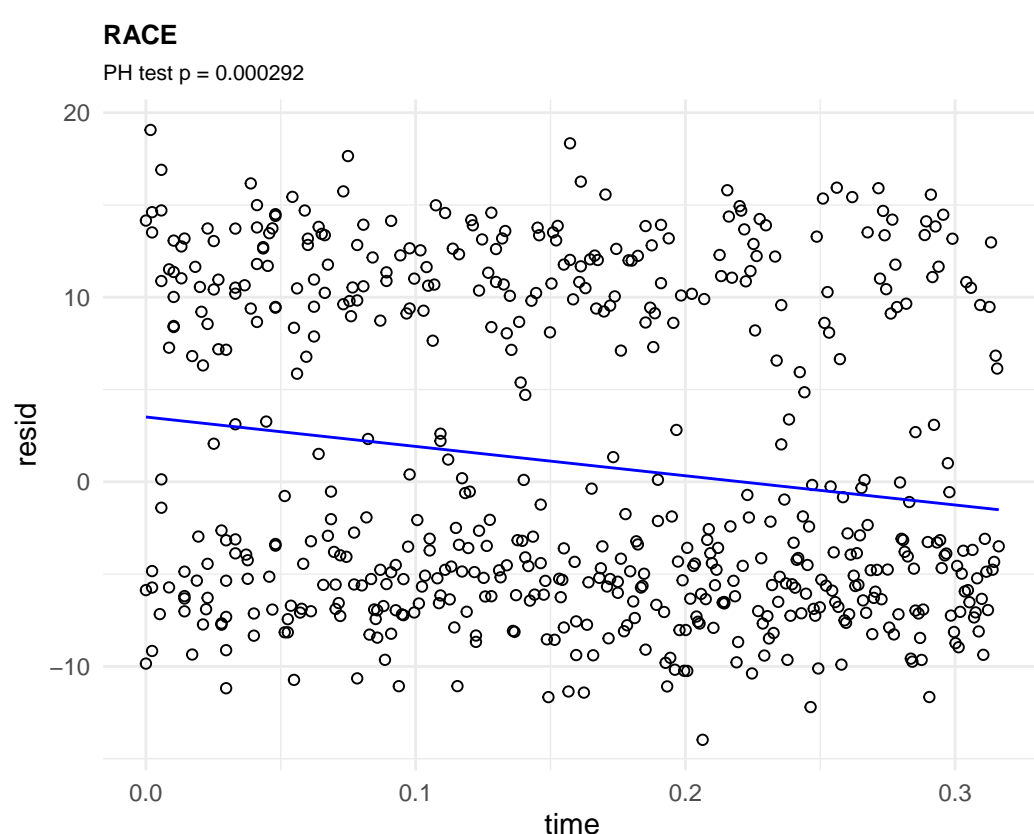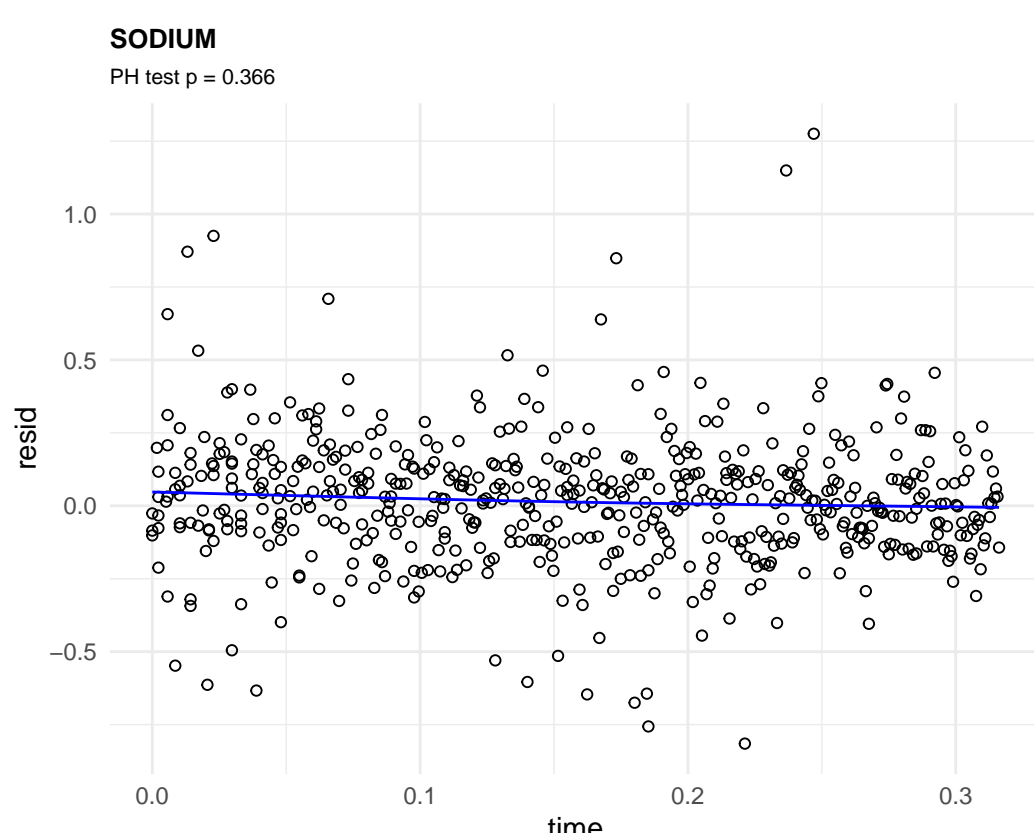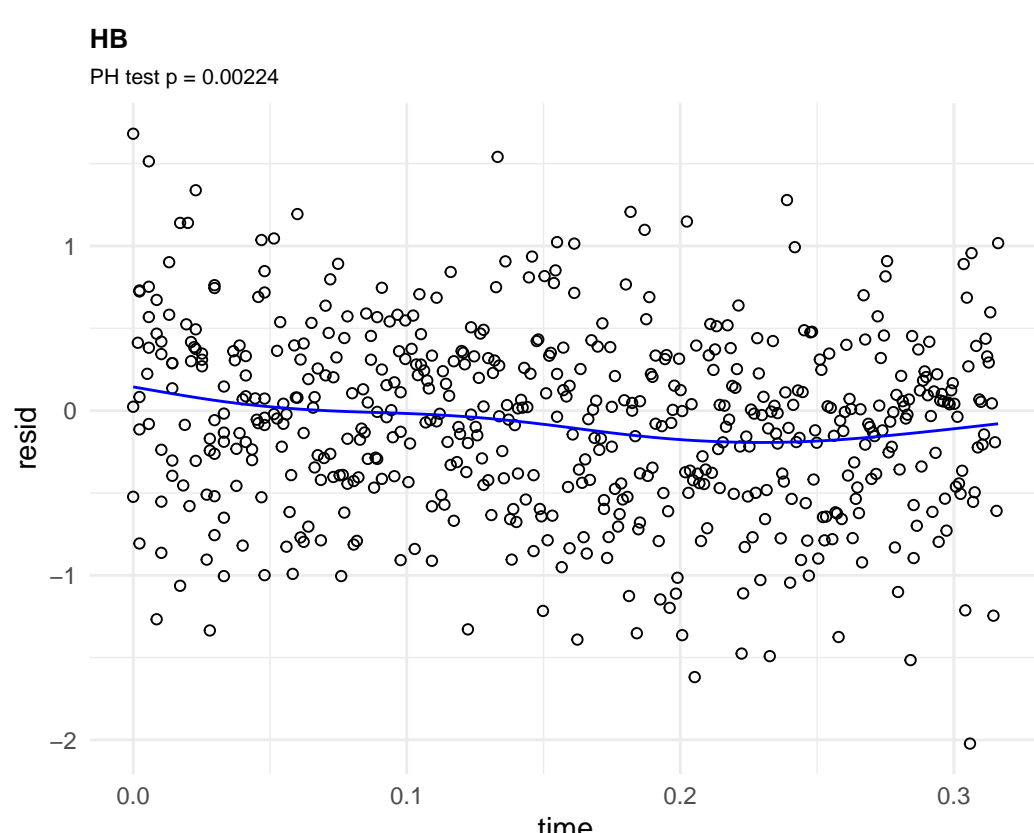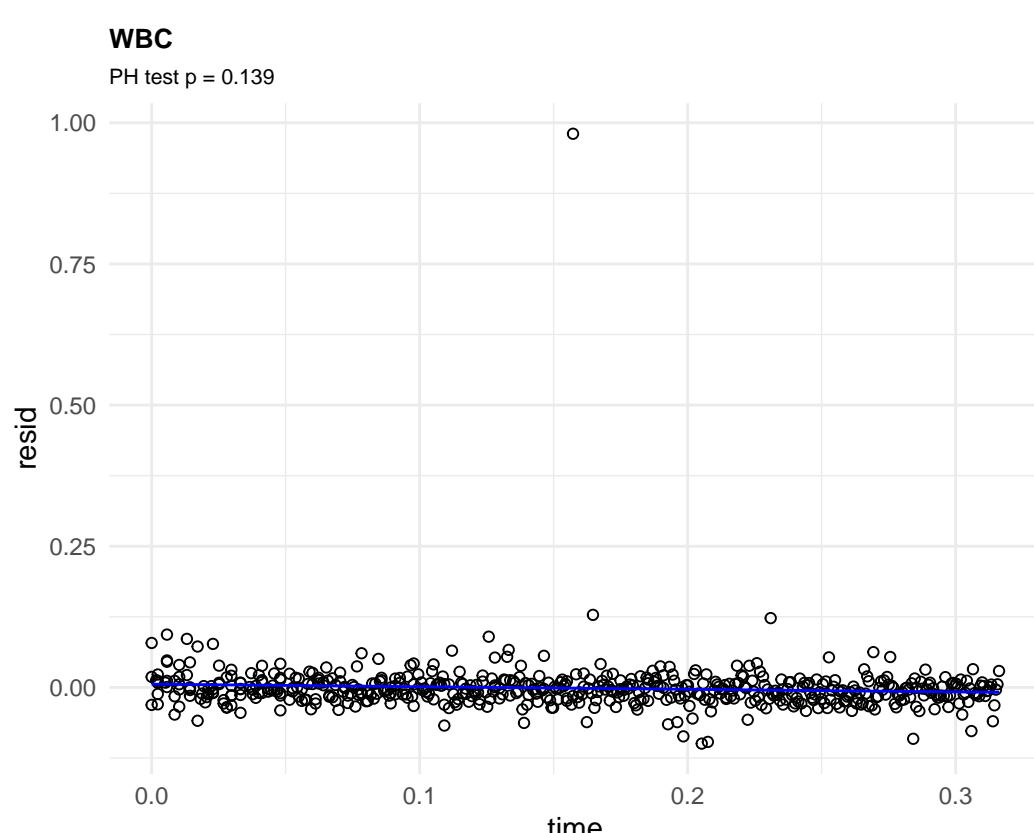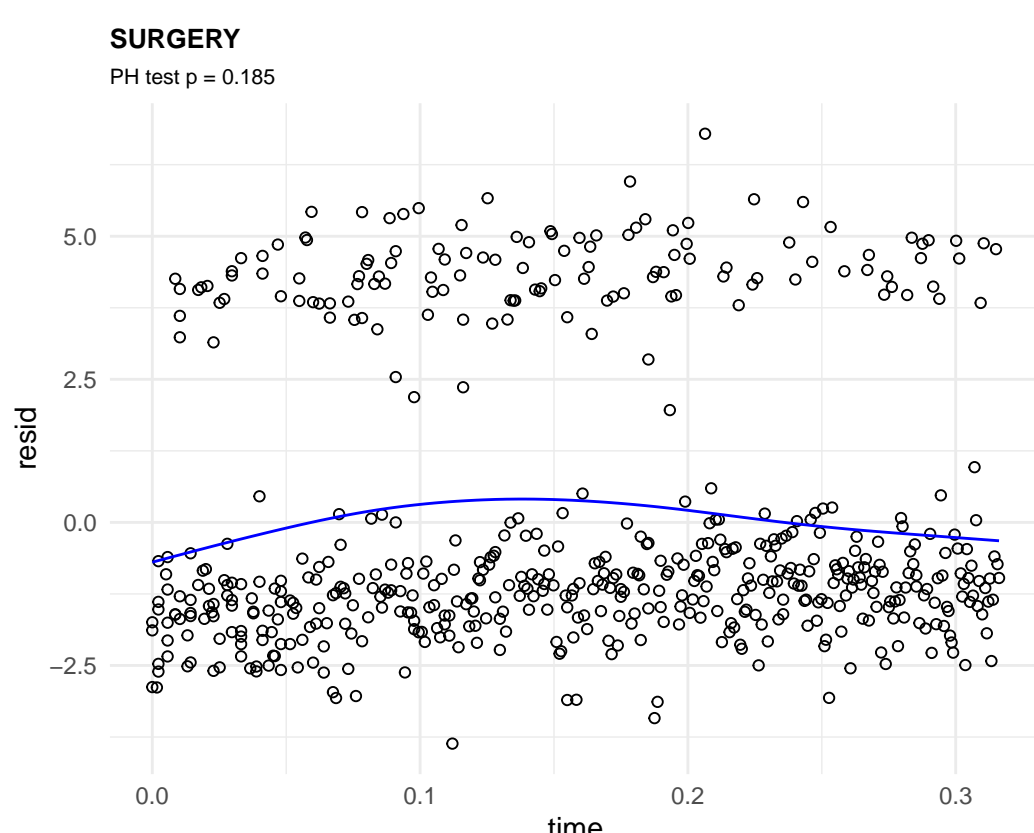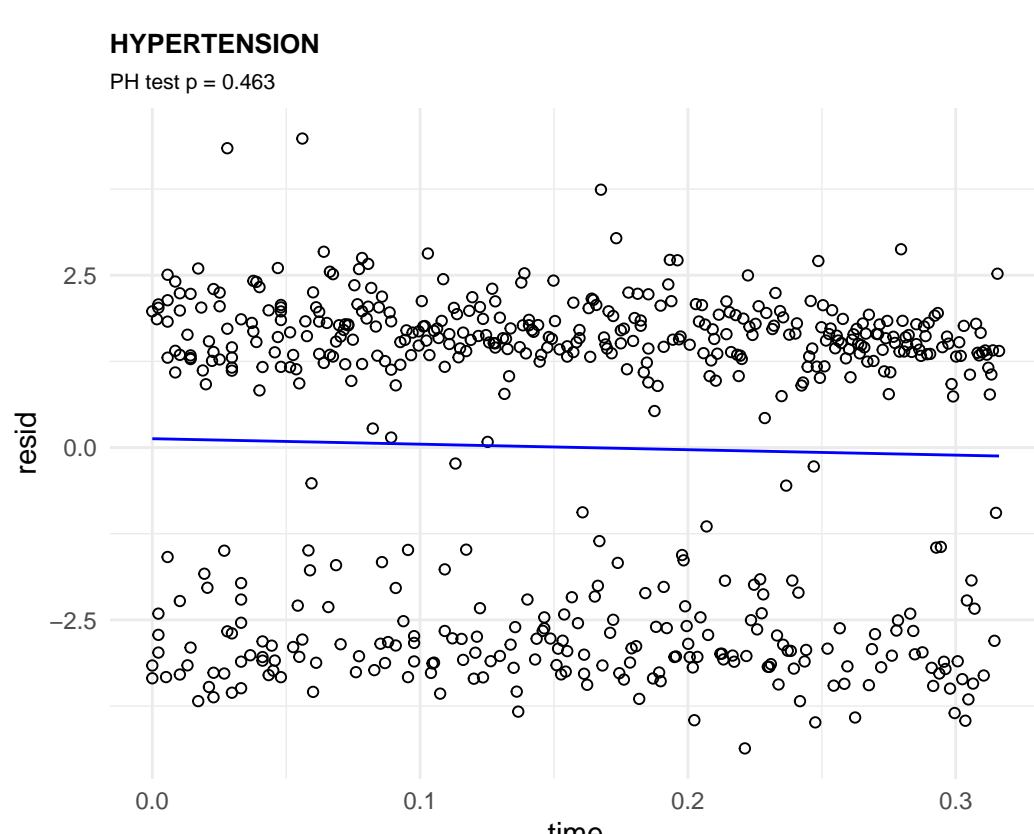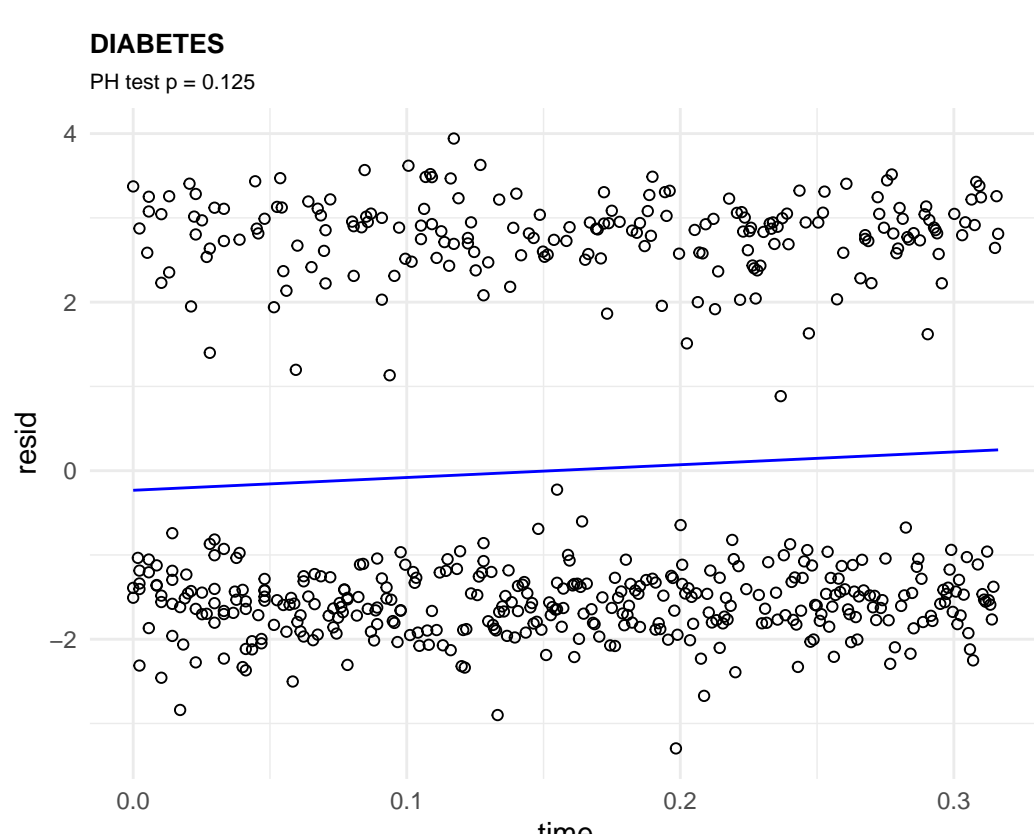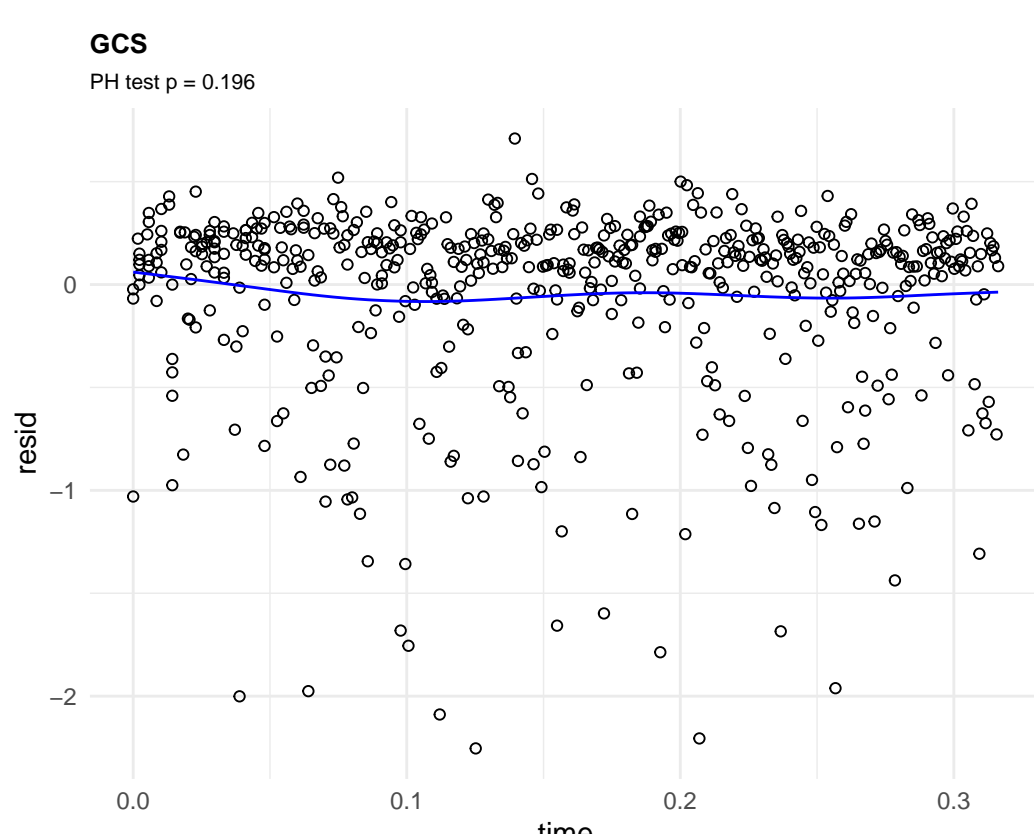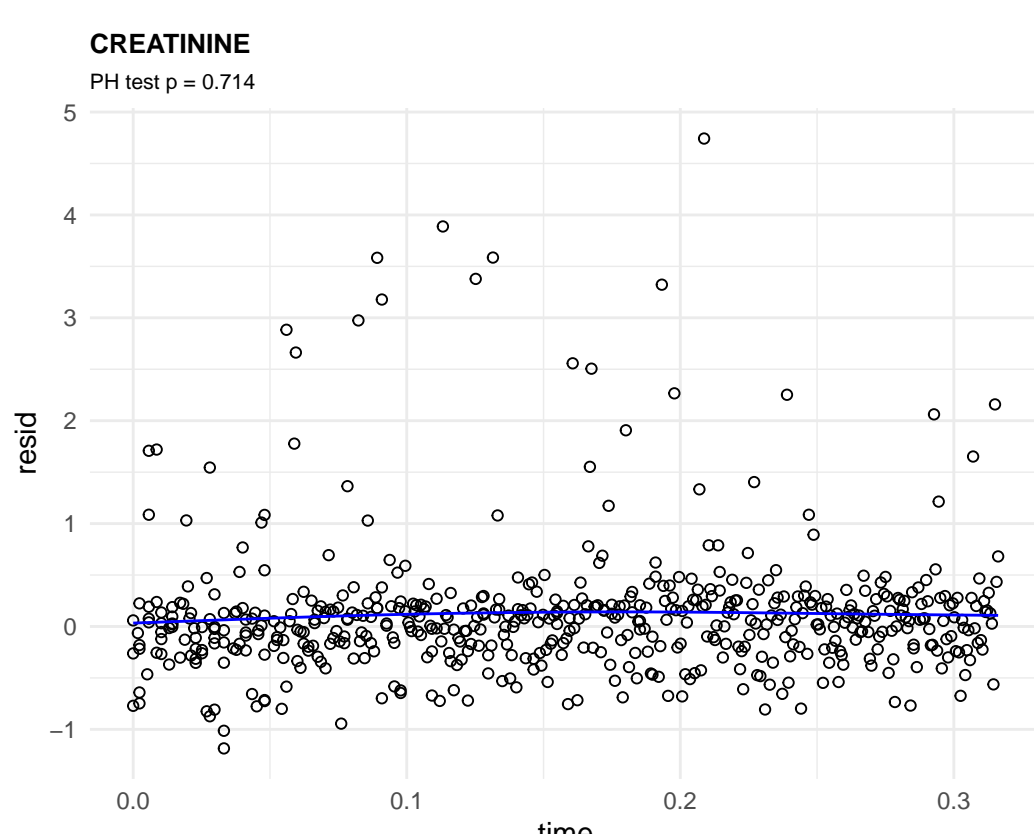

Supplement: Supplementary Figure 6 — Schoenfeld residuals for 1-year mortality model. [file DataSheet6.pdf]
