# Supplementary material for: Unveiling the role of stress hyperglycemia in predicting mortality for critically ill hemorrhagic stroke patients: insights from MIMIC-IV
Source: Front Endocrinol (Lausanne). 2025 May 2;16:1558352. doi: 10.3389/fendo.2025.1558352 (PMC12081252; doi:10.3389/fendo.2025.1558352)
Supplement: Supplementary file 7 [file Table1.docx]

**Table S 1.** Variance inflation factor (VIF) values for variables included in the multivariate cox regression model

| Variable | VIF |
| --- | --- |
| SHR | 1.1 |
| Hemoglobin | 1.07 |
| Age | 1.06 |
| WBC | 1.06 |
| INR | 1.04 |
| GCS | 1.04 |
| Creatinine | 1.04 |
| Sodium | 1 |
